# A Trauma-informed Care Curriculum for Perinatal Providers, Staff, and Learners

**DOI:** 10.15766/mep_2374-8265.11563

**Published:** 2025-12-09

**Authors:** Jennifer Chin, Meghal Sheth, Sam Britz, Lauren Owens, Ying Zhang

**Affiliations:** 1 Assistant Professor, Department of Obstetrics and Gynecology, University of Washington School of Medicine; 2 Resident, Department of Obstetrics and Gynecology, Swedish Medical Center; 3 Resident, Department of Anesthesia, University of Washington School of Medicine; 4 Associate Professor, Department of Obstetrics and Gynecology, University of Washington School of Medicine; 5 Associate Professor, Department of Family Medicine, University of Washington School of Medicine

**Keywords:** Trauma-informed care, Perinatal Health, Clinical/Procedural Skills Training, Obstetrics

## Abstract

**Introduction:**

Trauma, including physical, emotional, and psychological experiences, is highly prevalent and can lead to profound, long-lasting health consequences, especially in perinatal patients, who are uniquely vulnerable to trauma. Research suggests that providers caring for perinatal patients are unprepared to deliver trauma-informed care (TIC). We designed a curriculum for individuals who interact with perinatal patients in TIC principles, practice, and reflection.

**Methods:**

The curriculum was a four-part virtual workshop series delivered over a year to all providers, staff, and learners who interacted with perinatal patients at a large academic medical center. All sessions were 60 minutes and included didactic teaching and small-group case-based discussion. Session 1 introduced the principles of TIC. Session 2 focused on the practice of TIC for perinatal patients. Session 3 reflected on vicarious trauma. Session 4 elevated community voices. We elicited qualitative comments and evaluated self-perceived knowledge, confidence, and self-reported application using 5-point Likert scales pre- and postworkshop, comparing scores using two-tailed paired *t* tests.

**Results:**

In total, 246 participants attended our workshop series. Of these, 34 (14%) completed pre/postworkshop surveys. Self-perceived knowledge about perinatal TIC significantly increased (mean score 3.5 vs 4.6; *p* < .05), belief that TIC was important increased (mean score 4.7 vs 4.9; *p* < .05), and realization that TIC required both individual and systems-level change increased (mean score 4.6 vs 4.8; *p* < .05) pre- to postworkshop. Participants planned to incorporate lessons learned into clinical practice.

**Discussion:**

Our curriculum improved participants’ self-perceived knowledge and preparedness in TIC for perinatal patients.

## Educational Objectives

By the end of this activity, learners will be able to:

1. Define trauma-informed care.
2. Describe trauma-informed care approaches to providing care to perinatal patients.
3. Define vicarious trauma.
4. Explain the importance of partnering with community members to improve trauma-informed care.

## Introduction

Individuals who give birth can experience trauma before, during, and after pregnancy, which has the potential to cause adverse health outcomes for both the birthing person and the infant.^1^ Trauma is defined as an event(s) or circumstance experienced as physically or emotionally harmful or life threatening and has lasting adverse effects on wellbeing, including uncontrolled chronic disease, comorbid mental health conditions, increased risk for undiagnosed sexually transmitted infections, and lower rates of preventive health screening.^2,3^

Traumatic experiences before pregnancy may be related to adverse childhood events that contribute to issues during childbearing years,^3^ as well as sexual, physical, and emotional abuse.^4–6^ Traumatic experiences may also occur in health care settings either before or during birth, with patients experiencing loss of autonomy, being threatened, and receiving inadequate responses to requests for help.^7^ These experiences are often magnified and occur more frequently toward individuals of color.^5,7,8^ Birthing individuals who identify as trans or nonbinary often face traumas such as dysphoria, isolation, exclusion, and poor care within health care settings, which can make them more susceptible to perinatal mental health problems and traumatic birth experiences.^9^

Nine percent of childbearing individuals in one study reported experiencing posttraumatic stress disorder, with another 18% considered at high risk.^10^ Trauma-informed care (TIC) frameworks aim to prevent re-traumatization by focusing on clinician skills and systemic changes to support patient wellbeing.^2^ Many TIC training programs use didactic methods to teach TIC principles to health care providers, including medical residents, with some programs incorporating skills practice using standardized patients to enhance competency.^11–14^ Several previously published curricula have focused on TIC for medical students, pediatricians, military-connected individuals, and emergency medicine learners.^15–18^ None of these curricula specifically focused on caring for perinatal patients. Our study is unique in that our training program was specifically designed to enhance the care of perinatal patients by integrating case-based learning within multidisciplinary groups and promoting interdisciplinary discussion and problem-solving. Our approach fosters diverse dialogue by incorporating patient perspectives and engaging community partners, ensuring a more comprehensive and patient-centered educational experience.

While TIC training is often generalized, a subset of studies has focused on TIC training for providers giving care and support to pregnant individuals, to improve postpartum mental health outcomes.^19^ Researchers recognize that many providers lack TIC education and acknowledge its potential benefits for perinatal care.^20^ Two quantitative studies on TIC programs for perinatal caregivers, including nurses, doulas, and midwives, reported improved knowledge about TIC after receiving training.^11,21^ A module on assessing trauma history in pregnant patients, previously published in *MedEdPORTAL*, did not incorporate a multidisciplinary approach nor did it include information about how to perform trauma-informed pelvic exams.^22^ Another module used standardized patients in a multidisciplinary simulation to improve knowledge and comfort regarding providing TIC for pregnant patients impacted by a prior traumatic event.^23^ However, this module did not incorporate information on vicarious trauma or community voices and reflections.

Our curriculum engages a multidisciplinary group of staff and clinicians in TIC training, including attending physicians, residents, medical students, nurses, midwives, social workers, medical assistants, and front desk personnel who interact with perinatal patients across clinical settings. Our curriculum was delivered through a synchronous virtual platform, using Zoom, to broadly reach staff and clinicians across multiple hospital and clinic locations. We purposefully combined didactics learning with small-group discussions for our curriculum, to facilitate multidisciplinary engagement and real-time reflection on the educational material. Prior to and after the training workshops, we assessed participants’ self-perceived knowledge, confidence, and intention to implement TIC principles in practice in their perinatal work settings. In accordance with Level 2 of the Kirkpatrick training evaluation model, we assessed participants’ immediate postworkshop self-perceived knowledge and confidence with various aspects of perinatal TIC, as well as participants’ intention to implement TIC principles in practice, providing insight into potential shifts in practice behavior and long-term improvements in patient care.^24,25^

## Methods

### Curriculum Description

We developed our four-part workshop series for providers, staff, and learners across all clinic sites and two hospital campuses of our academic hospital system in 2022. Each workshop combined didactics learning with interdisciplinary small-group discussion and reflection on learning and practice experiences. The Schon Theory of reflective learning, which includes knowledge in action, reflection in action, and reflection on action, influenced our curriculum design, allowing us to deliver a workshop series focused on practice-based learning and highlighting experiential knowledge of participants and instructors.^26^ Reflection in action occurred during the workshops through small-group breakout discussions, while reflection on action was promoted through the longitudinal nature and repeating discussion-group breakout sessions at each subsequent workshop of the series.^27,28^ We then administered the training in four separate 1 hour–long virtual sessions, each offered in the morning and afternoon with the same content, to all learners who interacted with perinatal patients from 2022 to 2023. Our first session covered an overview of TIC (Appendix A); our second session covered TIC in perinatal care (Appendix B); our third session covered vicarious trauma (Appendix C); and our fourth session covered community voices and reflections (Appendix D). The workshops were developed as a collaboration among labor and delivery nurses, obstetrics and gynecology (OB/GYN) physicians, a Family Medicine physician, an Infectious Disease physician, and a psychotherapist specializing in posttraumatic stress disorder. The University of Washington Institutional Review Board (IRB) granted the study exempt status (August 15, 2022; IRB No. STUDY00016147).

### Materials

The training curriculum required internet access and was carried out virtually on the Zoom platform. Electronic and physical fliers publicizing each session were used to gather eligible participants. The surveys were administered using REDCap.^29,30^

### Faculty

Each workshop featured two to four providers (ie, family medicine physician, behavioral health licensed therapist, OB/GYN physician, or infectious disease physician) who delivered the didactic material, and an additional three to four moderators (ie, other physicians or nurses on the planning team who were not presenters) who led the small-group discussions and monitored the online chat. The presenters and facilitators had previous experience delivering and teaching TIC in clinical contexts. Our final workshop included community members and patient voices with professional and/or personal experience surrounding TIC in the perinatal space. This final workshop did not have official breakout sessions but did leave space at the end of the presentation for a debrief among participants.

### Participant Preparation and Safety Measures

Considering the sensitive topic of this training, we took various precautions to prepare participants for the sessions. Prior to the first workshop, we emailed participants a detailed description of the topics we would cover. At the beginning of all workshops, we presented a slide with our academic center's specific resources for mental health, spiritual care, and survivor resources. Our goal was to avoid subjecting participants to potentially triggering content without providing them advance warning. We notified participants that they could go off-camera or leave the workshop at any point if they felt uncomfortable. Given the sensitive nature of the topics and the likely prevalence of trauma, even among our learners, breaking into small groups to discuss cases was a way to allow for discussion in more intimate, less intimidating ways.

### Part 1: TIC and Approach to Care

Our team created and delivered a 1-hour live virtual session in which we provided background information on the definition of trauma, its impact on long-lasting health, and principles of TIC (Appendix A). Topics were divided among faculty based on content expertise. We ended the session with small-group breakouts to discuss how trauma showed up for participants’ day-to-day work with patients, in what ways their clinical spaces were trauma-informed, in what ways their clinical spaces were not trauma-informed, and how we could address those differences. One of our faculty who was not presenting created breakout groups of three to four participants, with a faculty member assigned to each breakout group. Within the breakout groups, we first discussed group norms and ground rules, and then proceeded with discussion questions and sharing. After this, we brought all participants back into one large group and asked each group to share something they discussed. We spent 30 minutes on didactics, 20 minutes on breakout groups and discussion, and 10 minutes for closing and questions.

### Part 2: TIC in Perinatal Care

Next, our team created and delivered a 1-hour live virtual session in which we discussed why TIC was particularly important in perinatal care and explored applications in outpatient and inpatient perinatal care (Appendix B). Topics were divided among faculty based on content expertise. We led small-group case-based discussions to apply lessons learned on TIC for pregnant individuals in the outpatient and inpatient setting. Breakout rooms used the same structure as described in Part 1 for moderation, ground rules, discussion, and sharing back to the large group. We spent 30 minutes on didactics, held two separate 10-minute breakout group and discussion sections, and left 10 minutes for closing and questions.

### Part 3: Vicarious Trauma

Next, our team created and delivered a 1-hour live virtual session in which we discussed the definition of vicarious trauma and recognized trauma exposure responses in participants and coworkers (Appendix C). Topics were divided among faculty based on content expertise. We led small-group breakouts to discuss best practices for managing these responses and ways we could change our systems to support integration of trauma stewardship. Breakout rooms used the same structure as described in Part 1 for moderation, ground rules, discussion, and sharing back to the large group. We spent 30 minutes on didactics, held two separate 10-minute breakout groups and discussion sections, and left 10 minutes for closing and questions.

### Part 4: Community Voices and Reflections

In our final session, we invited four community members to share their experiences with us about their personal and professional experiences with trauma and TIC (Appendix D). These panelists included staff and a doula for a community-based perinatal support organization, staff at a local community center supporting clients affected by substance use disorder (SUD), and an individual with a history of SUD who gave birth in our hospital system. When recruiting members of the panel, we consulted our planning group to identify patient communities of interest, including Black or Indigenous people of color (BIPOC) communities and families or communities affected by SUD. Based on their guidance, we conducted outreach through key contacts recommended by our planning committee and recruited panel members from these connections. We conducted meetings with each of these groups prior to our sessions to gauge what questions individuals felt comfortable answering, how they wanted the session to be conducted, and if there were specific messages they wanted to convey to our participants. Our morning session featured individuals with SUD and birth workers supporting them. Our afternoon session featured Black birthing patients. We asked community members to share their personal and professional interactions with the health care system, in what ways the care they received was trauma-informed, and their suggestions for improvement when it was not trauma-informed. We spent 50 minutes with our community members, including 10 minutes for questions, and left an additional 10 minutes after our community members left for participants to debrief and reflect. We provided stipends to these community members to thank them for their time and vulnerability.

### Participant Survey Development and Structure

We invited everyone who attended to fill out a preworkshop survey by including a survey link and QR code in our invitation emails and the initial slide of all our presentations (Appendix E). This survey was developed by exploring our educational objectives and involved collaboration from our entire planning team, who piloted the survey prior to implementation. We then invited all participants who completed a preworkshop survey to complete a postworkshop survey 1 month after our series ended (Appendix F). We chose a 1-month gap before administering the postworkshop survey in order to assess the participants’ long-term retention of information rather than immediate attainment of the educational outcomes, as our goal was to influence long-term patient care. We did not calculate a sample size as we wanted to gather as many responses as possible. Our target population was anyone within our institution who interacted with perinatal patients and included providers, staff, and learners. All participants were given a written description of the study and were invited to fill out an optional pre- and postworkshop survey, and all participants provided written consent prior to completing each survey.

We surveyed participants for self-perceived knowledge, confidence, and plans to use strategies presented in this program by administering surveys immediately before the first completed session and 1 month after our final session (Appendix E and Appendix F). To ensure accurate comparison, we asked each participant to provide their email when filling out the preworkshop survey and to enter the same email for their postworkshop survey. The surveys featured 15 questions that assessed participant self-perceived knowledge and confidence with various aspects of TIC on a 5-point Likert scale (1 = *strongly disagree*, 2 = *disagree*, 3 = *neutral*, 4 = *agree*, and 5 = *strongly agree*). We also included three free-text questions in the postworkshop survey: (1) “Did this program meet your personal objectives?”; (2) “What barriers do you perceive will hinder your ability to implement the knowledge gained from this workshop?”; and (3) “Have you incorporated anything from these trainings into your practice?” We categorized participant feedback by analyzing recurring themes throughout participant responses. Participants were offered a $10 gift card for each completed pre- and/or postworkshop survey.

### Survey Data Analysis

We collected survey data using REDCap, and then extracted and analyzed the data using Microsoft Excel. We compared the pre- and postworkshop Likert scores from matched survey responses using paired *t* tests. We calculated the mean with interquartile range for the composite pre- and postworkshop survey scores for each participant. We then calculated the difference between the composite pre- and postworkshop scores using paired Student's *t* tests, and defined our significance level as a *p* value <.05. We did not include unpaired survey responses in the analyses.

## Results

In total, 246 perinatal providers, staff, and learners attended our workshop series. Of these participants, 34 (14%) completed pre- and postworkshop evaluation surveys (Table 1). Of those who completed pre- and postworkshop evaluation surveys, 14 (41%) attended all four sessions, and four (29%) of these 14 participants were physicians and medical learners.

**Table 1.**
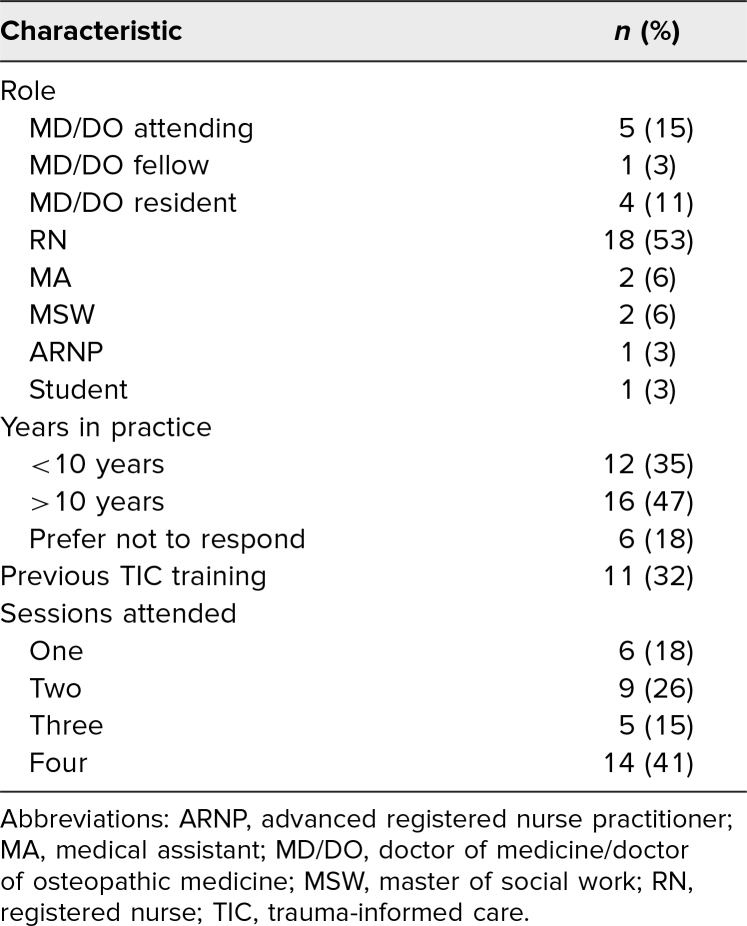
Characteristics of Survey Responders Participating in the TIC Workshop (*N* = 34)

### Effectiveness of the Intervention

For each of the six survey questions assessing self-perceived knowledge, we compared participants’ pre- and postworkshop Likert scores from matched survey responses. Of the 14 participants who completed both surveys and attended all four sessions, we found a significant increase from pre- to postworkshop in self-perceived knowledge of perinatal TIC (mean score 3.5 vs 4.7; *p* < .05), identification of posttraumatic stress (3.6 vs 4.4, *p* = .02), how to address traumatic situations in labor for someone with a trauma history (3.4 vs 4.2, *p* < .05), identification of vicarious trauma (3.5 vs 4.2, *p* = .02), and how certain populations disproportionately experience trauma (3.7 vs 4.4, *p* = 0.03).

When looking at survey responses from all participants including those who attended fewer than four sessions (*N* = 34), we demonstrated a statistically significant increase in self-perceived knowledge and confidence in each of the six assessed knowledge areas regarding perinatal TIC (all *p* < .01 vs preworkshop), as shown in Table 2. Postworkshop, participants were more likely to note the importance of practicing TIC and realized that it required both individual and systems-level change (each *p* < .05 vs preworkshop). Most of our survey respondents planned to use strategies presented in the program to enhance their care for future patients.

**Table 2.**
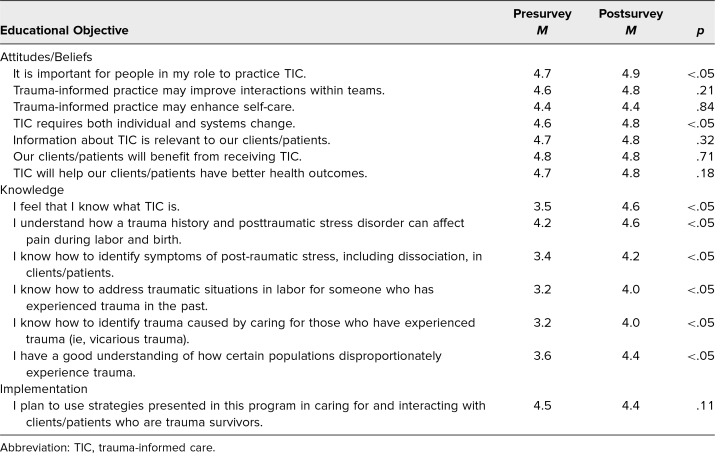
Participants’ Pre- and Postworkshop Survey Ratings of Attitudes, Knowledge, and Plans (*N* = 34)

We categorized participant feedback from the three free-text questions into three primary categories: personal objectives, barriers to implementation, and practice changes. Twenty-three participants responded to our first question (personal objectives), of whom 15 responded that the trainings did meet personal goals. Some of these goals included increasing understanding of TIC, as illustrated by a participant stating, “It was a very informative overview and provided information for everyone to incorporate into our interactions and practices.” Another goal was identifying trauma and communicating and engaging patients with trauma, as illustrated by a participant stating, “Reviewing the direct implications to our patient population was helpful.” An additional goal was being able to address trauma with colleagues and oneself, as illustrated by a participant stating, “It is helpful to listen to how different attendings approach the topic.”

For our second free-text question (barriers to implementation), 20 participants commented on barriers to implementation of TIC, and themes included systems-based challenges such as access to resources, and obstacles related to insurance practices; as one participant noted, “needing to bill insurance.” Other barriers included management and policies (*n* = 2) and not having enough time in patient interactions (*n* = 4). One participant noted, “The business of my schedule will be a barrier.” Another barrier noted was not everyone on the care team having appropriate knowledge or motivation for TIC (*n* = 5), as illustrated by a participant stating, “Other team members not being trained on TIC skills.”

For our third free-text question (practice changes), 28 participants responded that they have incorporated trainings into practice, and 17 named specific components, such as being mindful of language and nonverbal cues (*n* = 6), as illustrated by a participant stating, “I have universal phrases that I use with every patient.” Others noted having different approaches and modalities for performing physical exams (*n* = 3), as illustrated by a participant stating, “I use the stirrups less. I ask every patient about past experiences prior to an exam.” Finally, comments from some participants indicated that they learned to generally approach patients with respect and assume that every patient has a trauma history (*n* = 5).

## Discussion

Trauma is a prevalent problem with detrimental, long-term health consequences.^31^ TIC and trauma-informed approaches to care are increasingly recognized as important principles to follow for all populations, but this is particularly important for perinatal patients.^15^ Prior curricula have focused on providers and medical students, whereas our work intentionally included nonmedical staff who interact with perinatal patients.^15–18,22,23^ This was a strength of our workshop, as we were able to include a broader audience to incorporate multiple perspectives in our breakout discussions. At baseline, we found that perinatal providers, staff, and learners acknowledged the importance of TIC when interacting with patients. We demonstrated the effectiveness of our curriculum on improving the self-perceived knowledge and confidence of incorporating TIC for perinatal patients. Our curriculum was successfully implemented using widely available resources, including Zoom, PowerPoint presentations, and small-group breakout discussions.

Our data showed improvements in self-perceived knowledge scores as well as increased confidence and comfort in providing TIC for perinatal patients. We purposely administered postworkshop surveys 1 month after completion of the workshop to ensure participants retained the information gained from the workshop series. However, this may have introduced confounding factors such as other learning experiences that may have influenced participants’ perceptions. The best way to maintain the long-term benefits of this curriculum is a potential area for future study.

Our survey responses showed that most participants self-reported incorporating components of our training into practice, including using intentional language and nonverbal communication, performing sensitive and patient-centered physical exams, and assuming all patients have some type of trauma history.

### Limitations and Future Directions

Our work shows that a curriculum about TIC for perinatal patients can have a significant impact on self-perceived knowledge, confidence, and self-reported plans to incorporate knowledge gained into future practice. We demonstrated benefits with only 4 hours of teaching time over the course of 1 year. These findings may translate into greater awareness and delivery of TIC for all patients, particularly perinatal patients, more intentional processing of vicarious trauma among providers and staff, and further engagement with community members and organizations.

Due to scheduling constraints, more than half of our respondents were unable to participate in our full curriculum, and some only participated in one of four sessions. We chose to administer our pre- and postworkshop surveys to anyone who completed any part of the curriculum in order to gain as much information as possible about the impact of our curriculum. Future iterations of this program could consider making the full curriculum mandatory or only surveying participants who were able to attend all four sessions. We decided to offer gift cards to participants to thank them for their time and to encourage survey responses. However, offering compensation for completing the surveys may have biased responses by incentivizing those who otherwise would not have responded to our surveys.

Our evaluation of our intervention is limited by a low survey response rate at a single institution and a short time interval; future directions may include applying the curriculum to a broader sample size and repeating surveys at a later time interval. To increase survey participation, perhaps future iterations should employ truly anonymous data collection without email addresses to encourage more participation. To promote sustained behavior change, there could be additional teaching sessions to remind learners about TIC for perinatal patients at later points in their career. The average response to our question about planning to incorporate strategies into future practice unfortunately decreased between the pre- and postworkshop surveys, and the difference in these responses did not reach statistical significance. However, we did note significant changes in responses to our other survey questions. Another significant limitation was that participant behaviors were self-reported and mostly involved practicing clinicians with few trainees, although we invited all providers, learners, and staff to participate. As our assessments of knowledge, confidence, and incorporation of knowledge in clinical practice were all self-reported, we do not know in an objective manner if these areas truly improved. Future directions could include assessing specific pieces of knowledge or measuring concrete impacts of behavior change, such as asking for patient feedback and experiences with receiving TIC from providers, staff, and learners. We could also ask about personal experiences with trauma or vicarious trauma to see whether there would be differences in outcomes after the curriculum. While we conducted our sessions over Zoom to limit physical contact due to COVID-19 restrictions, these sessions could be conducted in person with hands-on elements such as skills stations for obstetric emergencies and high-risk trauma scenarios and simulation-based sessions targeting communication, teamwork, and role play. This approach may improve the engagement of participants as well as provide a more comprehensive and practice-oriented learning experience. We would recommend, if feasible, still holding a morning and afternoon session, as this increased accessibility for providers who often work day or night shifts. It is also possible that providing this training in person may ensure higher participation in pre- and postworkshop surveys, if surveys are administered at the time of training.

Identifying and recruiting workshop participants for our fourth session required significant planning and could be a potential challenge when replicating this session. Our planning committee helped us identify a list of potential panelists through community networks and connections from individual committee members. Some individuals felt uncomfortable sharing their experiences within a large group and thus declined the invitation. We were able to offer a stipend to all participants, which we felt was necessary to compensate them for their participation and vulnerability. We recommend generating a list of potential speakers at least 3 months prior to the session to give adequate time to recruit and prepare speakers and to ensure readiness and comfort with the format of the session and potential questions during the presentation.

Our curriculum extends prior curricula related to TIC to specifically focus on those interacting with perinatal patients, who represent a particularly vulnerable population. Successfully training providers, staff, and learners at our institution is one important step in promoting the best possible care for perinatal patients, regardless of their history of trauma.

## Appendices


Part 1 - Overview of TIC.pptxPart 2 - TIC in Perinatal Care.pptxPart 3 - Vicarious Trauma.pptxPart 4 - Community Voices & Reflection.pptxPresurvey.docxPostsurvey.pdf
*All appendices are peer reviewed as integral parts of the Original Publication.*
