# Supplementary material for: A Trauma-informed Care Curriculum for Perinatal Providers, Staff, and Learners
Source: MedEdPORTAL. 2025 Dec 9;21:11563. doi: 10.15766/mep_2374-8265.11563 (PMC12686155; doi:10.15766/mep_2374-8265.11563)
Supplement: Supplementary file 1 — Part 1 - Overview of TIC.pptxPart 2 - TIC in Perinatal Care.pptxPart 3 - Vicarious Trauma.pptxPart 4 - Community Voices & Reflection.pptxPresurvey.docxPostsurvey.pdf [file mep_2374-8265.11563-s001.zip › A. Part 1 - Overview of TIC.pptx]

## Slide 1
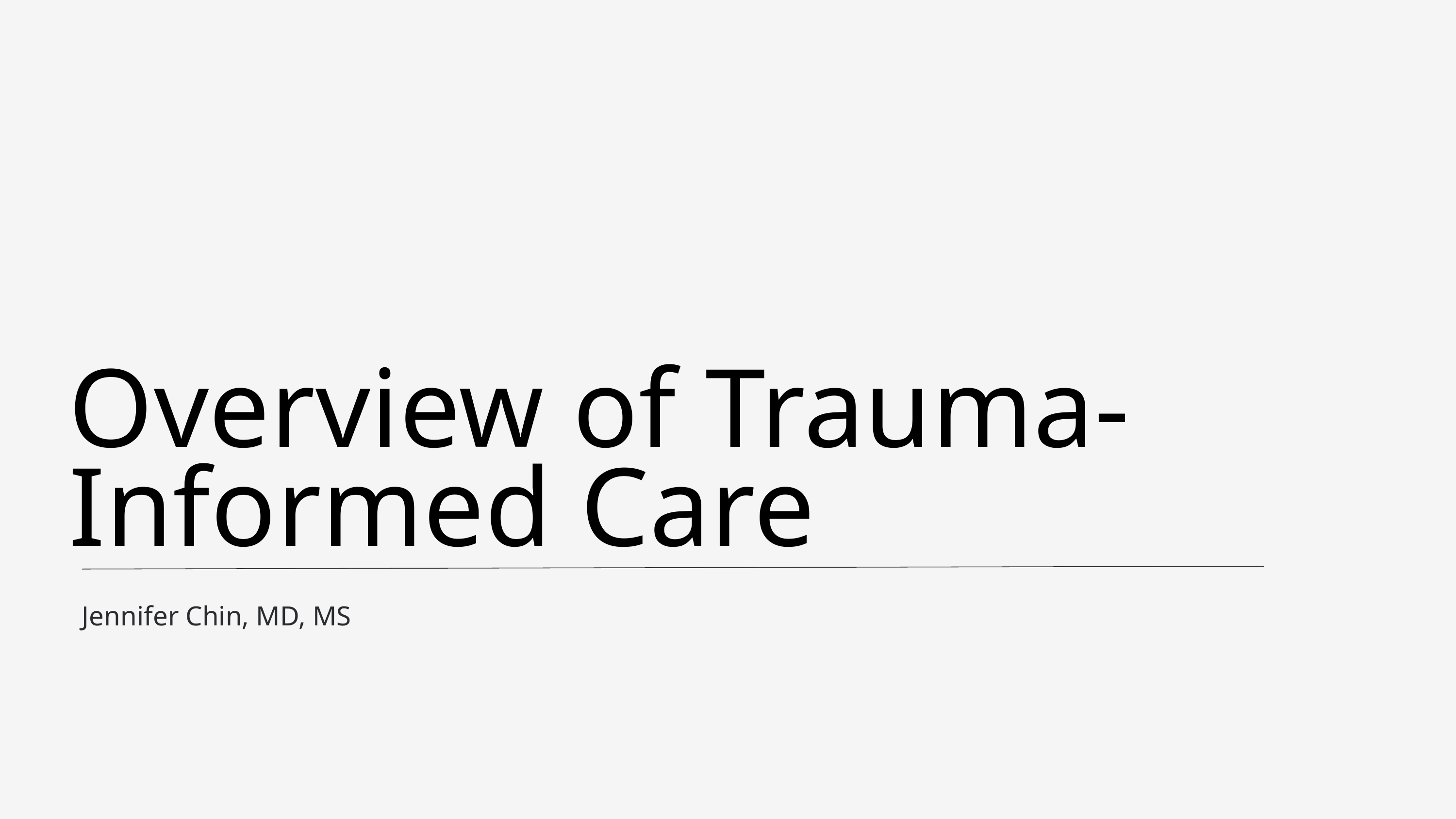

Overview of Trauma-Informed Care
Jennifer Chin, MD, MS

## Slide 2
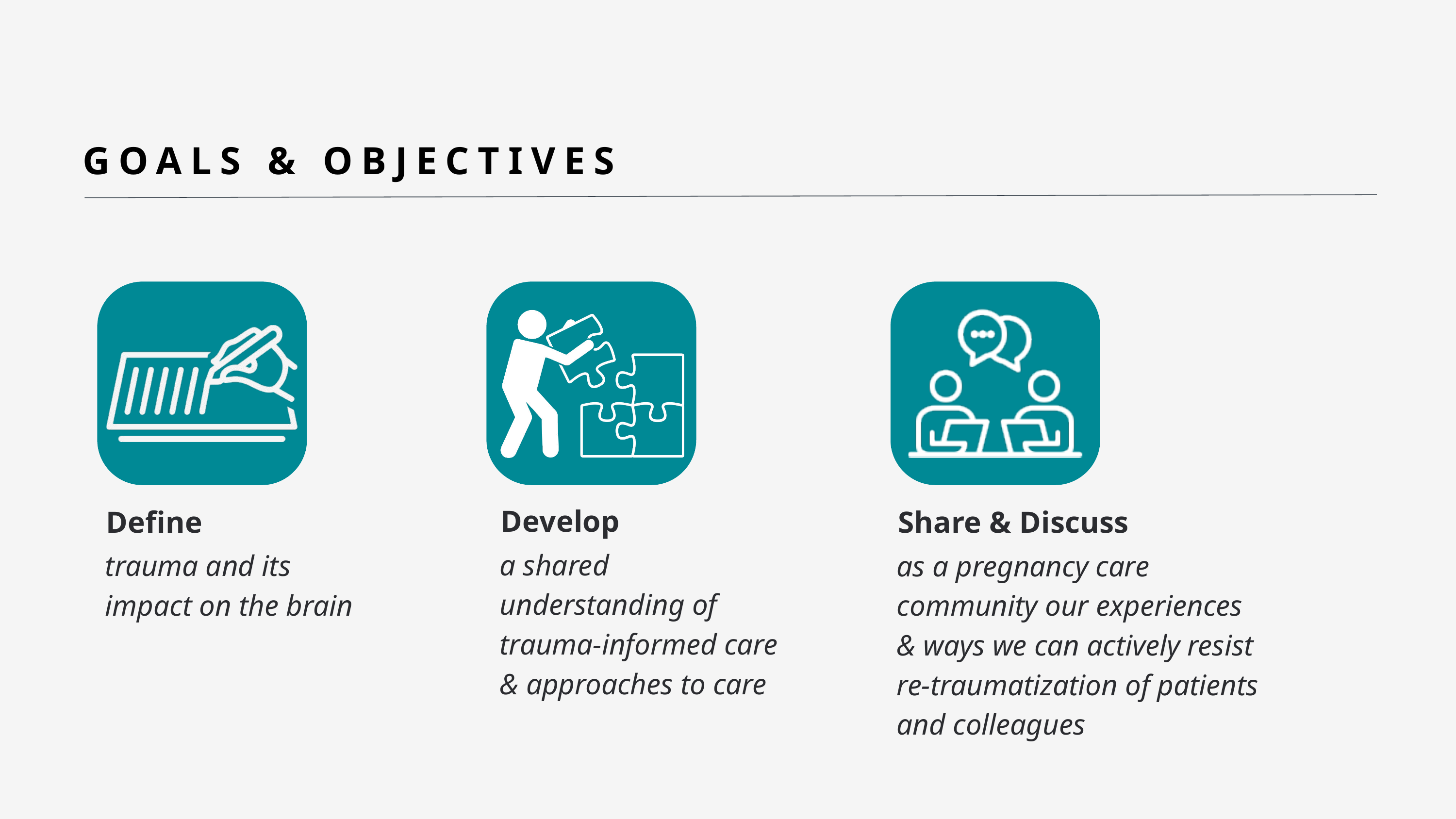

GOALS & OBJECTIVES
Develop
Define
Share & Discuss
a shared understanding of trauma-informed care & approaches to care
trauma and its impact on the brain
as a pregnancy care community our experiences & ways we can actively resist re-traumatization of patients and colleagues

## Slide 3
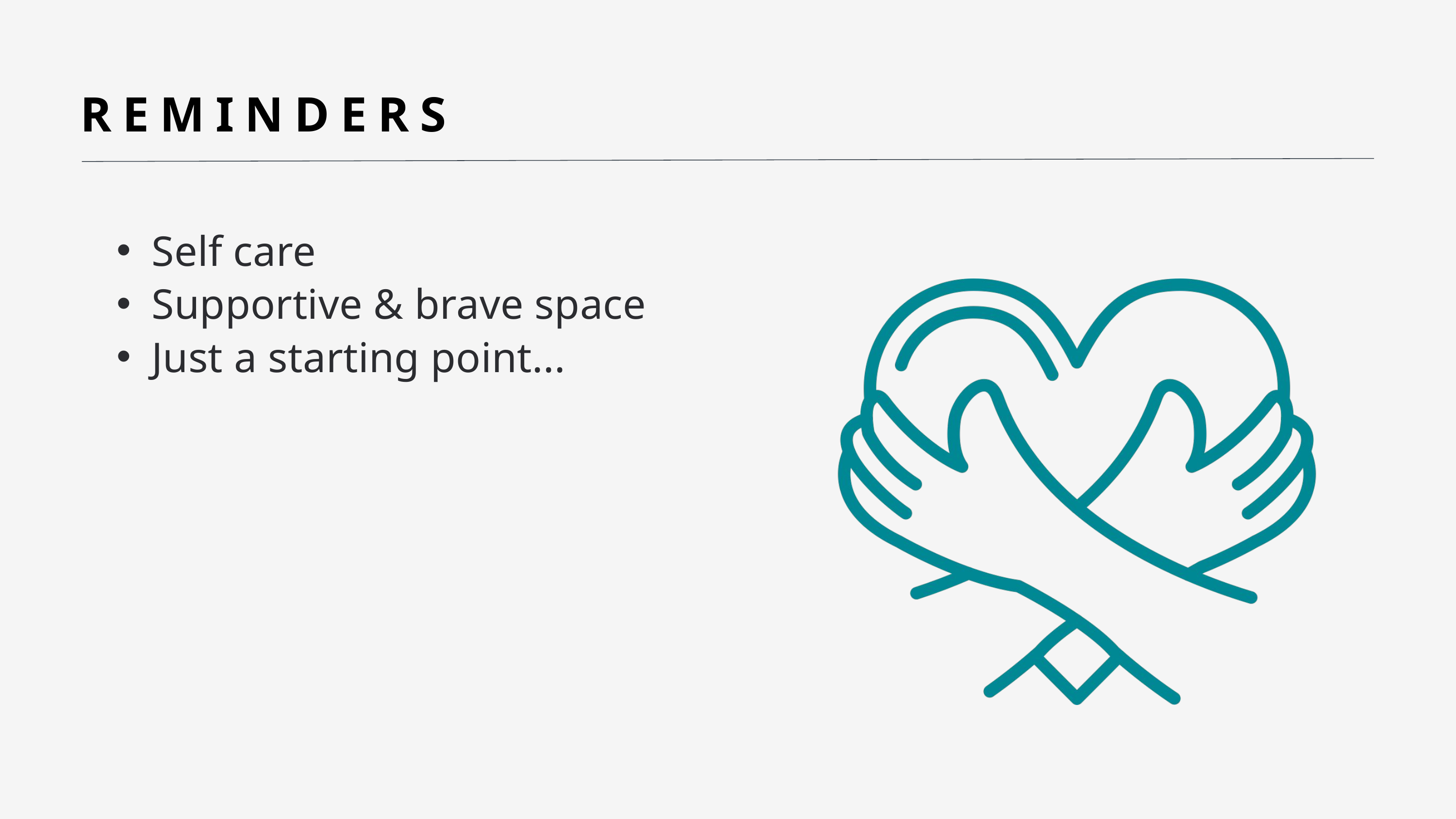

REMINDERS
Self care
Supportive & brave space
Just a starting point...

## Slide 4
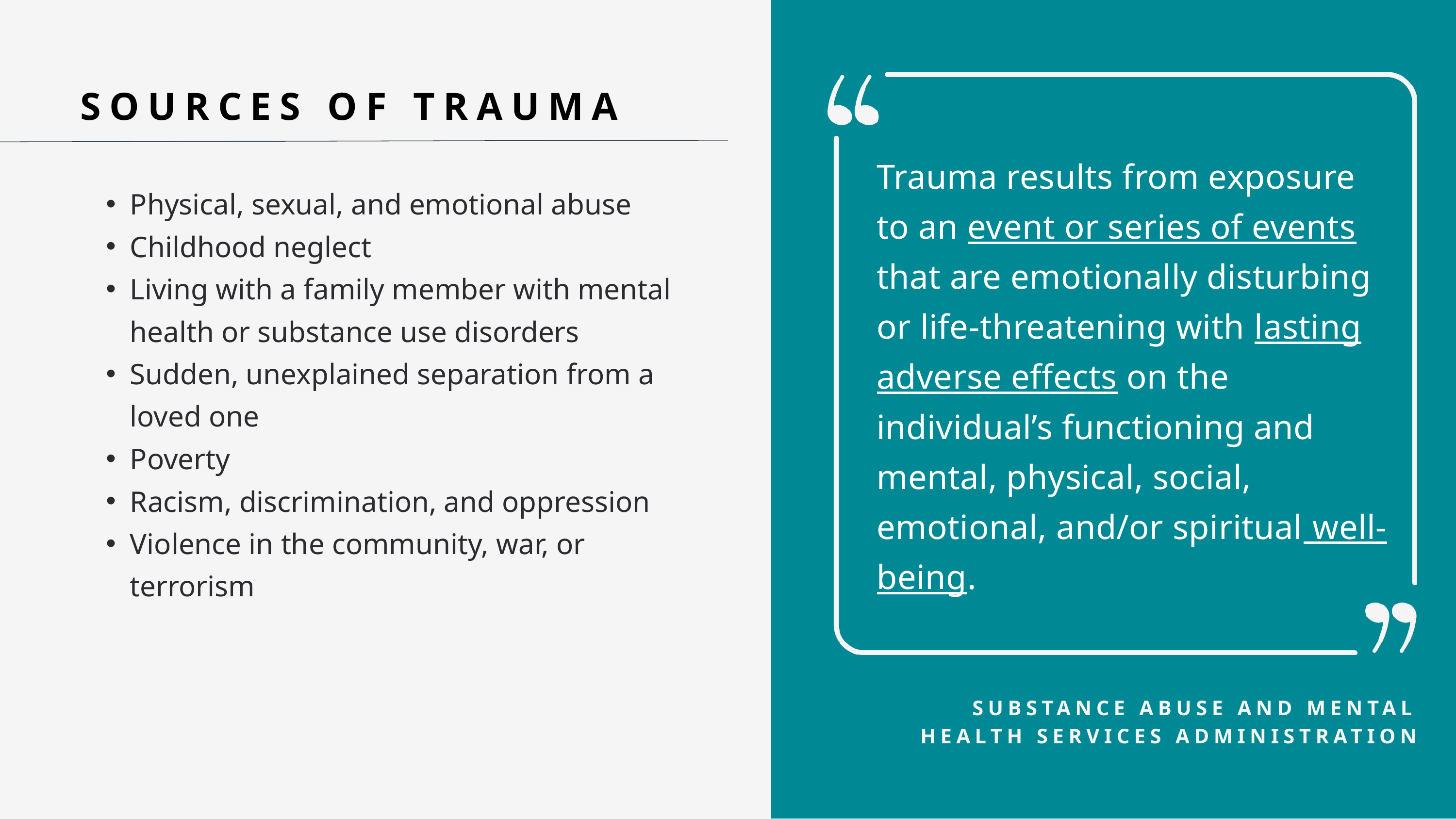

SOURCES OF TRAUMA
Trauma results from exposure to an event or series of events that are emotionally disturbing or life-threatening with lasting adverse effects on the individual’s functioning and mental, physical, social, emotional, and/or spiritual well-being.
Physical, sexual, and emotional abuse​
Childhood neglect​
Living with a family member with mental health or substance use disorders​
Sudden, unexplained separation from a loved one​
Poverty​
Racism, discrimination, and oppression​
Violence in the community, war, or terrorism
SUBSTANCE ABUSE AND MENTAL HEALTH SERVICES ADMINISTRATION

## Slide 5
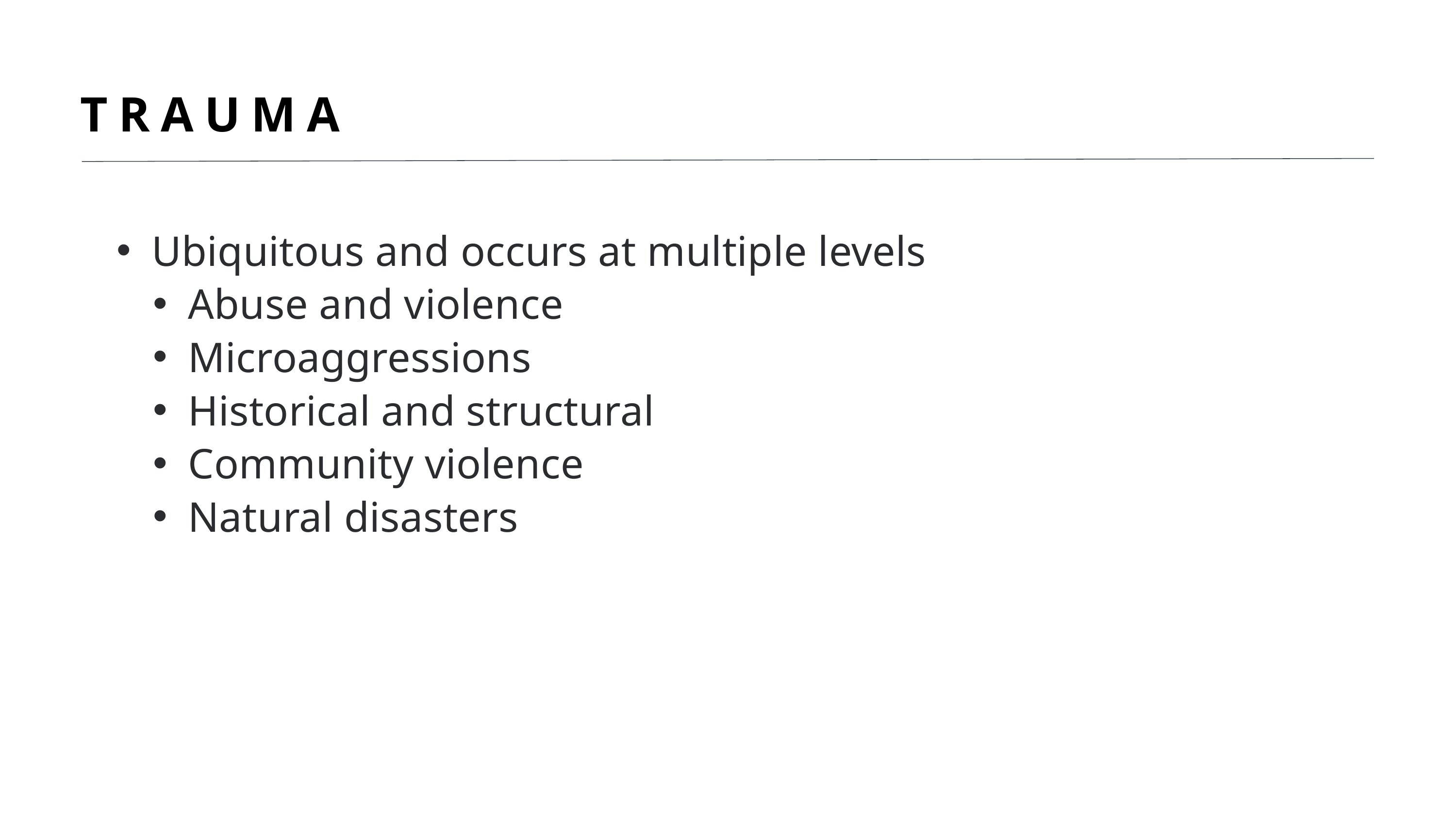

TRAUMA
Ubiquitous and occurs at multiple levels
Abuse and violence
Microaggressions
Historical and structural
Community violence
Natural disasters

## Slide 6
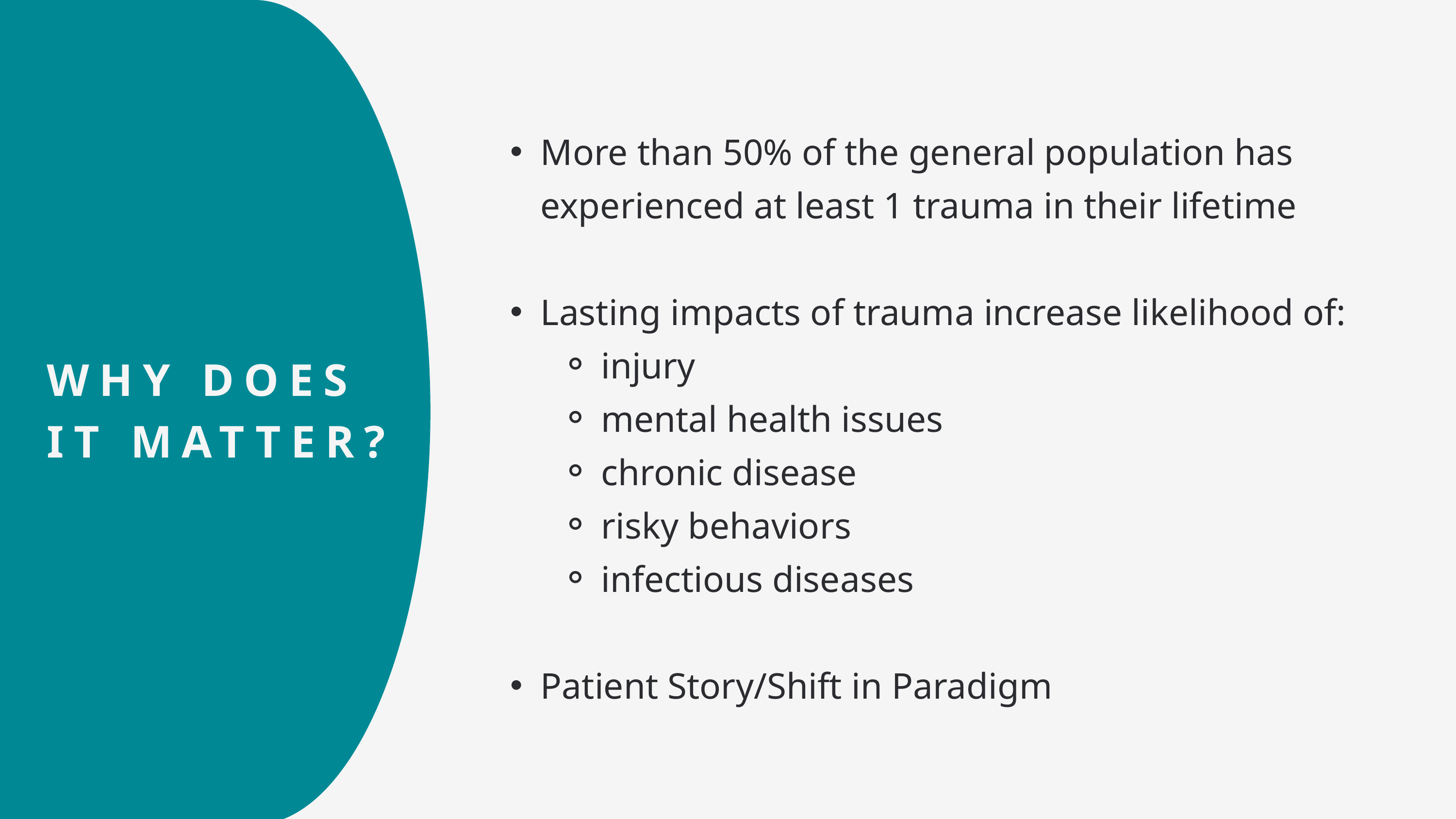

More than 50% of the general population has experienced at least 1 trauma in their lifetime
Lasting impacts of trauma increase likelihood of:
injury
mental health issues
chronic disease
risky behaviors
infectious diseases
Patient Story/Shift in Paradigm
WHY DOES IT MATTER?

## Slide 7
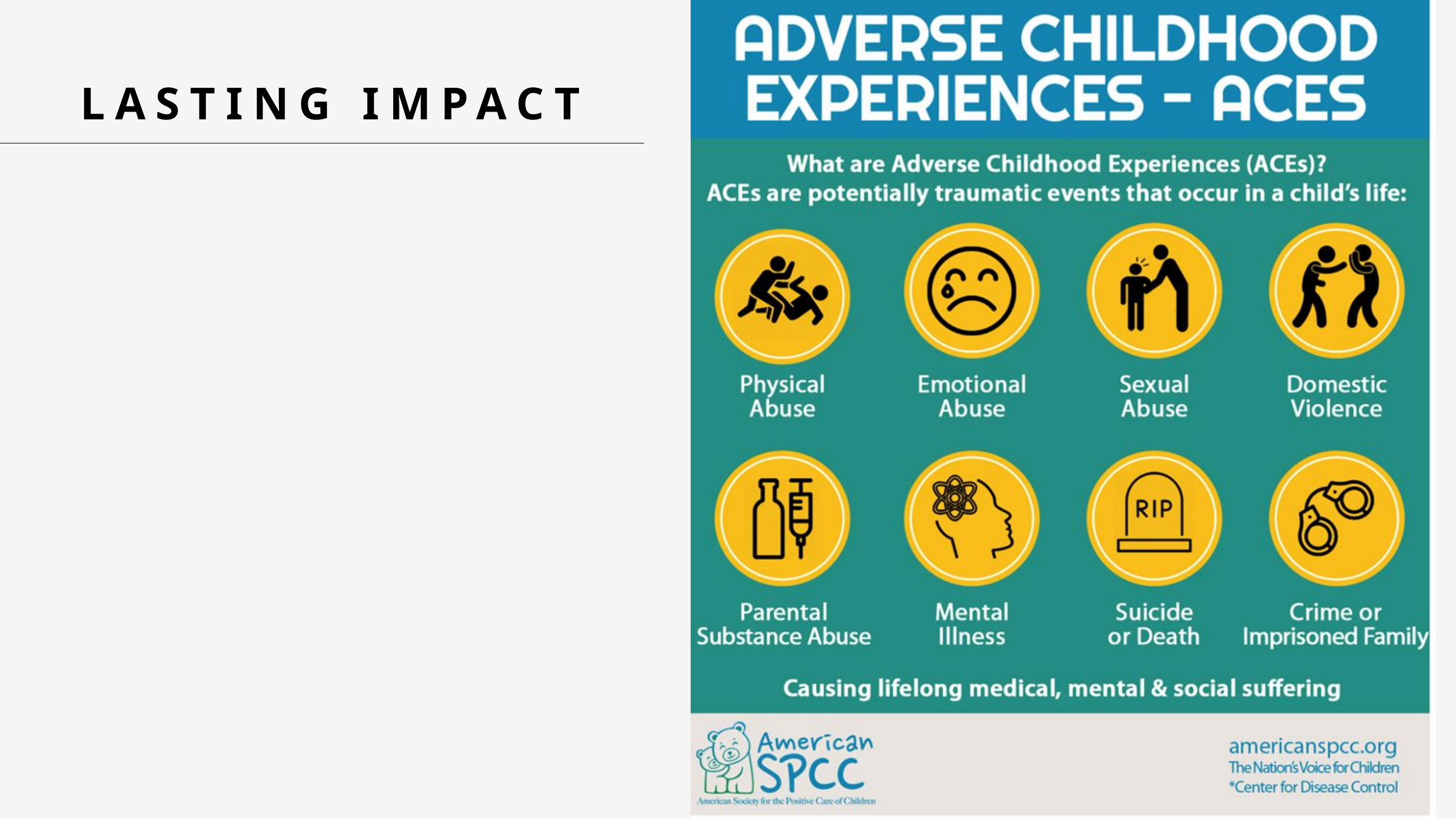

LASTING IMPACT

## Slide 8
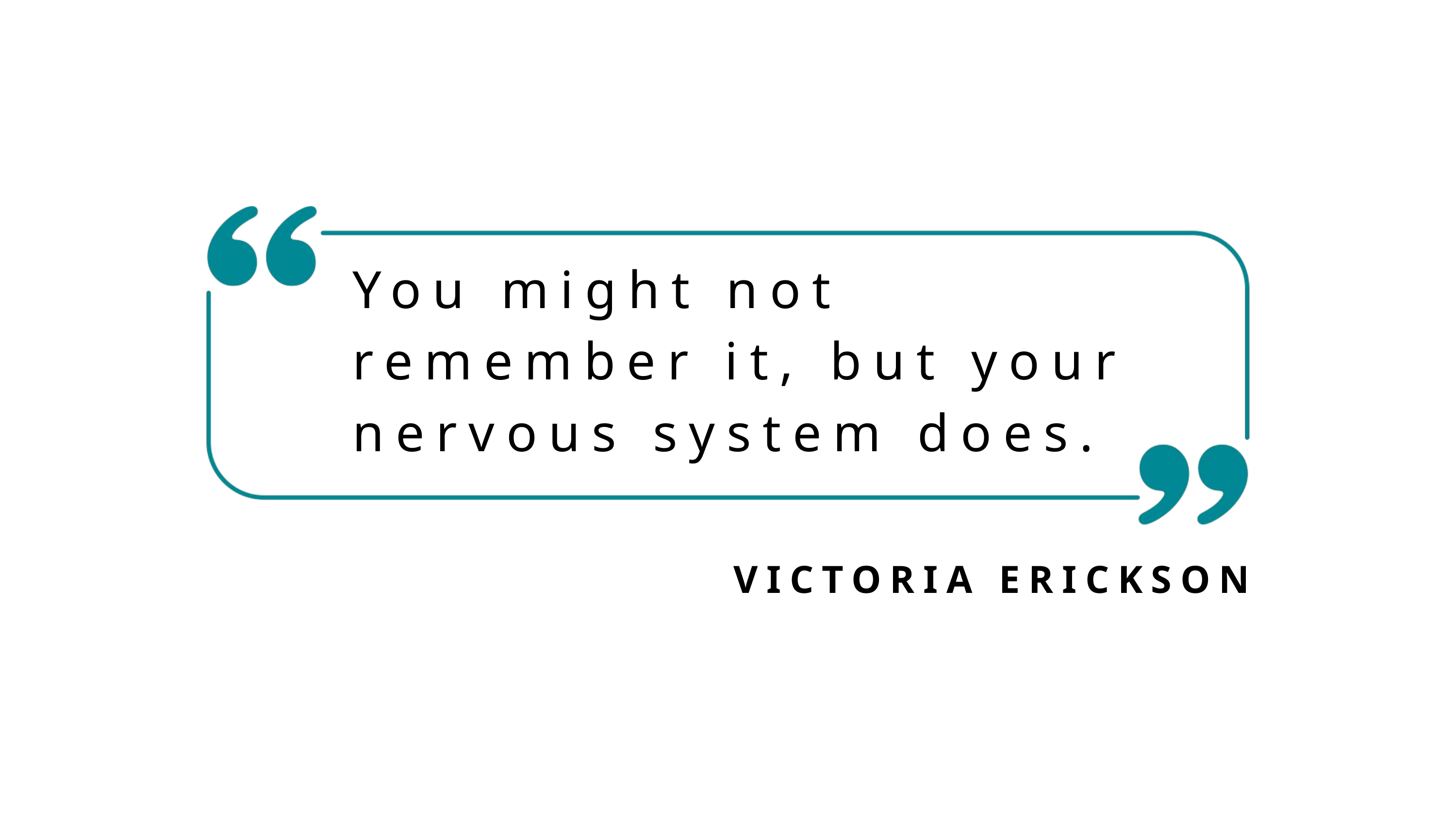

You might not remember it, but your nervous system does.
VICTORIA ERICKSON

## Slide 9
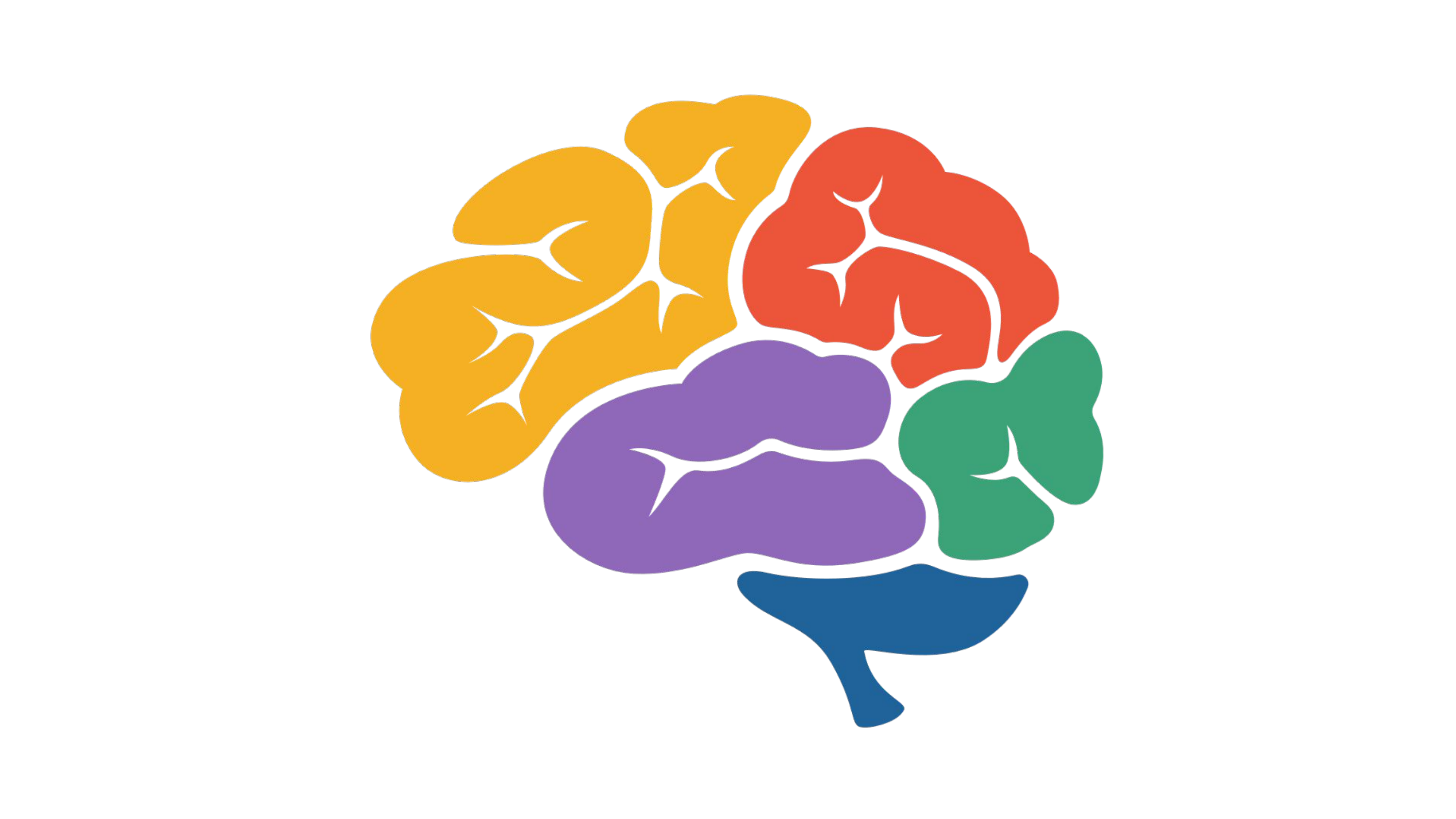

## Slide 10
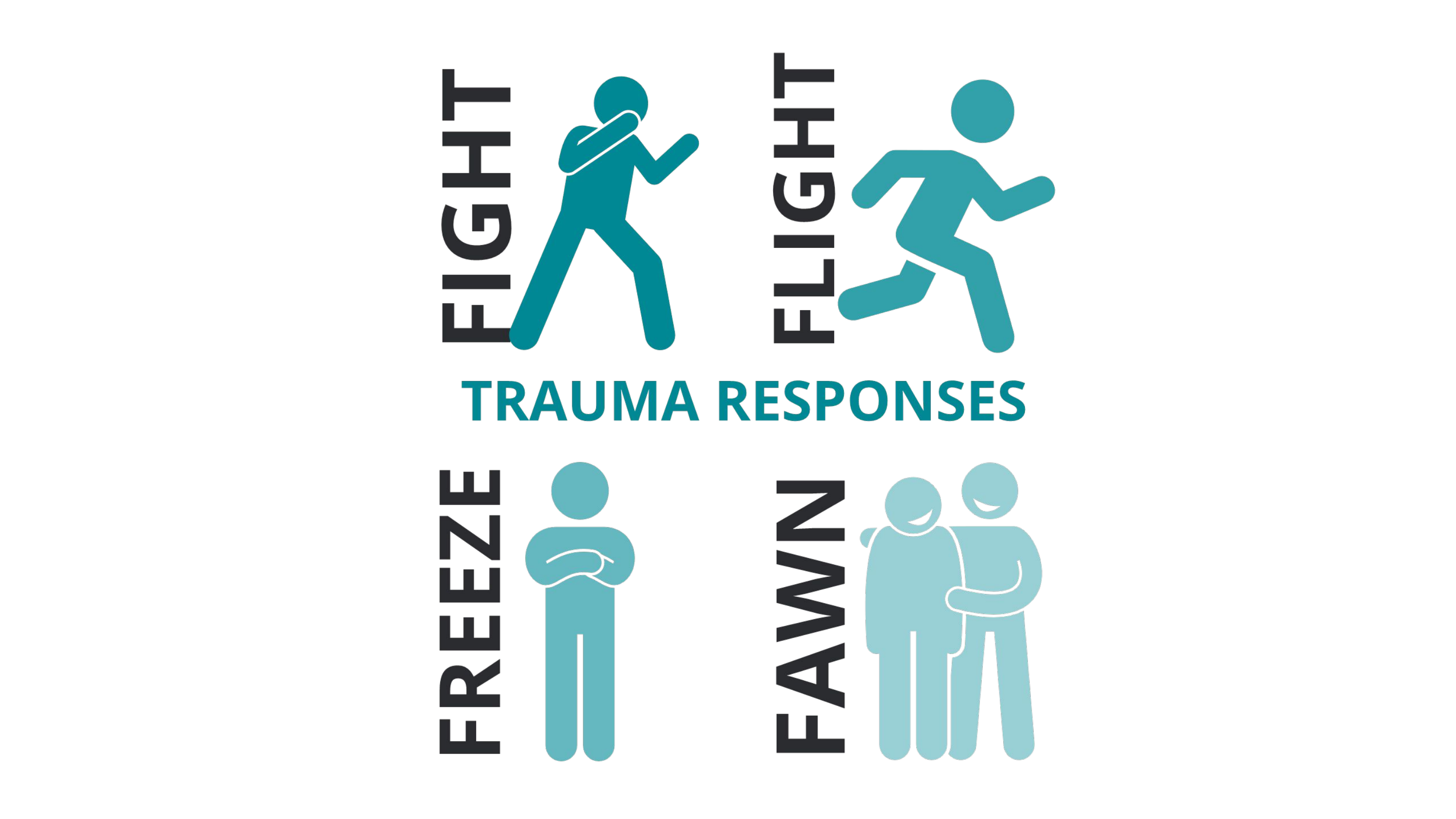

## Slide 11
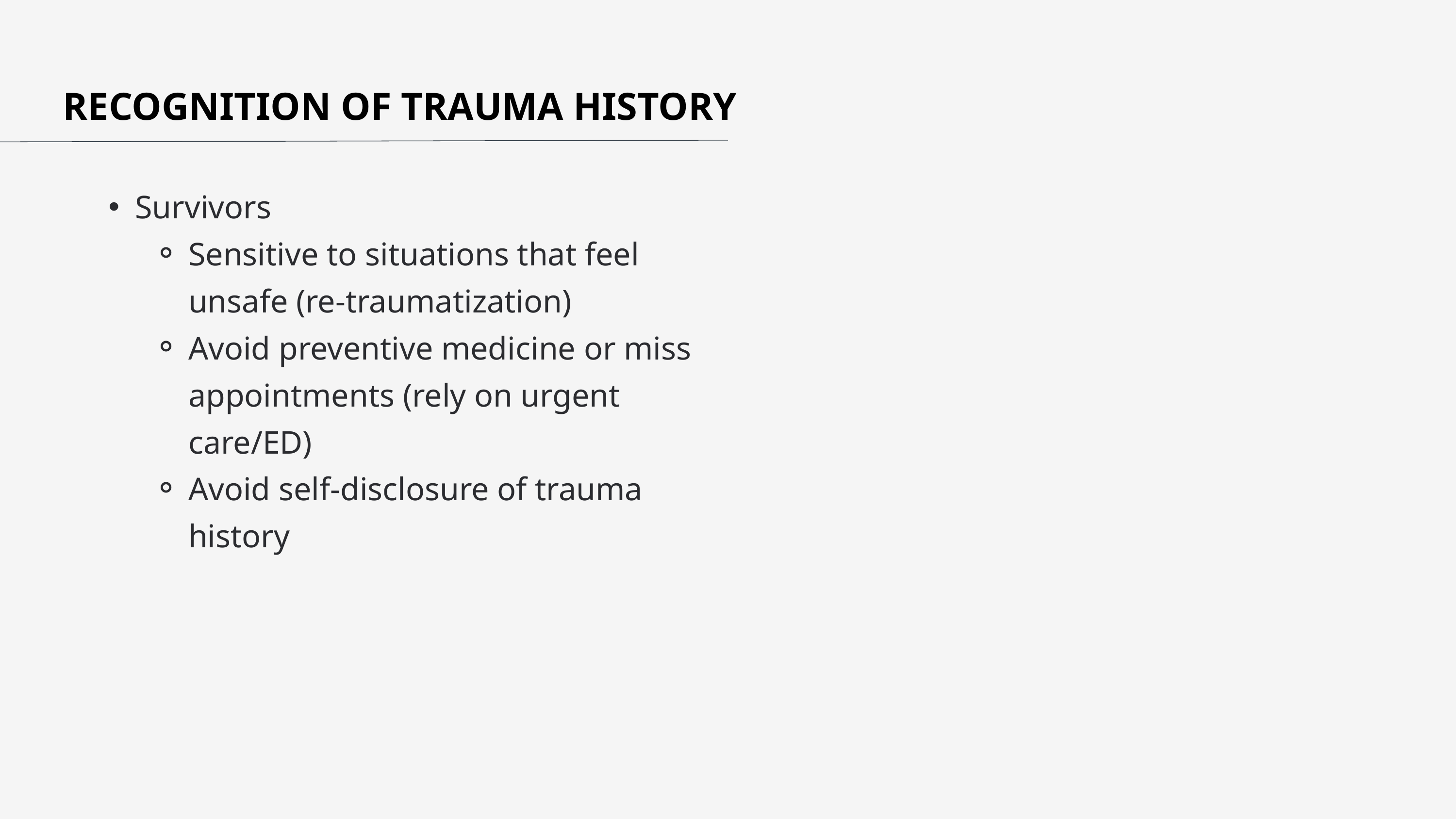

RECOGNITION OF TRAUMA HISTORY
Survivors
Sensitive to situations that feel unsafe (re-traumatization)
Avoid preventive medicine or miss appointments (rely on urgent care/ED)
Avoid self-disclosure of trauma history

## Slide 12
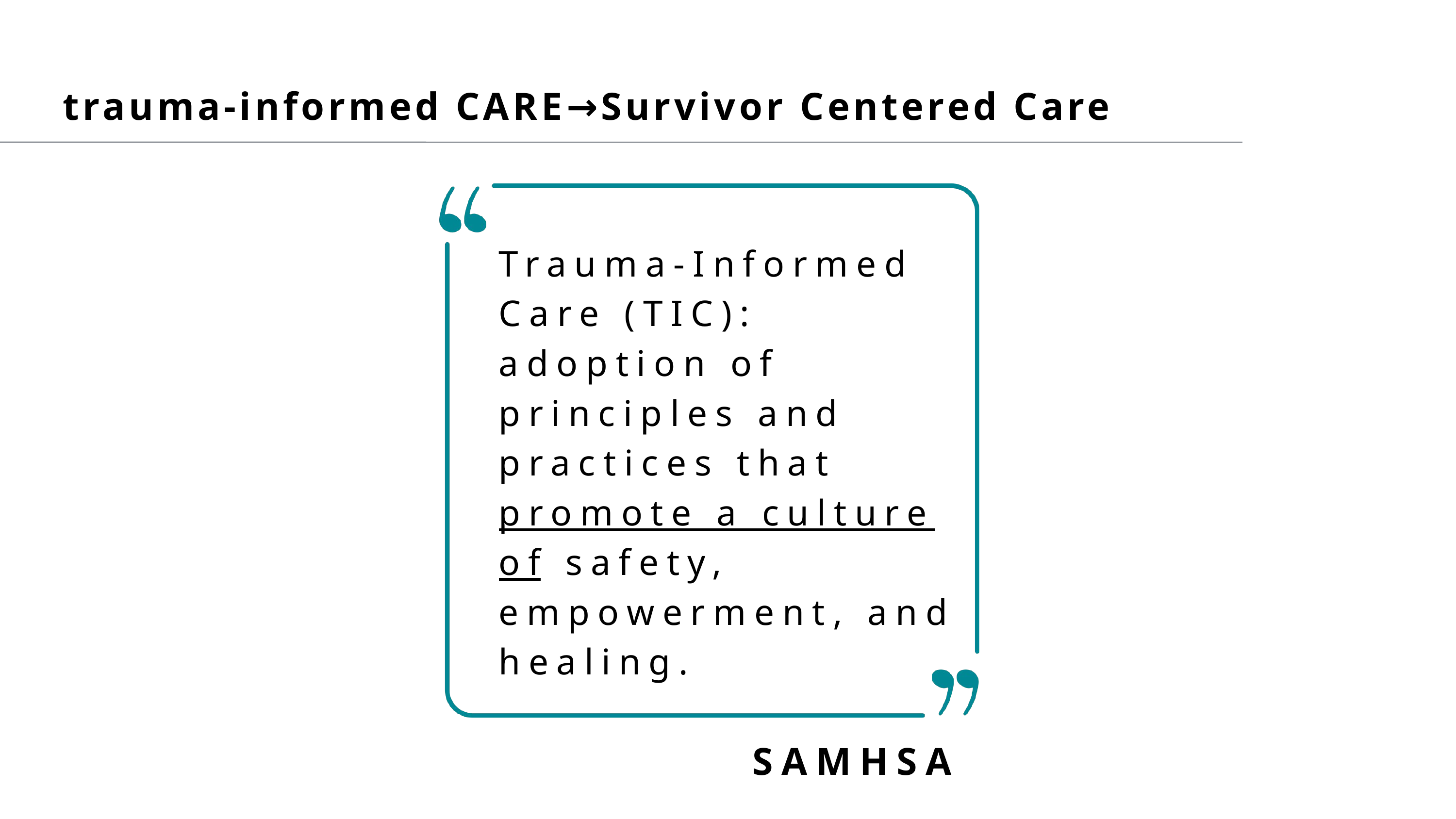

trauma-informed CARE→Survivor Centered Care
Trauma-Informed Care (TIC): adoption of principles and practices that promote a culture of safety, empowerment, and healing.
SAMHSA

## Slide 13
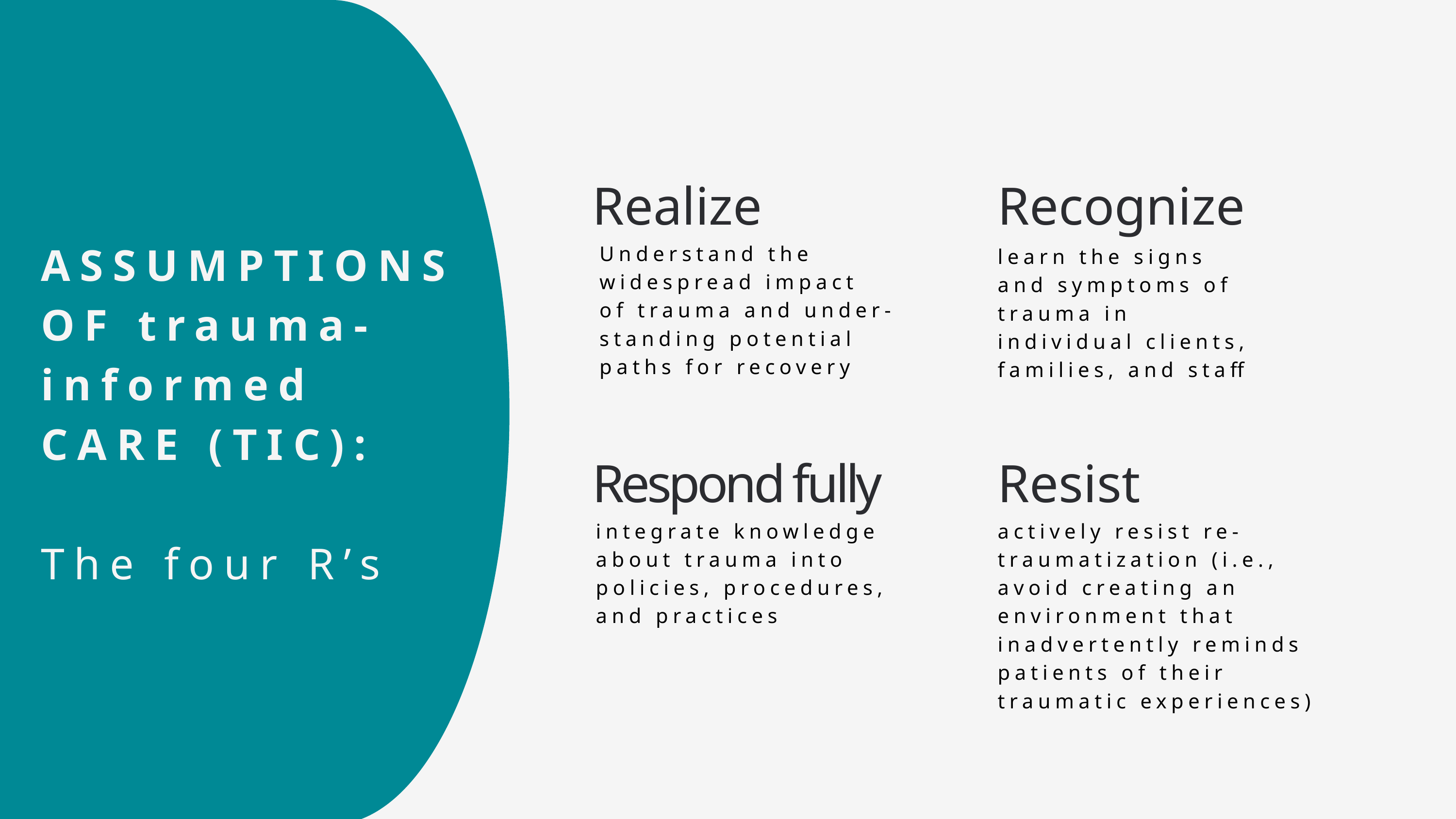

Realize
Recognize
ASSUMPTIONS OF trauma-informed CARE (TIC):
The four R’s
Understand the widespread impact of trauma and under-standing potential paths for recovery
learn the signs and symptoms of trauma in individual clients, families, and staff
Respond fully
Resist
integrate knowledge about trauma into policies, procedures, and practices
actively resist re-traumatization (i.e., avoid creating an environment that inadvertently reminds patients of their traumatic experiences)

## Slide 14
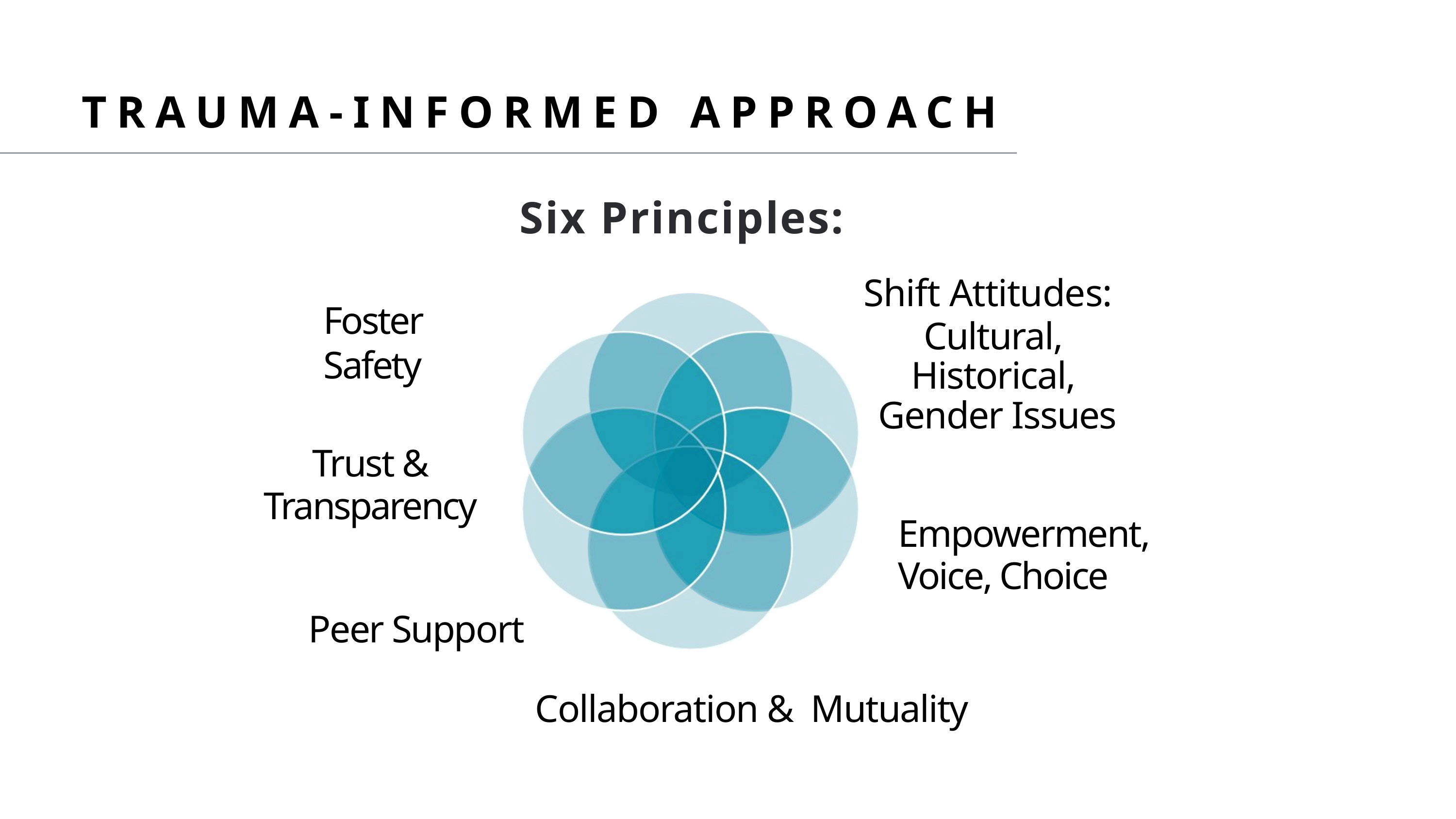

TRAUMA-INFORMED APPROACH
Six Principles:
Shift Attitudes:
Foster Safety
Cultural, Historical, Gender Issues
Trust &
Transparency
Empowerment,
Voice, Choice
Peer Support
Collaboration & Mutuality

## Slide 15
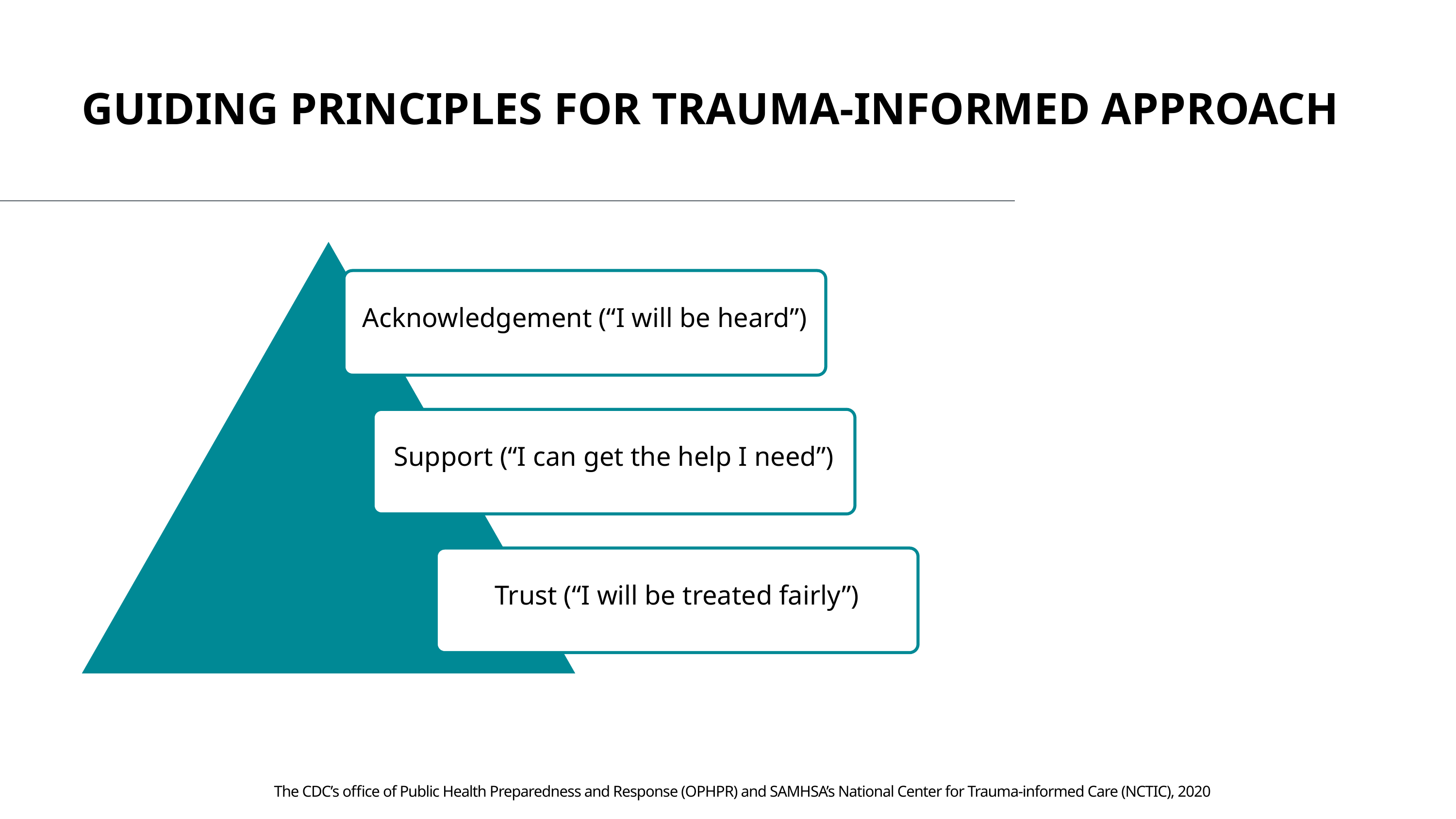

GUIDING PRINCIPLES FOR TRAUMA-INFORMED APPROACH
Acknowledgement (“I will be heard”)
Support (“I can get the help I need”)
Trust (“I will be treated fairly”)
The CDC’s office of Public Health Preparedness and Response (OPHPR) and SAMHSA’s National Center for Trauma-informed Care (NCTIC), 2020

## Slide 16
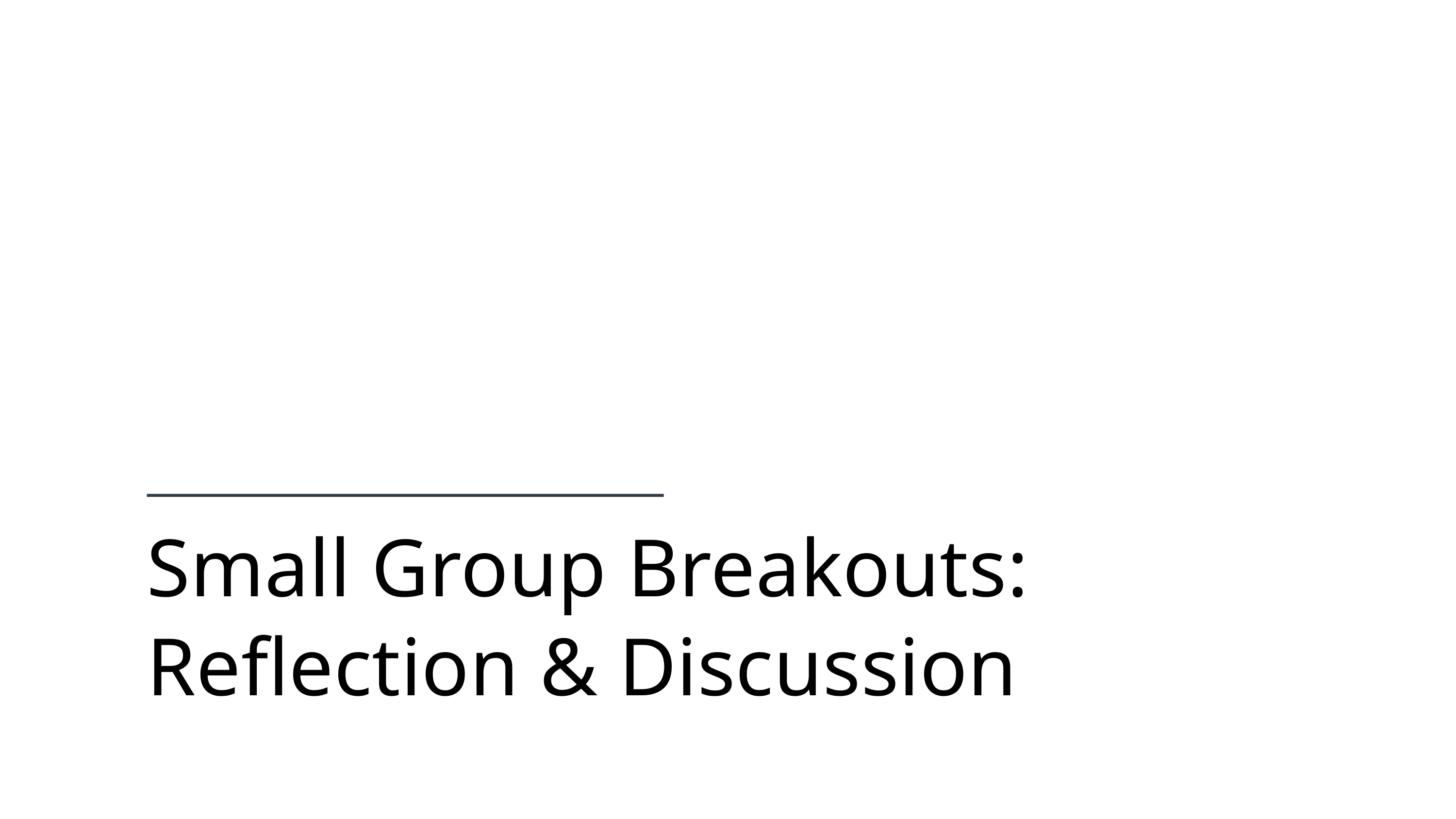

Small Group Breakouts:
Reflection & Discussion

## Slide 17
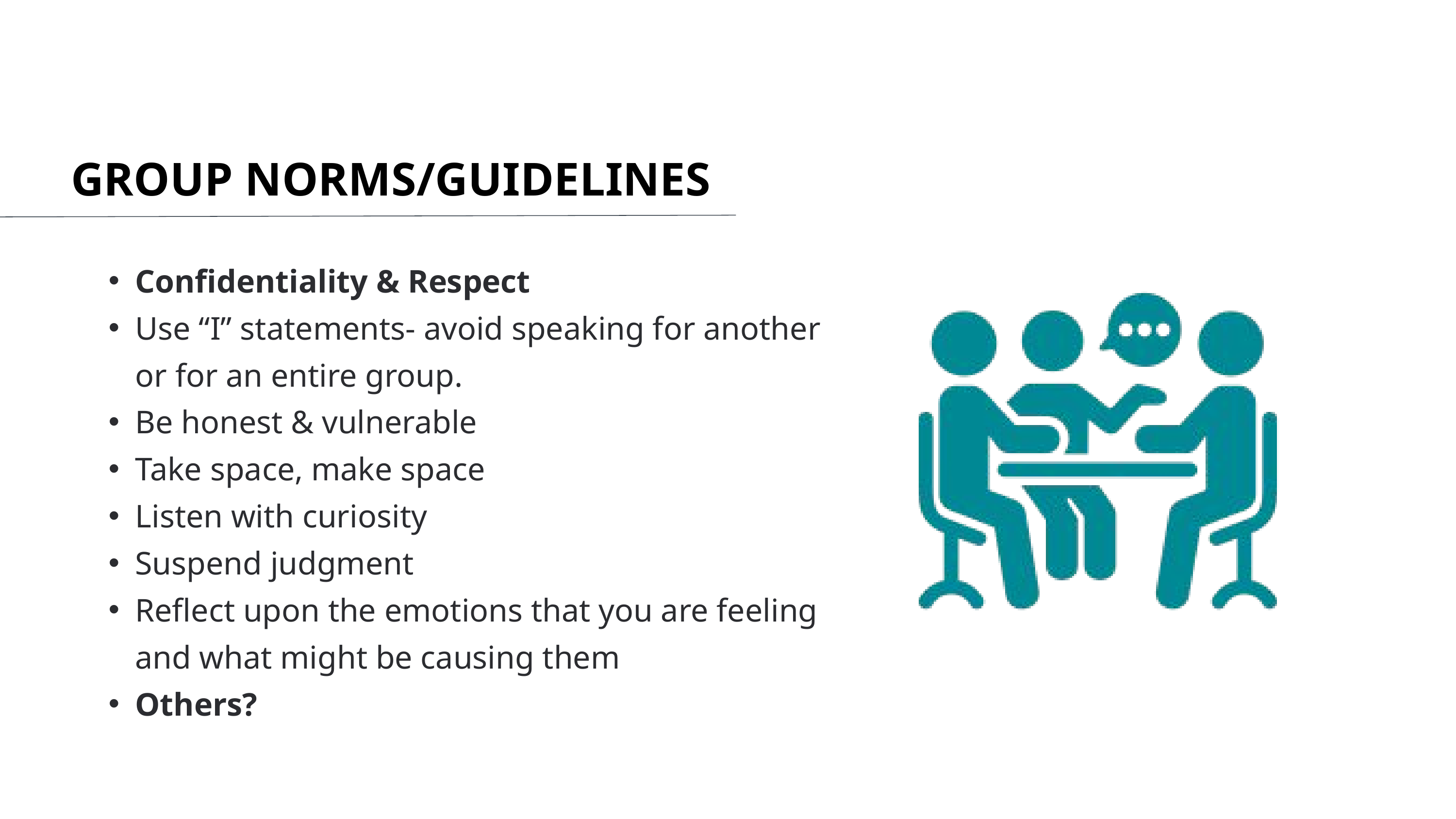

GROUP NORMS/GUIDELINES
Confidentiality & Respect
Use “I” statements- avoid speaking for another or for an entire group.
Be honest & vulnerable
Take space, make space
Listen with curiosity
Suspend judgment
Reflect upon the emotions that you are feeling and what might be causing them
Others?

## Slide 18
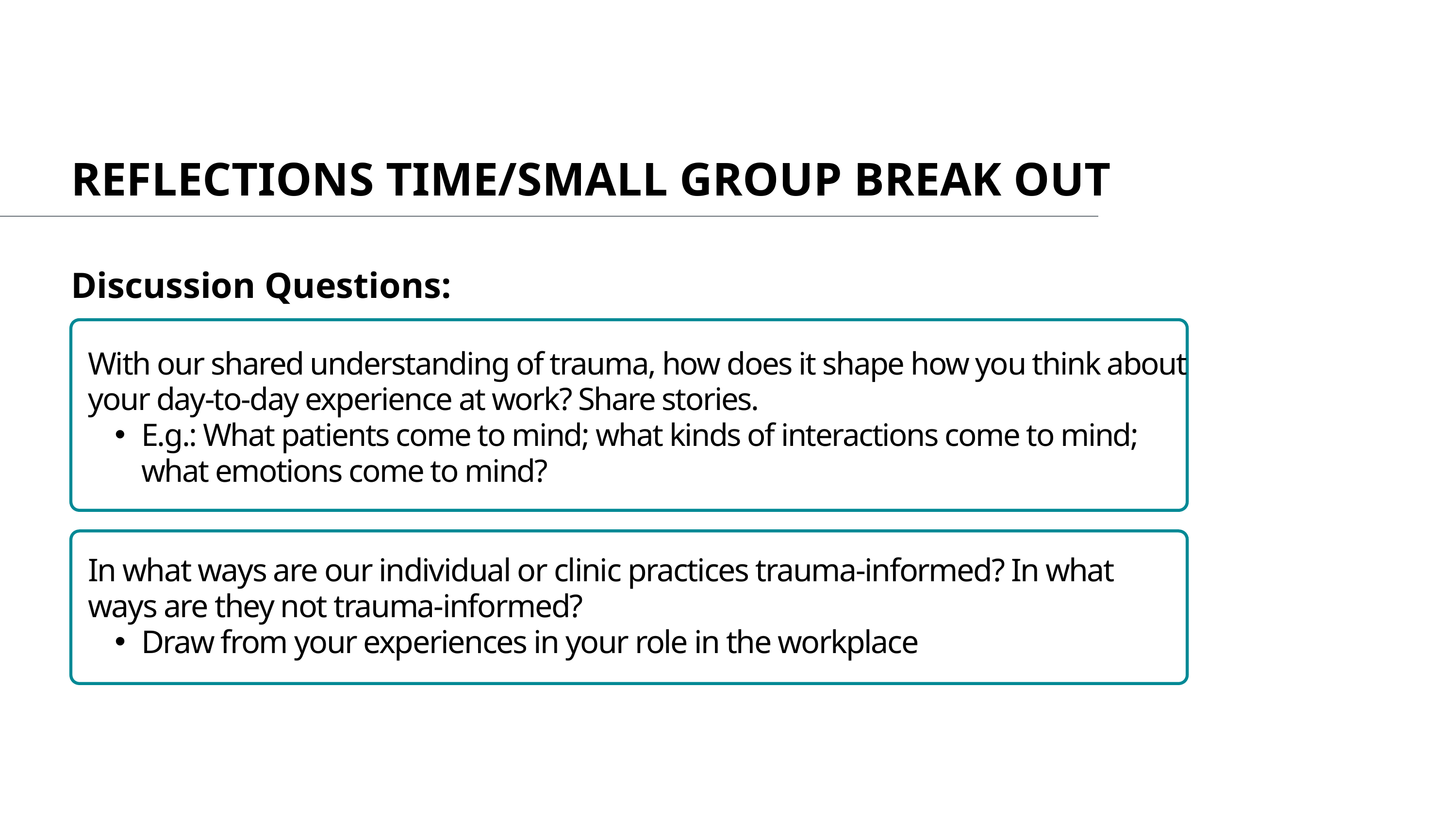

REFLECTIONS TIME/SMALL GROUP BREAK OUT
Discussion Questions:
With our shared understanding of trauma, how does it shape how you think about your day-to-day experience at work? Share stories.
E.g.: What patients come to mind; what kinds of interactions come to mind; what emotions come to mind?
In what ways are our individual or clinic practices trauma-informed? In what ways are they not trauma-informed?
Draw from your experiences in your role in the workplace

## Slide 19
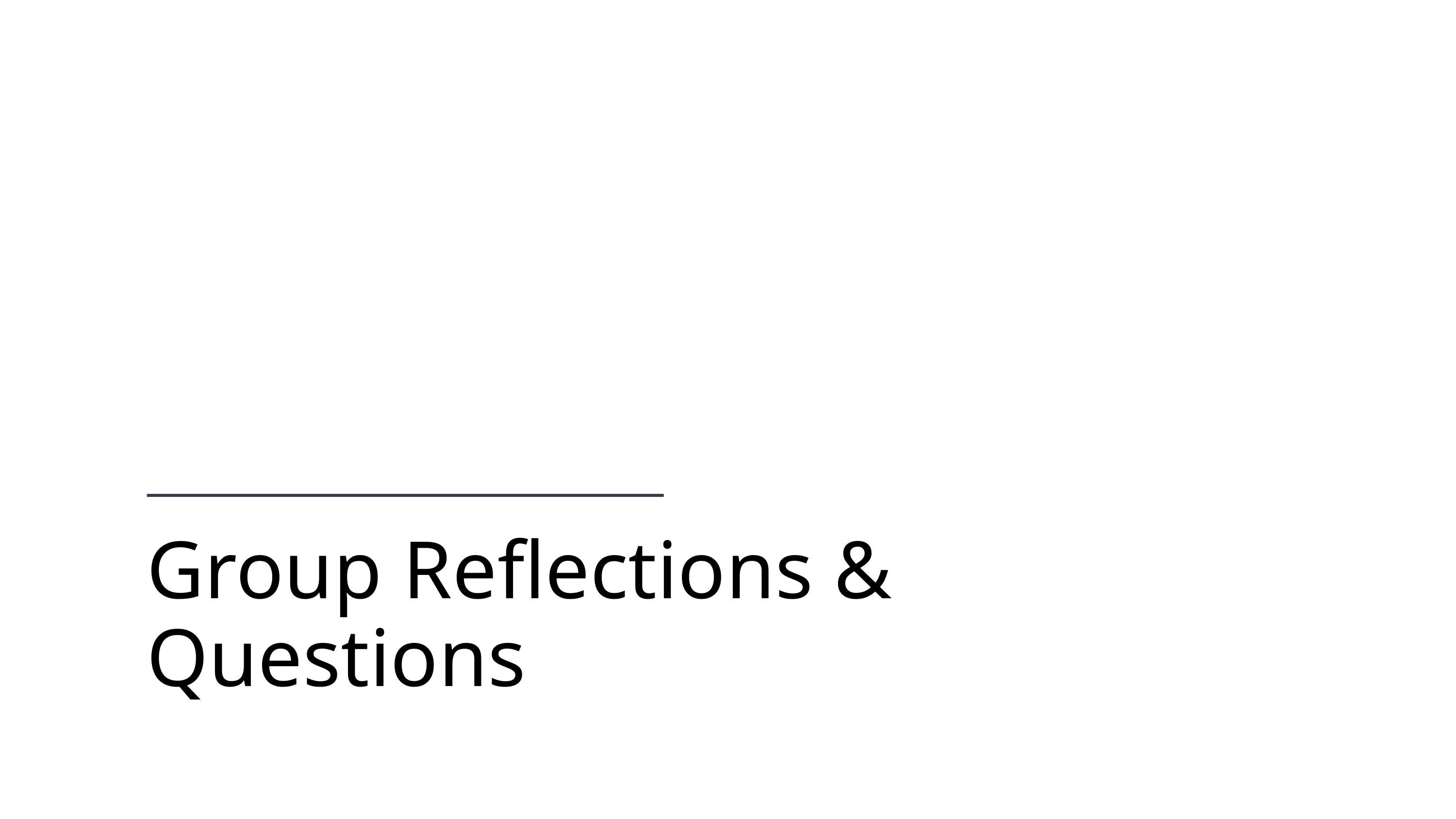

Group Reflections & Questions

## Slide 20
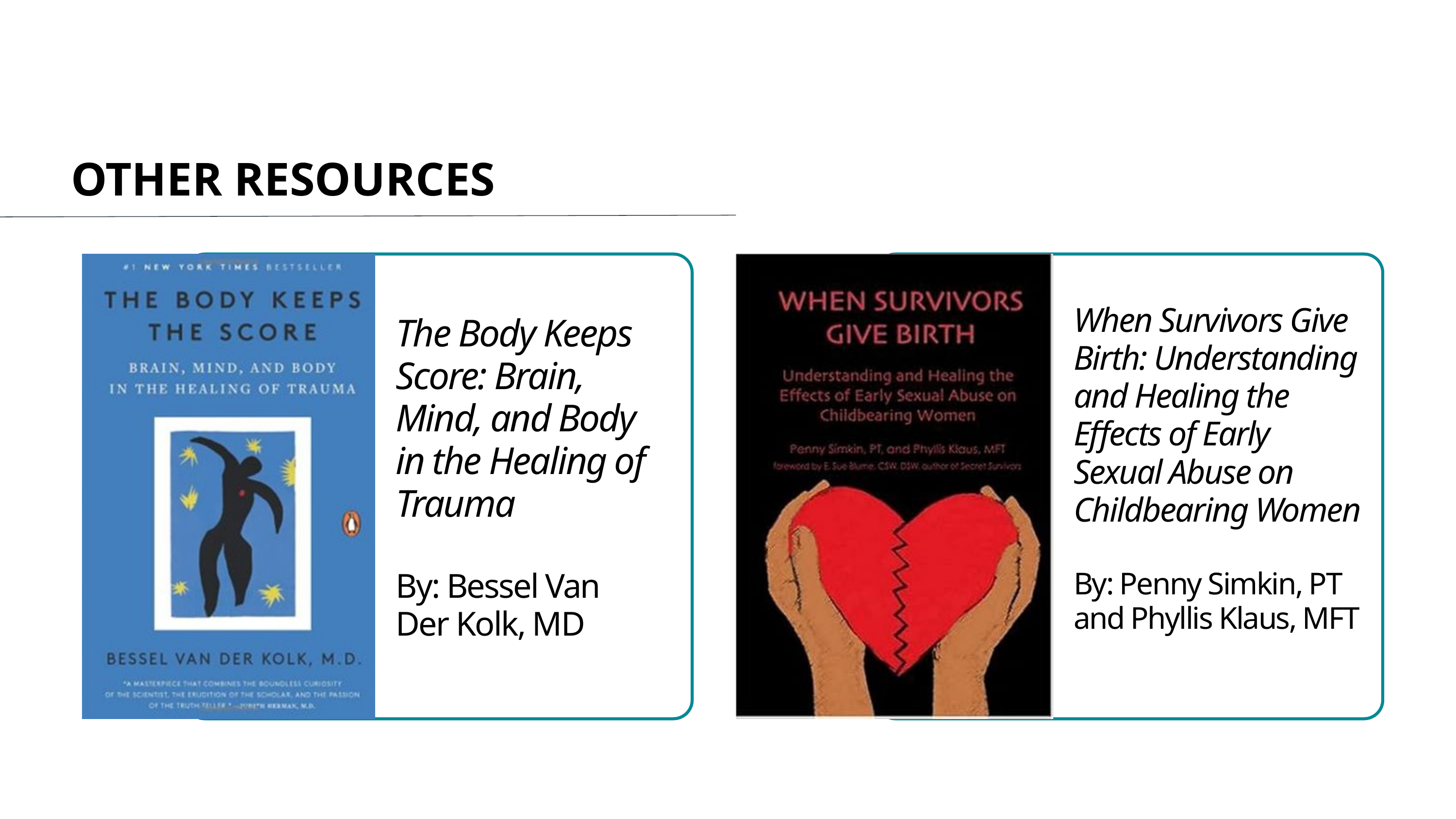

OTHER RESOURCES
When Survivors Give Birth: Understanding and Healing the Effects of Early Sexual Abuse on Childbearing Women
By: Penny Simkin, PT and Phyllis Klaus, MFT
The Body Keeps Score: Brain, Mind, and Body in the Healing of Trauma
By: Bessel Van Der Kolk, MD

## Slide 21
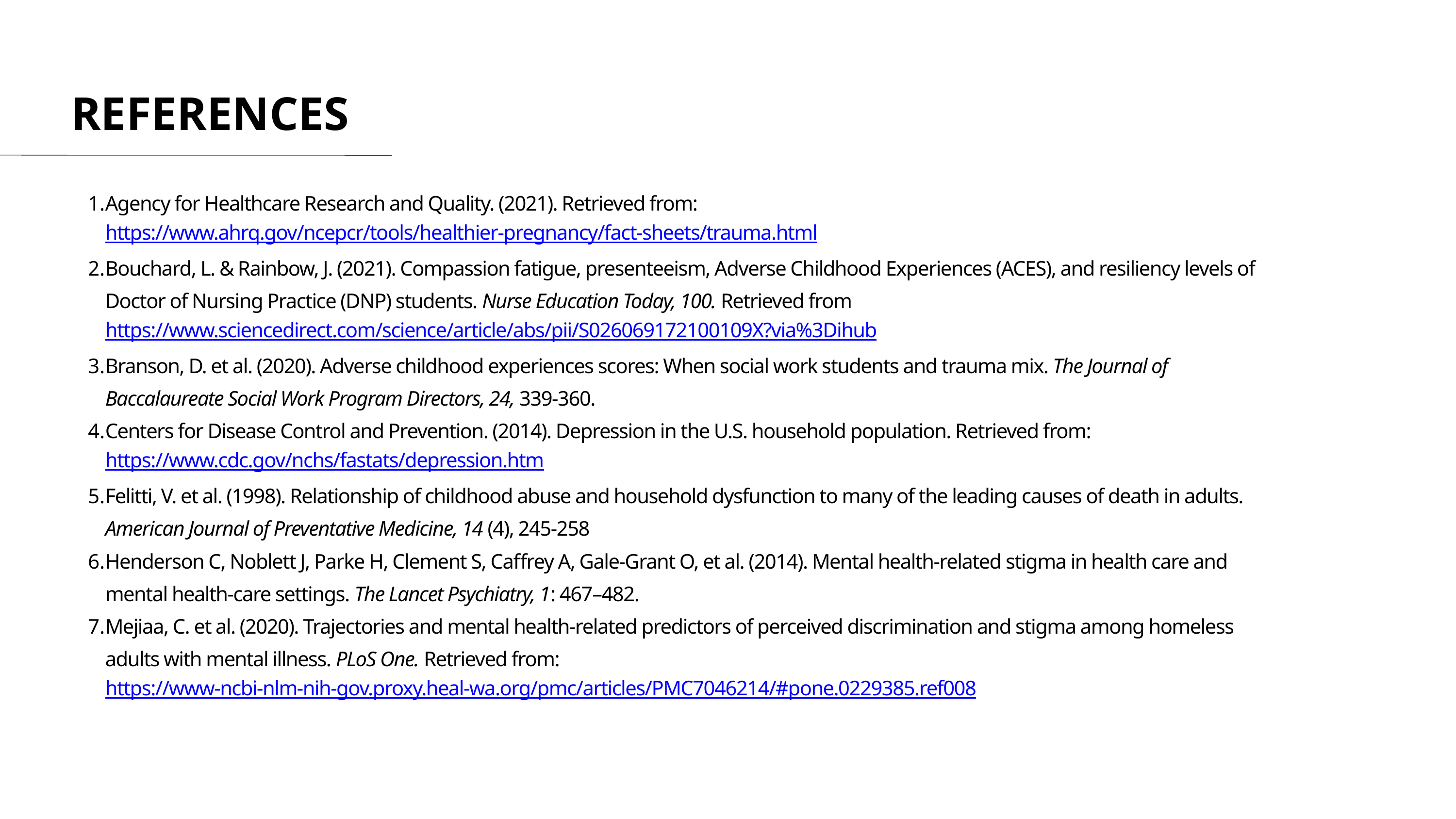

REFERENCES
Agency for Healthcare Research and Quality. (2021). Retrieved from: https://www.ahrq.gov/ncepcr/tools/healthier-pregnancy/fact-sheets/trauma.html
Bouchard, L. & Rainbow, J. (2021). Compassion fatigue, presenteeism, Adverse Childhood Experiences (ACES), and resiliency levels of Doctor of Nursing Practice (DNP) students. Nurse Education Today, 100. Retrieved from https://www.sciencedirect.com/science/article/abs/pii/S026069172100109X?via%3Dihub
Branson, D. et al. (2020). Adverse childhood experiences scores: When social work students and trauma mix. The Journal of Baccalaureate Social Work Program Directors, 24, 339-360.
Centers for Disease Control and Prevention. (2014). Depression in the U.S. household population. Retrieved from: https://www.cdc.gov/nchs/fastats/depression.htm
Felitti, V. et al. (1998). Relationship of childhood abuse and household dysfunction to many of the leading causes of death in adults. American Journal of Preventative Medicine, 14 (4), 245-258
Henderson C, Noblett J, Parke H, Clement S, Caffrey A, Gale-Grant O, et al. (2014). Mental health-related stigma in health care and mental health-care settings. The Lancet Psychiatry, 1: 467–482.
Mejiaa, C. et al. (2020). Trajectories and mental health-related predictors of perceived discrimination and stigma among homeless adults with mental illness. PLoS One. Retrieved from: https://www-ncbi-nlm-nih-gov.proxy.heal-wa.org/pmc/articles/PMC7046214/#pone.0229385.ref008
