# Supplementary material for: A Trauma-informed Care Curriculum for Perinatal Providers, Staff, and Learners
Source: MedEdPORTAL. 2025 Dec 9;21:11563. doi: 10.15766/mep_2374-8265.11563 (PMC12686155; doi:10.15766/mep_2374-8265.11563)
Supplement: Supplementary file 1 — Part 1 - Overview of TIC.pptxPart 2 - TIC in Perinatal Care.pptxPart 3 - Vicarious Trauma.pptxPart 4 - Community Voices & Reflection.pptxPresurvey.docxPostsurvey.pdf [file mep_2374-8265.11563-s001.zip › B. Part 2 - TIC in Perinatal Care.pptx]

## Slide 1
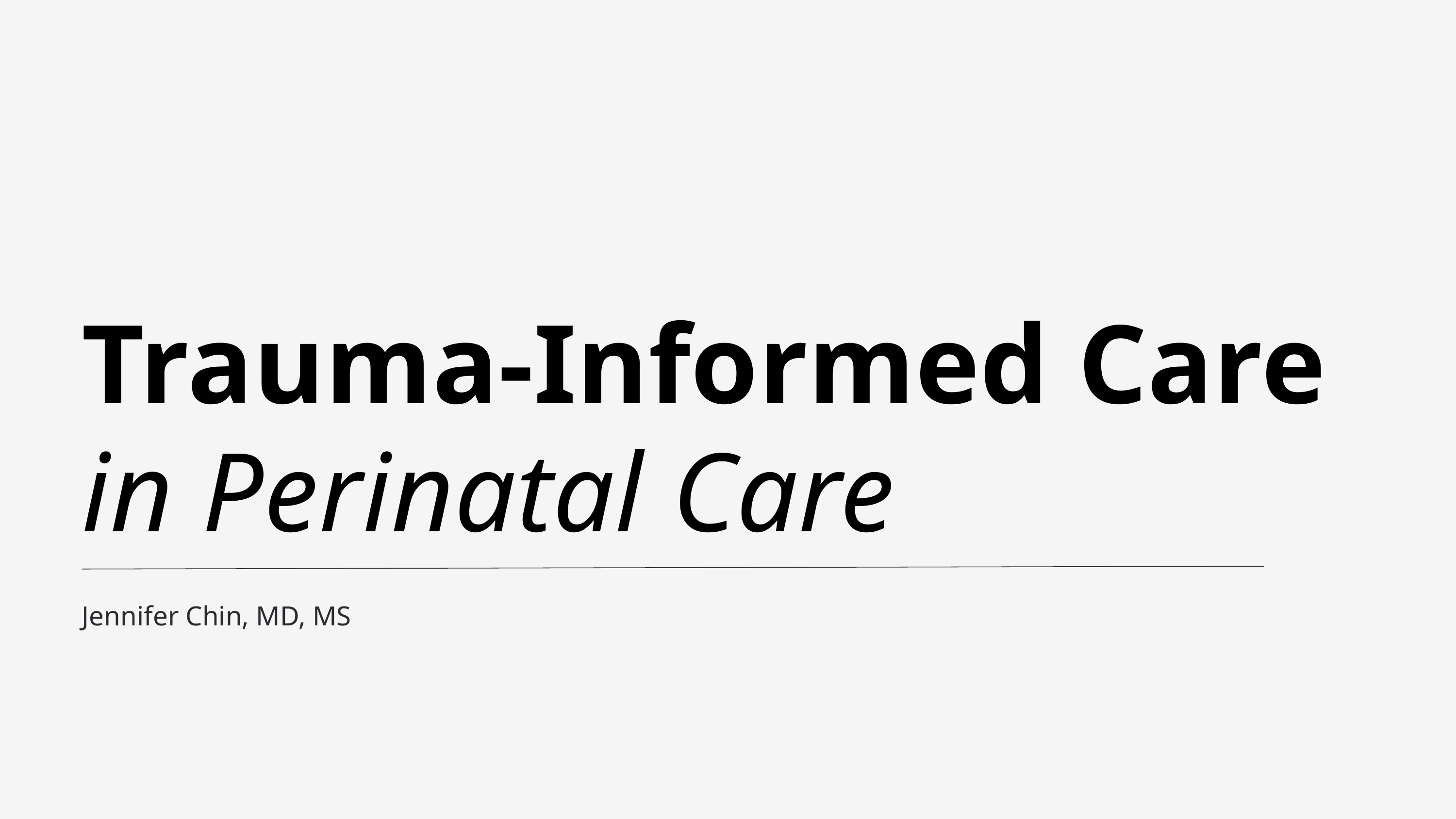

Trauma-Informed Care
in Perinatal Care
Jennifer Chin, MD, MS

## Slide 2
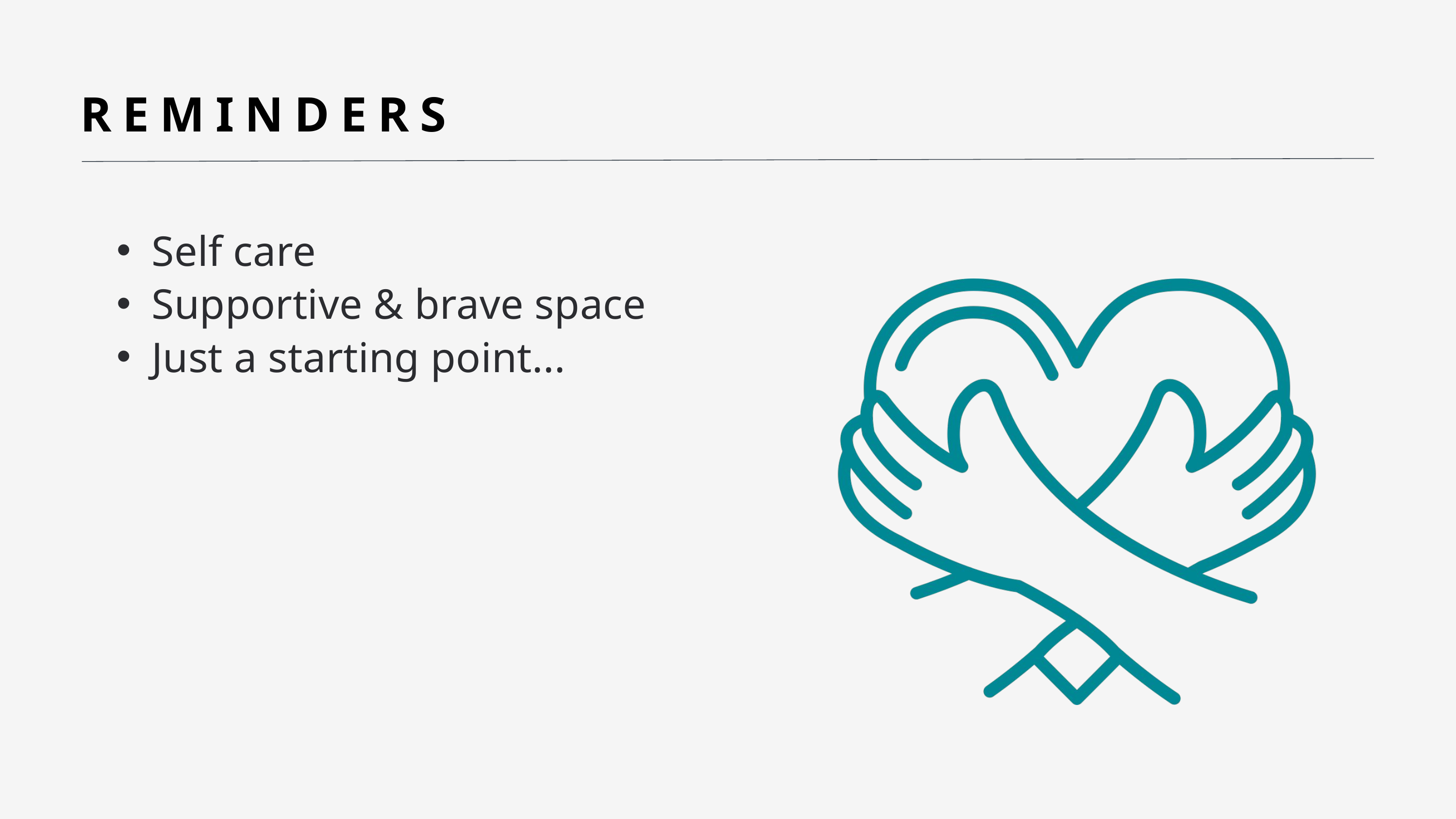

REMINDERS
Self care
Supportive & brave space
Just a starting point...

## Slide 3
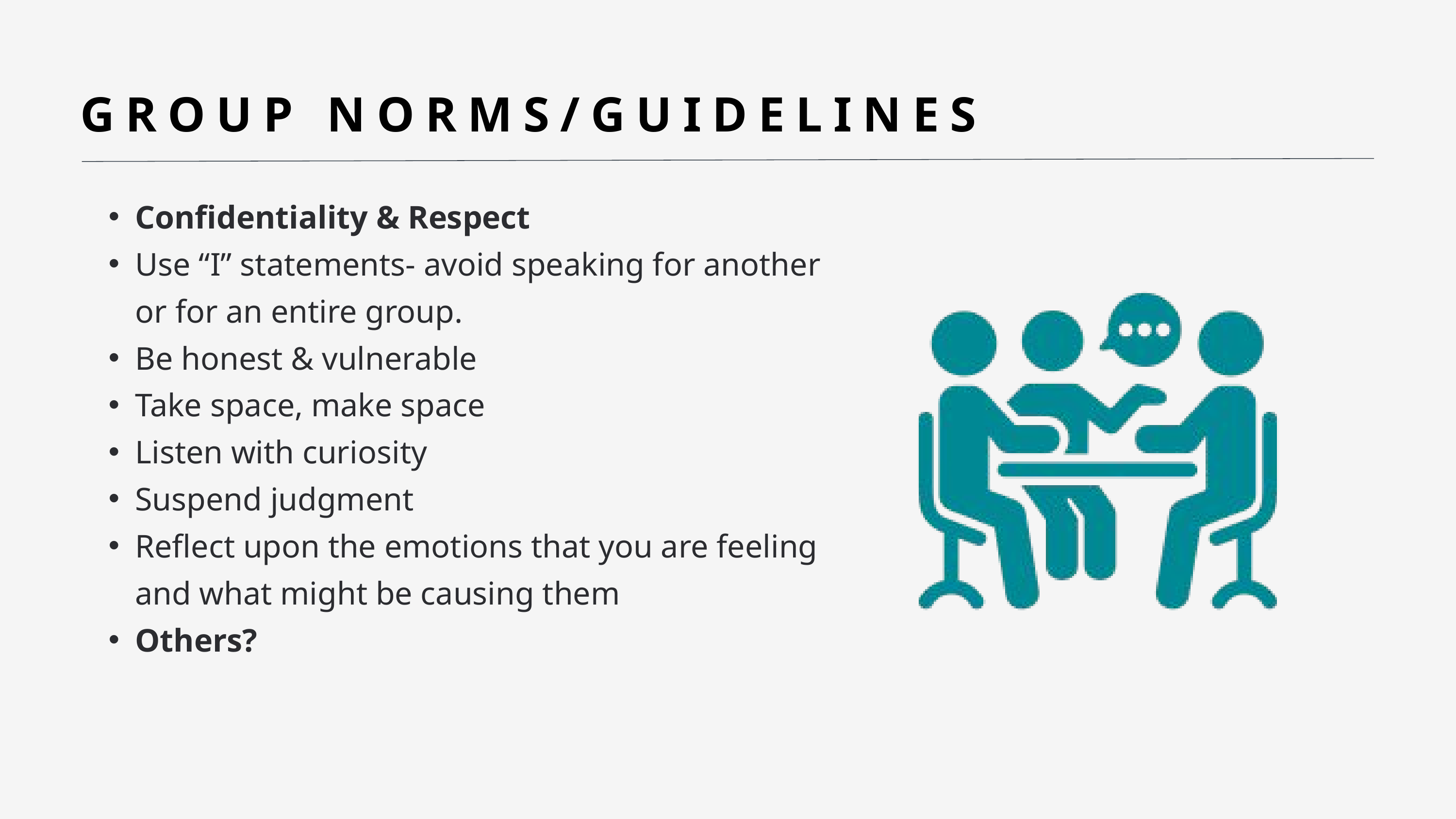

GROUP NORMS/GUIDELINES
Confidentiality & Respect
Use “I” statements- avoid speaking for another or for an entire group.
Be honest & vulnerable
Take space, make space
Listen with curiosity
Suspend judgment
Reflect upon the emotions that you are feeling and what might be causing them
Others?

## Slide 4
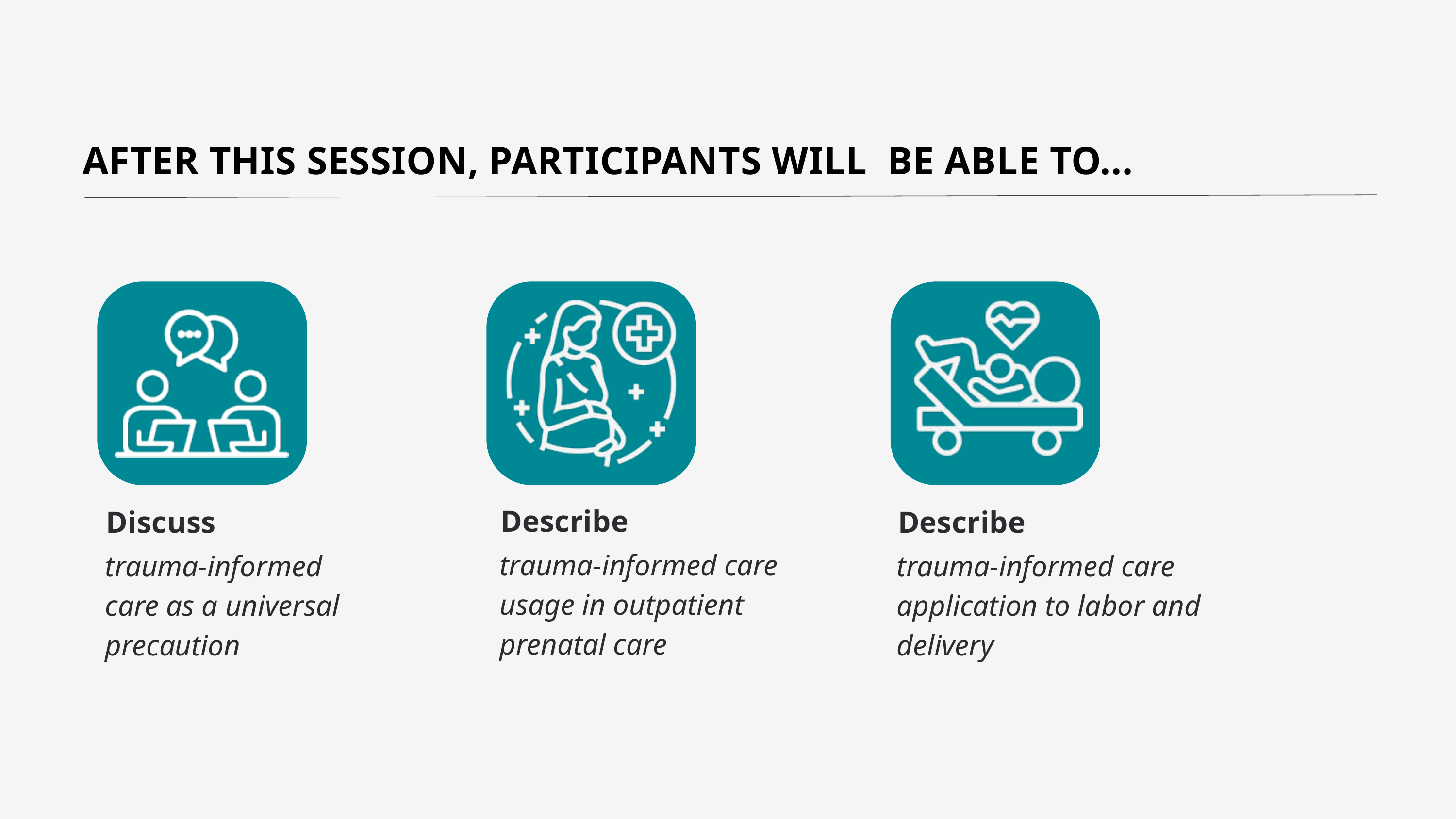

AFTER THIS SESSION, PARTICIPANTS WILL BE ABLE TO...
Describe
Discuss
Describe
trauma-informed care usage in outpatient prenatal care
trauma-informed care as a universal precaution
trauma-informed care application to labor and delivery

## Slide 5
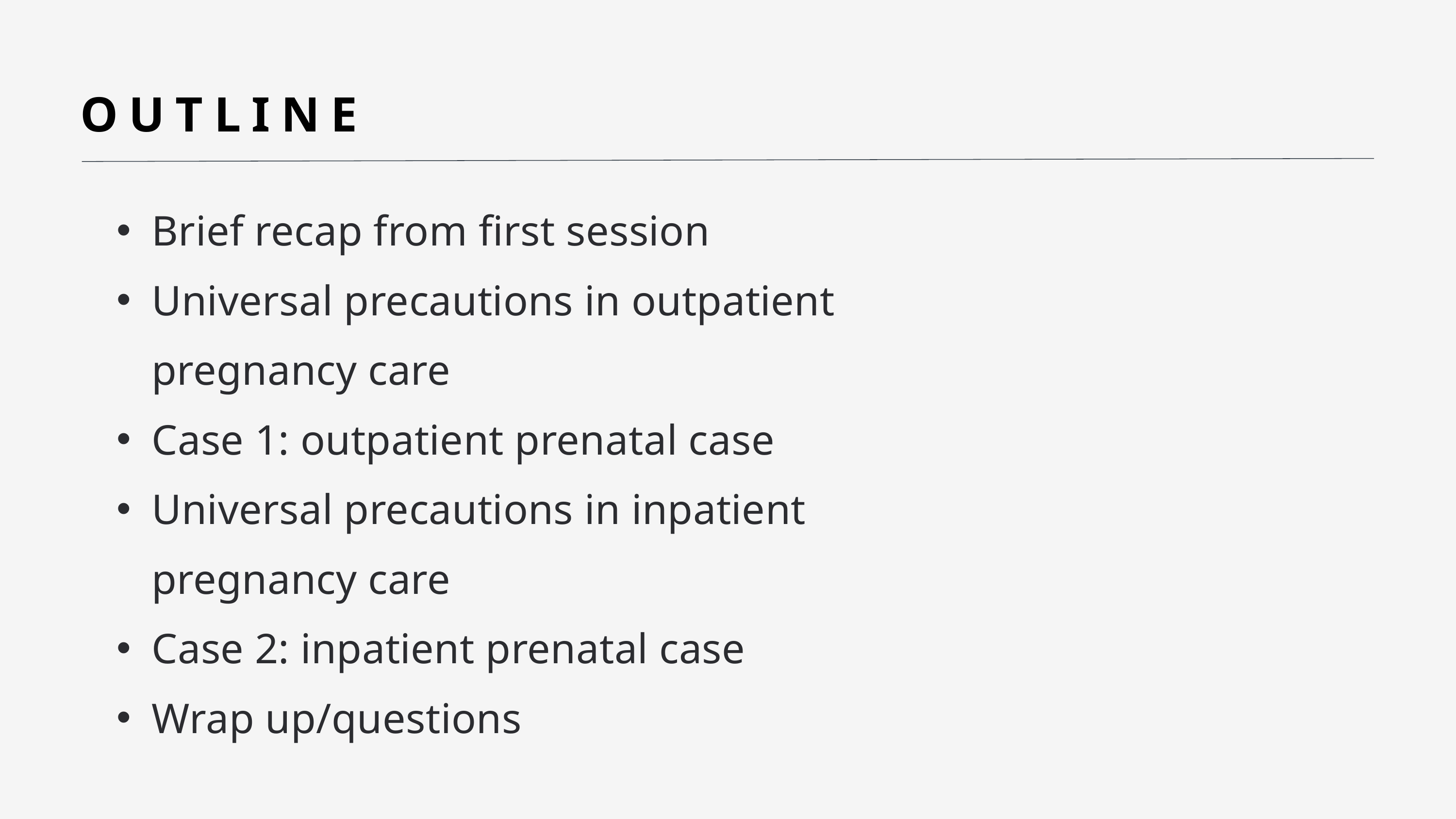

OUTLINE
Brief recap from first session
Universal precautions in outpatient pregnancy care
Case 1: outpatient prenatal case
Universal precautions in inpatient pregnancy care
Case 2: inpatient prenatal case
Wrap up/questions

## Slide 6
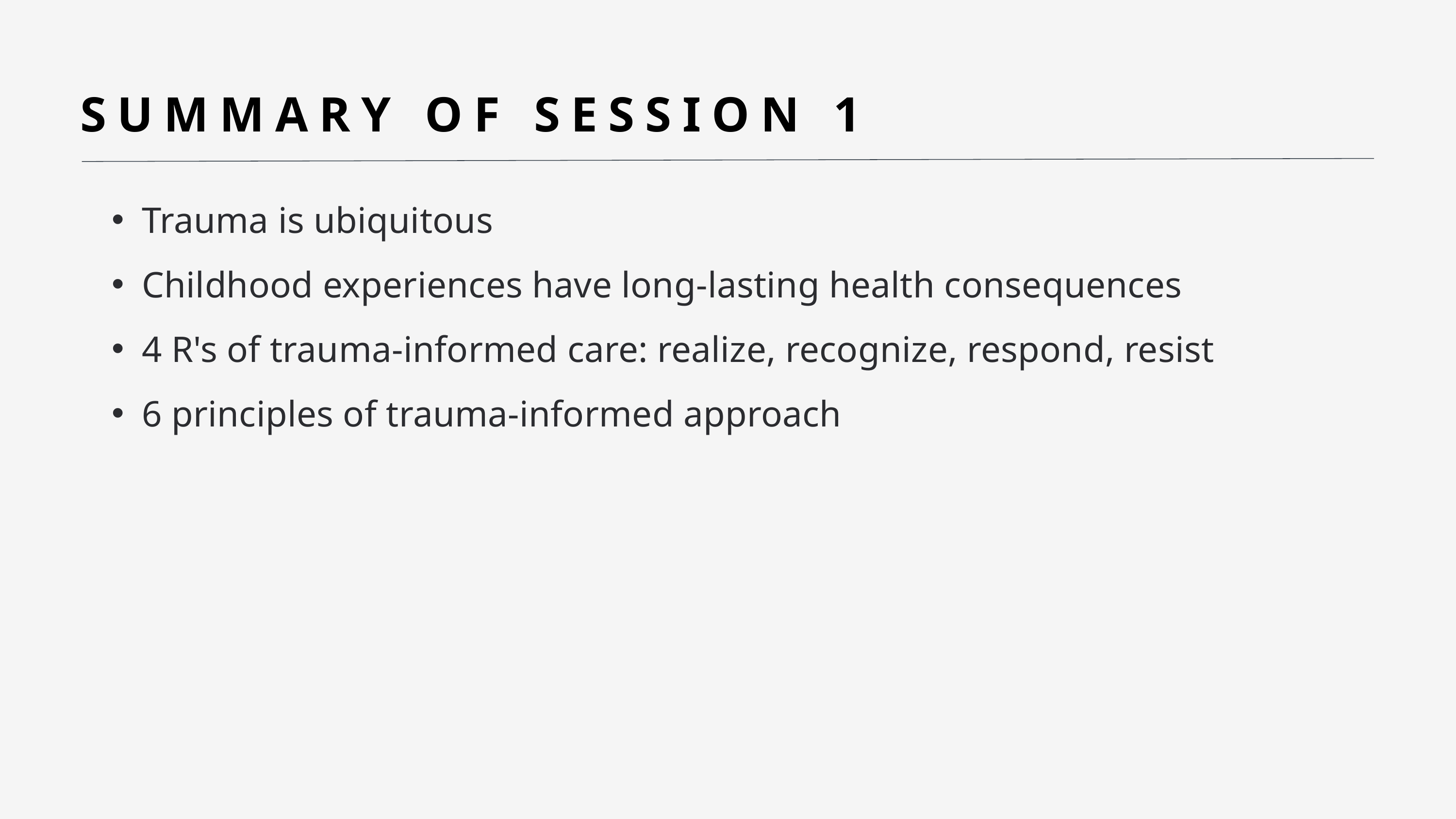

SUMMARY OF SESSION 1
Trauma is ubiquitous
Childhood experiences have long-lasting health consequences
4 R's of trauma-informed care: realize, recognize, respond, resist
6 principles of trauma-informed approach

## Slide 7
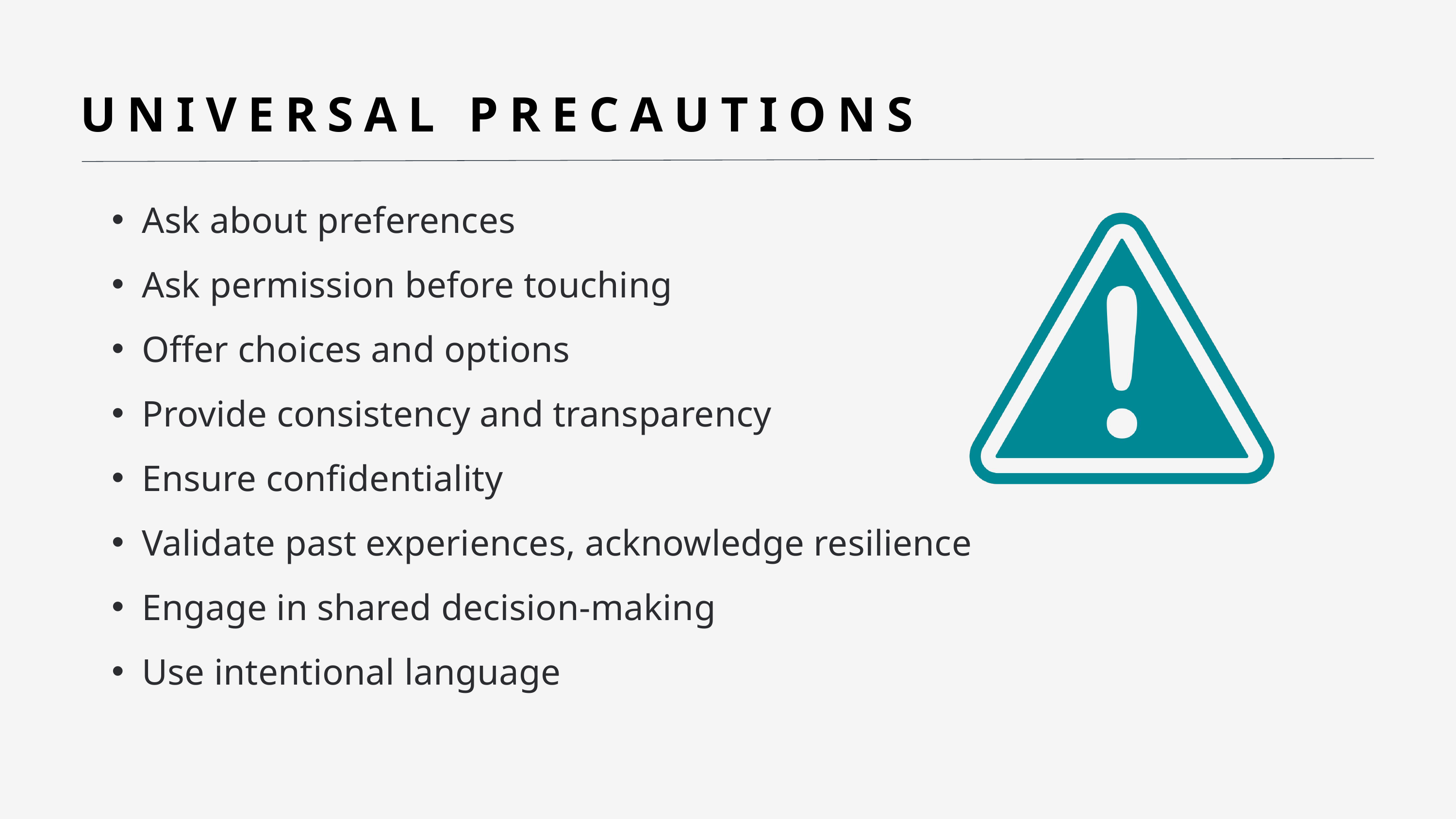

UNIVERSAL PRECAUTIONS
Ask about preferences
Ask permission before touching
Offer choices and options
Provide consistency and transparency
Ensure confidentiality
Validate past experiences, acknowledge resilience
Engage in shared decision-making
Use intentional language

## Slide 8
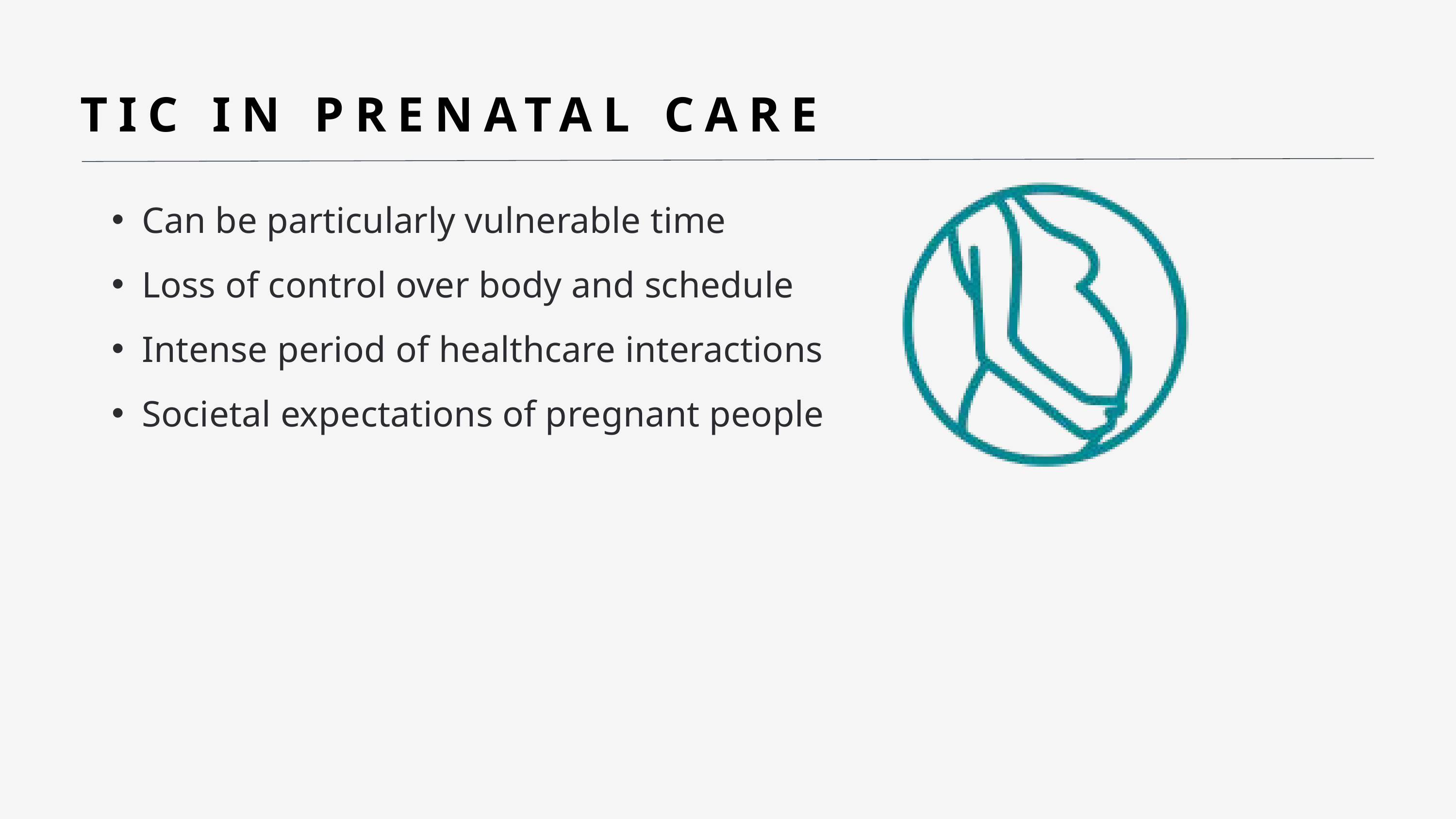

TIC IN PRENATAL CARE
Can be particularly vulnerable time
Loss of control over body and schedule
Intense period of healthcare interactions
Societal expectations of pregnant people

## Slide 9
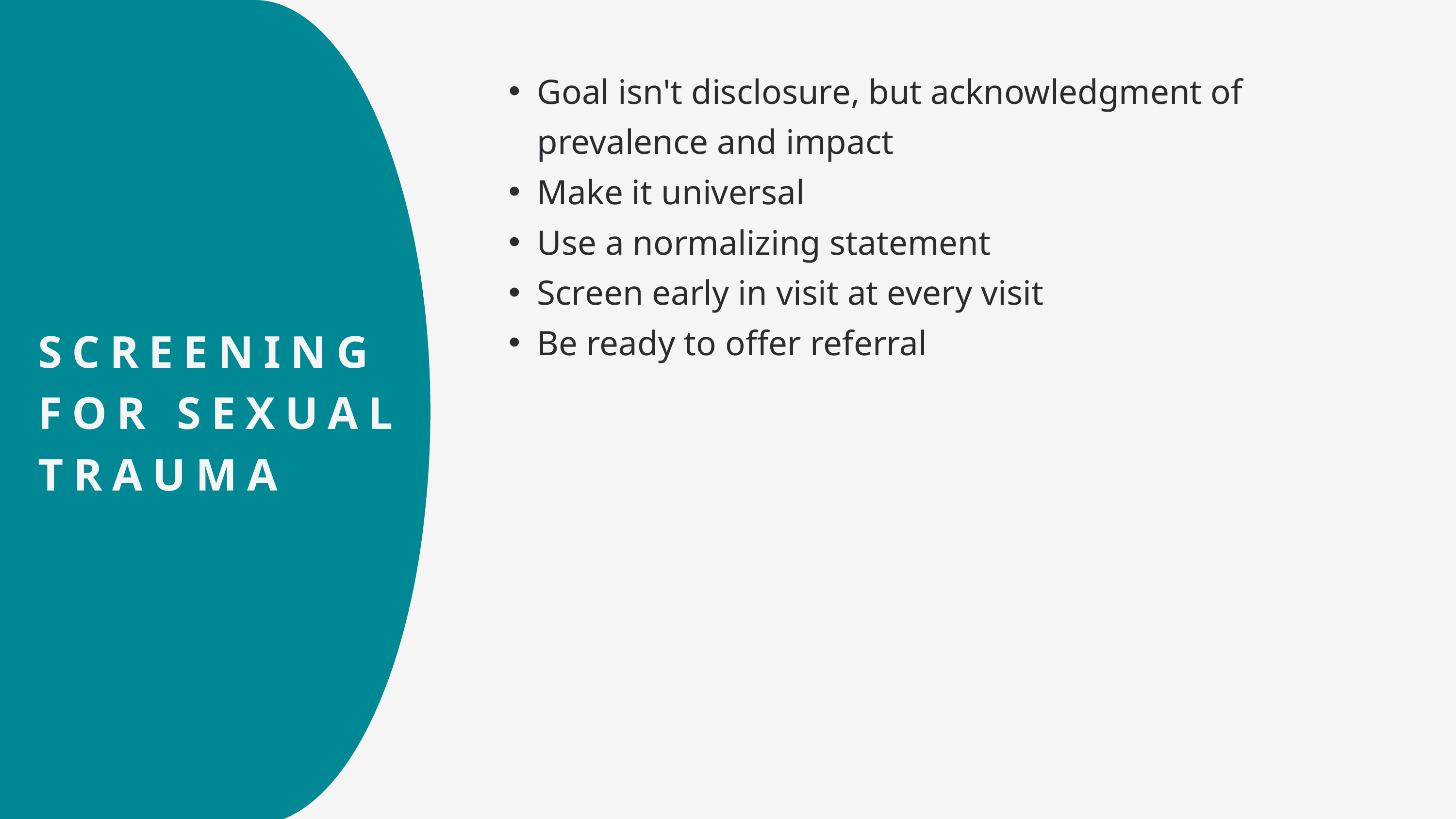

Goal isn't disclosure, but acknowledgment of prevalence and impact
Make it universal
Use a normalizing statement
Screen early in visit at every visit
Be ready to offer referral
SCREENING
FOR SEXUAL TRAUMA

## Slide 10
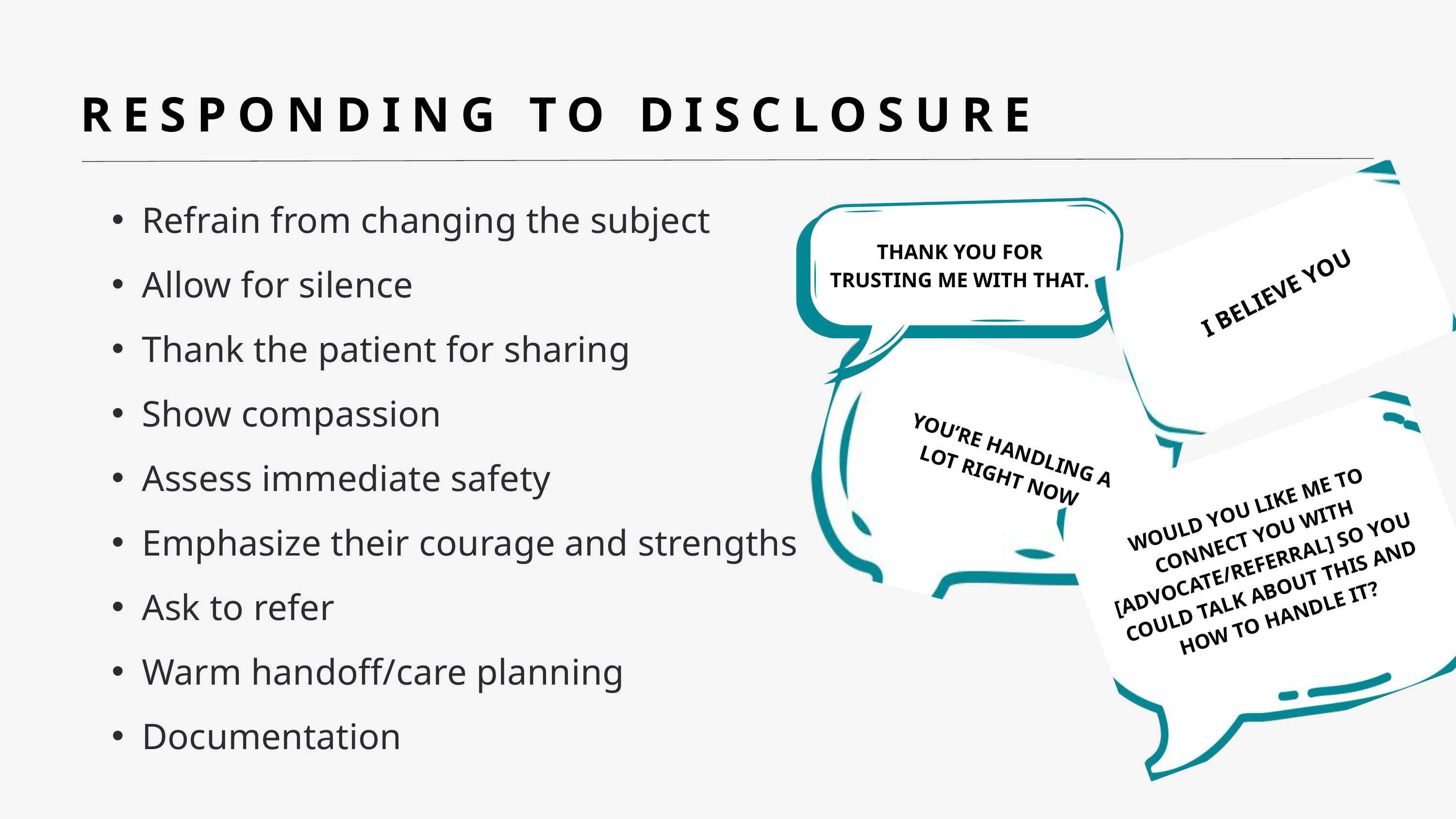

RESPONDING TO DISCLOSURE
Refrain from changing the subject
Allow for silence
Thank the patient for sharing
Show compassion
Assess immediate safety
Emphasize their courage and strengths
Ask to refer
Warm handoff/care planning
Documentation
THANK YOU FOR TRUSTING ME WITH THAT.
I BELIEVE YOU
YOU’RE HANDLING A LOT RIGHT NOW.
WOULD YOU LIKE ME TO CONNECT YOU WITH [ADVOCATE/REFERRAL] SO YOU COULD TALK ABOUT THIS AND HOW TO HANDLE IT?

## Slide 11
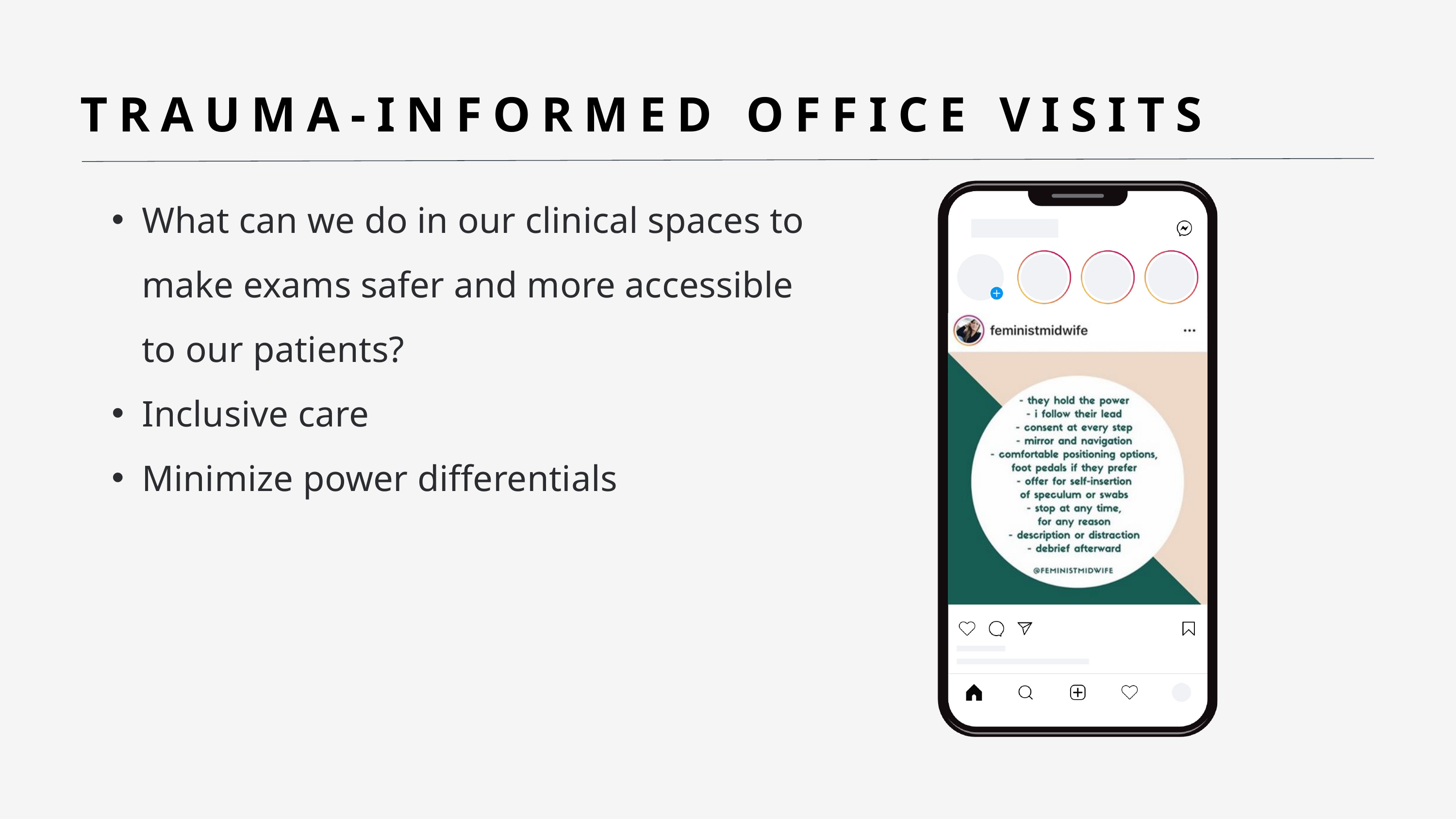

TRAUMA-INFORMED OFFICE VISITS
What can we do in our clinical spaces to make exams safer and more accessible to our patients?
Inclusive care
Minimize power differentials

## Slide 12
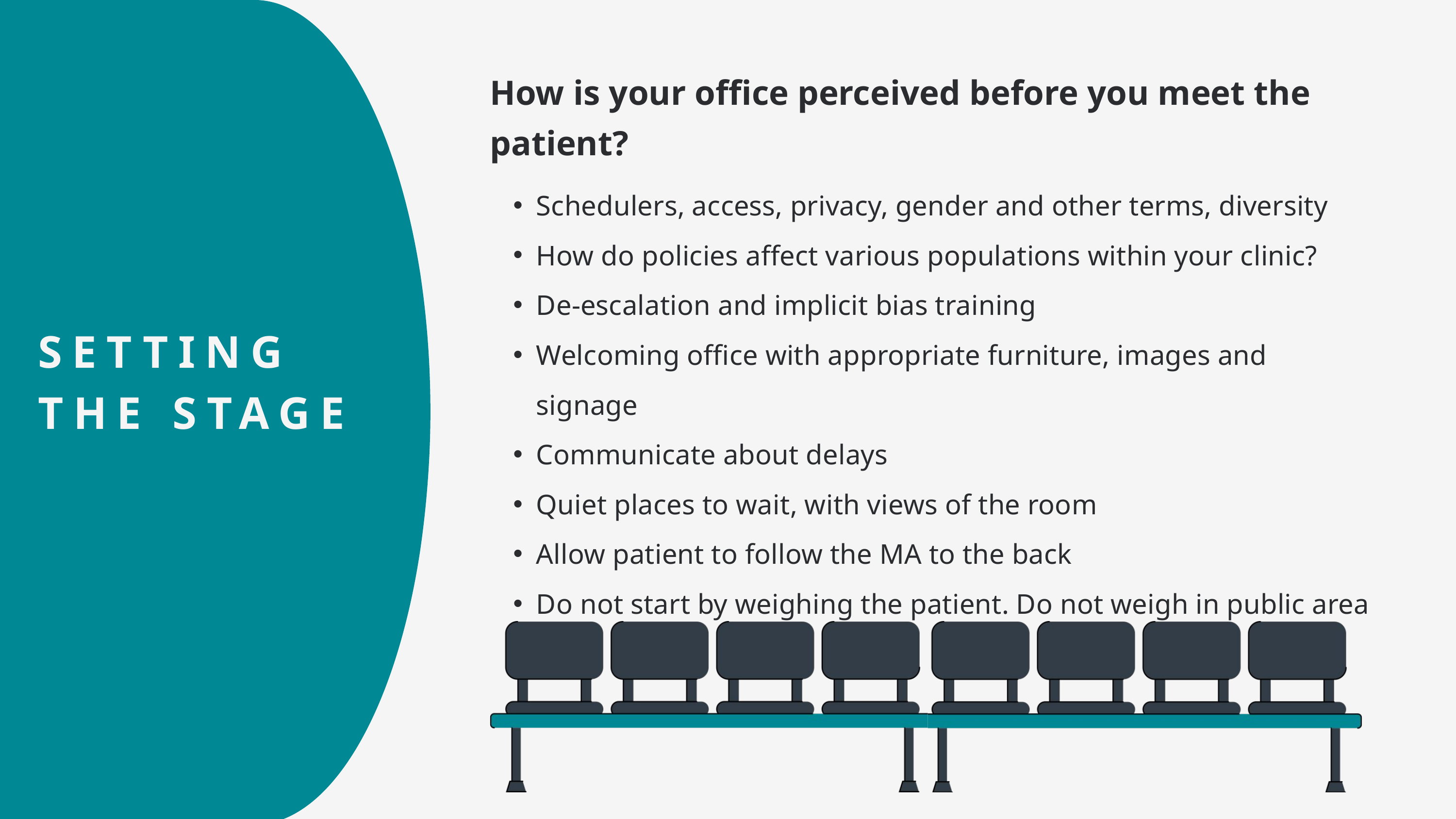

How is your office perceived before you meet the patient?
Schedulers, access, privacy, gender and other terms, diversity
How do policies affect various populations within your clinic?
De-escalation and implicit bias training
Welcoming office with appropriate furniture, images and signage
Communicate about delays
Quiet places to wait, with views of the room
Allow patient to follow the MA to the back
Do not start by weighing the patient. Do not weigh in public area
SETTING THE STAGE

## Slide 13
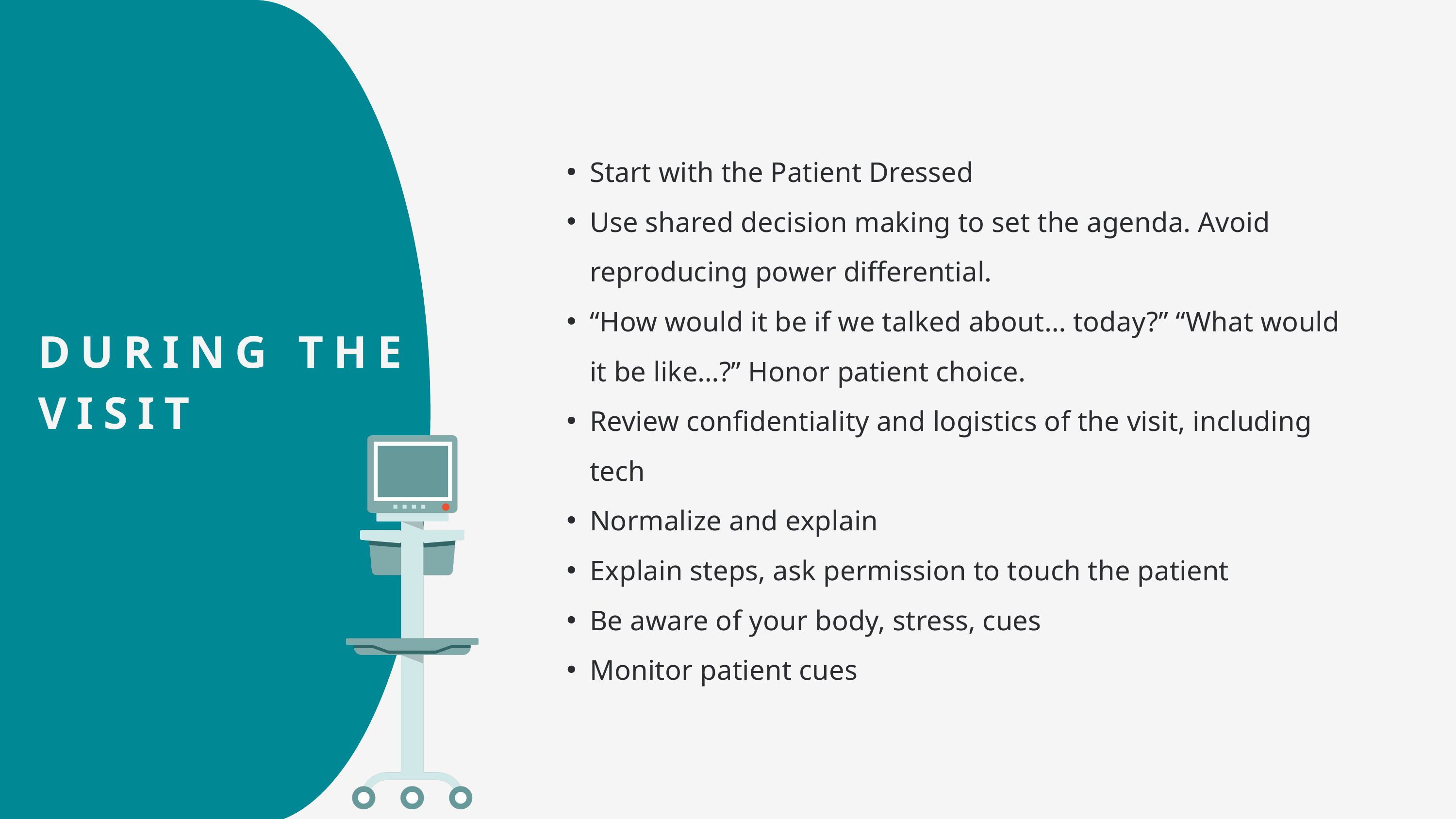

Start with the Patient Dressed
Use shared decision making to set the agenda. Avoid reproducing power differential.
“How would it be if we talked about… today?” “What would it be like…?” Honor patient choice.
Review confidentiality and logistics of the visit, including tech
Normalize and explain
Explain steps, ask permission to touch the patient
Be aware of your body, stress, cues
Monitor patient cues
DURING THE VISIT

## Slide 14
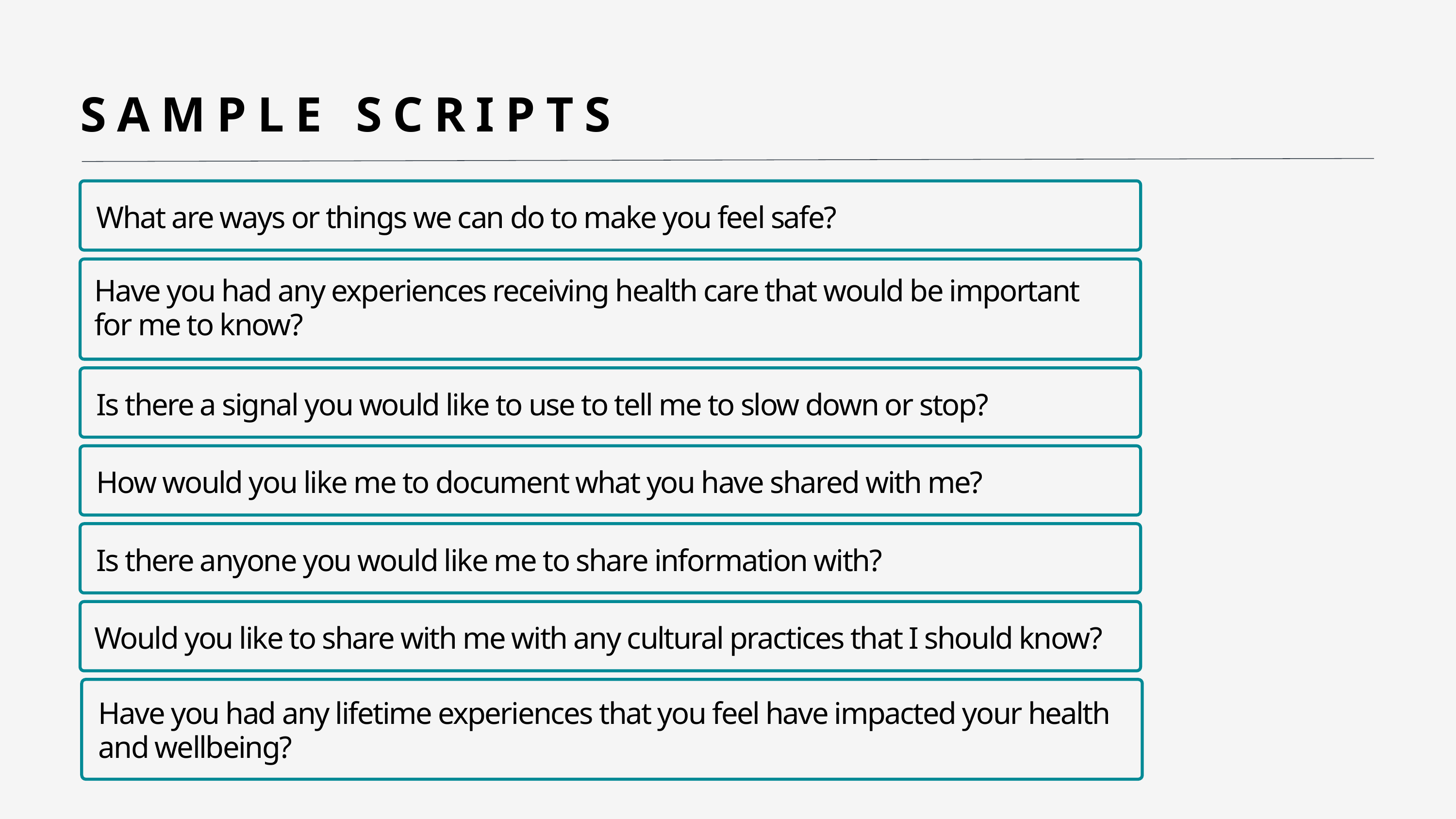

SAMPLE SCRIPTS
What are ways or things we can do to make you feel safe?
Have you had any experiences receiving health care that would be important for me to know?
Is there a signal you would like to use to tell me to slow down or stop?
How would you like me to document what you have shared with me?
Is there anyone you would like me to share information with?
Would you like to share with me with any cultural practices that I should know?
Have you had any lifetime experiences that you feel have impacted your health and wellbeing?

## Slide 15
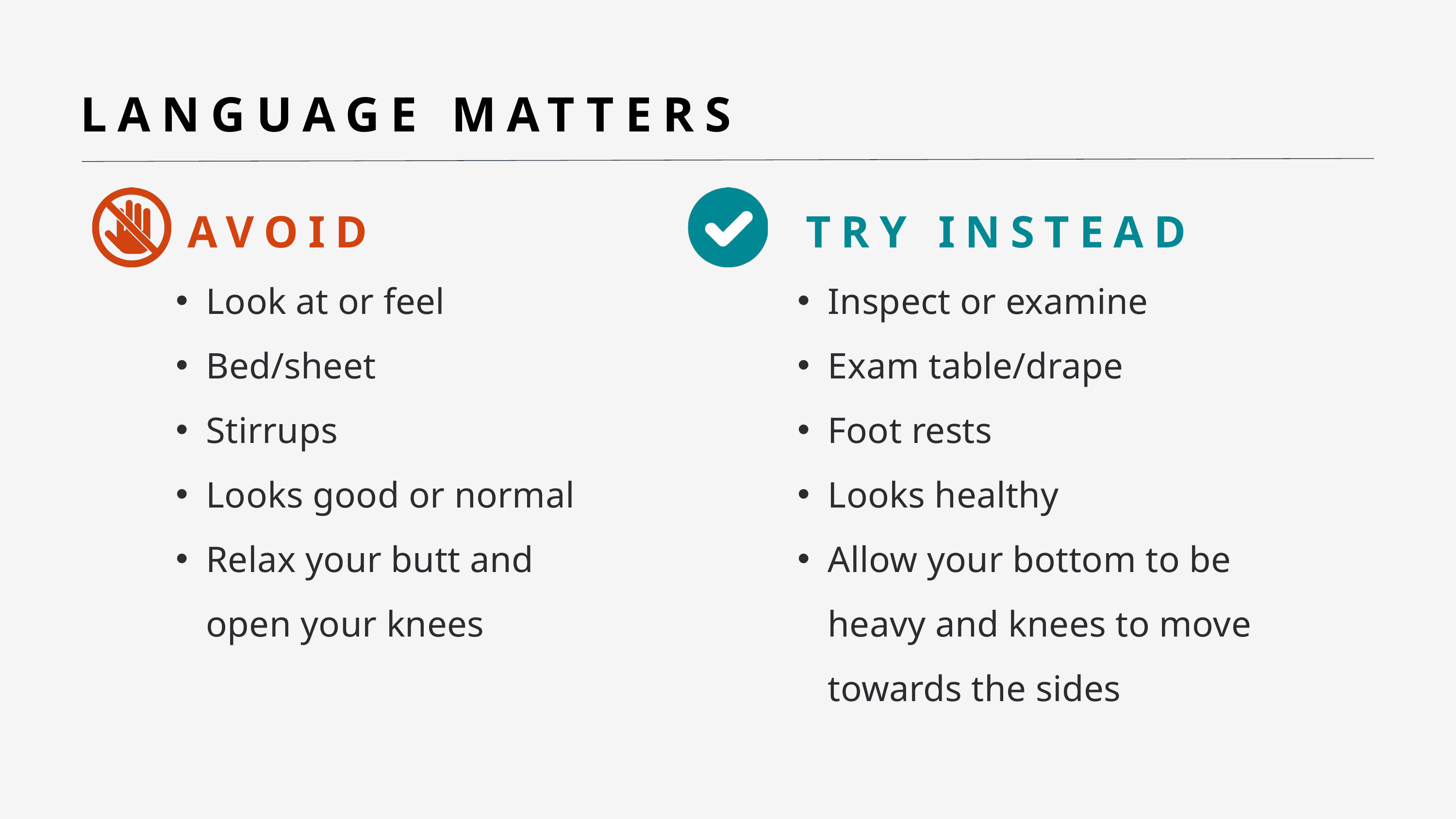

LANGUAGE MATTERS
AVOID
TRY INSTEAD
Look at or feel
Bed/sheet
Stirrups
Looks good or normal
Relax your butt and open your knees
Inspect or examine
Exam table/drape
Foot rests
Looks healthy
Allow your bottom to be heavy and knees to move towards the sides

## Slide 16
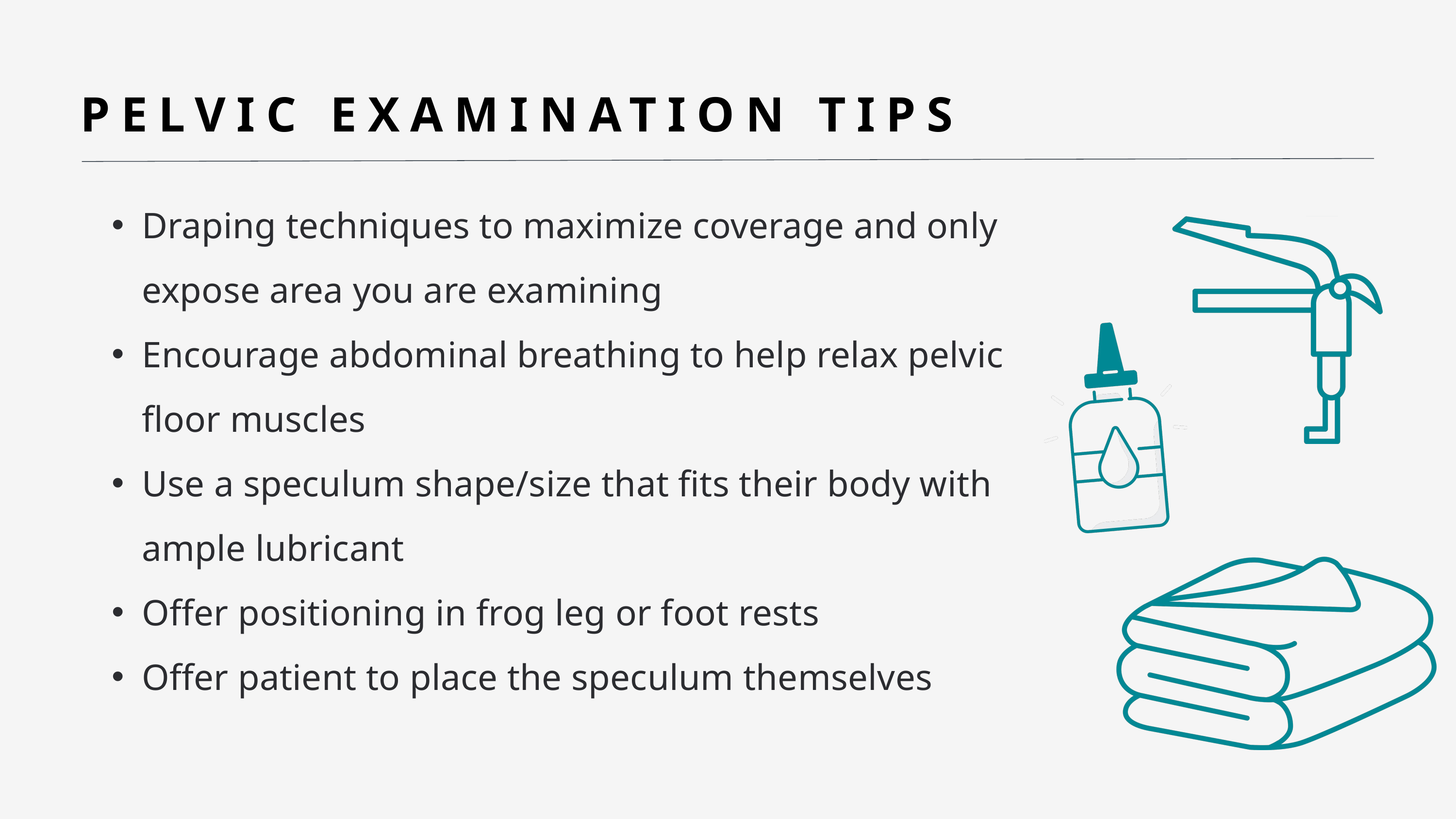

PELVIC EXAMINATION TIPS
Draping techniques to maximize coverage and only expose area you are examining
Encourage abdominal breathing to help relax pelvic floor muscles
Use a speculum shape/size that fits their body with ample lubricant
Offer positioning in frog leg or foot rests
Offer patient to place the speculum themselves

## Slide 17
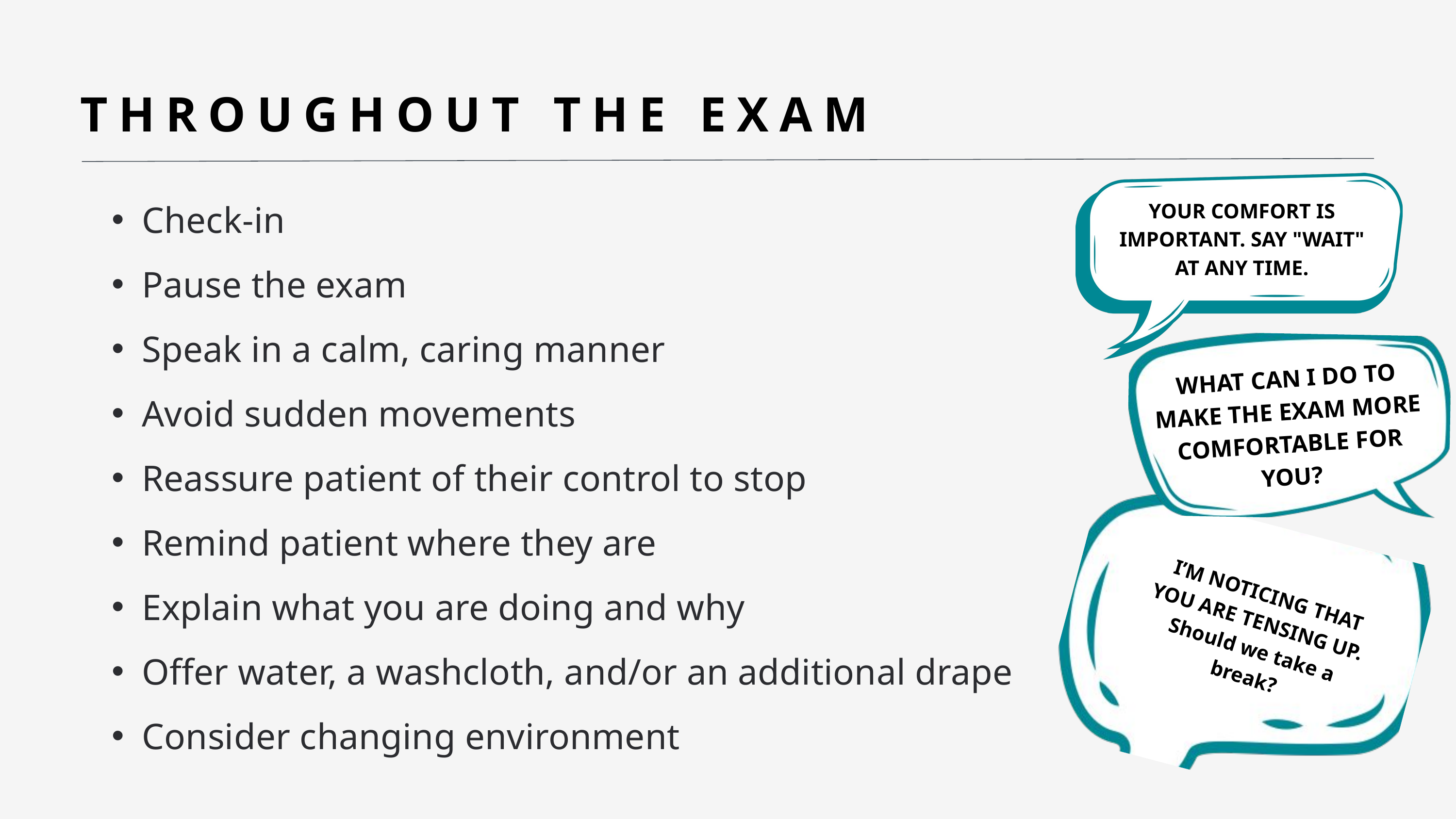

THROUGHOUT THE EXAM
Check-in
Pause the exam
Speak in a calm, caring manner
Avoid sudden movements
Reassure patient of their control to stop
Remind patient where they are
Explain what you are doing and why
Offer water, a washcloth, and/or an additional drape
Consider changing environment
YOUR COMFORT IS IMPORTANT. SAY "WAIT" AT ANY TIME.
WHAT CAN I DO TO MAKE THE EXAM MORE COMFORTABLE FOR YOU?
I’M NOTICING THAT YOU ARE TENSING UP.
Should we take a break?

## Slide 18
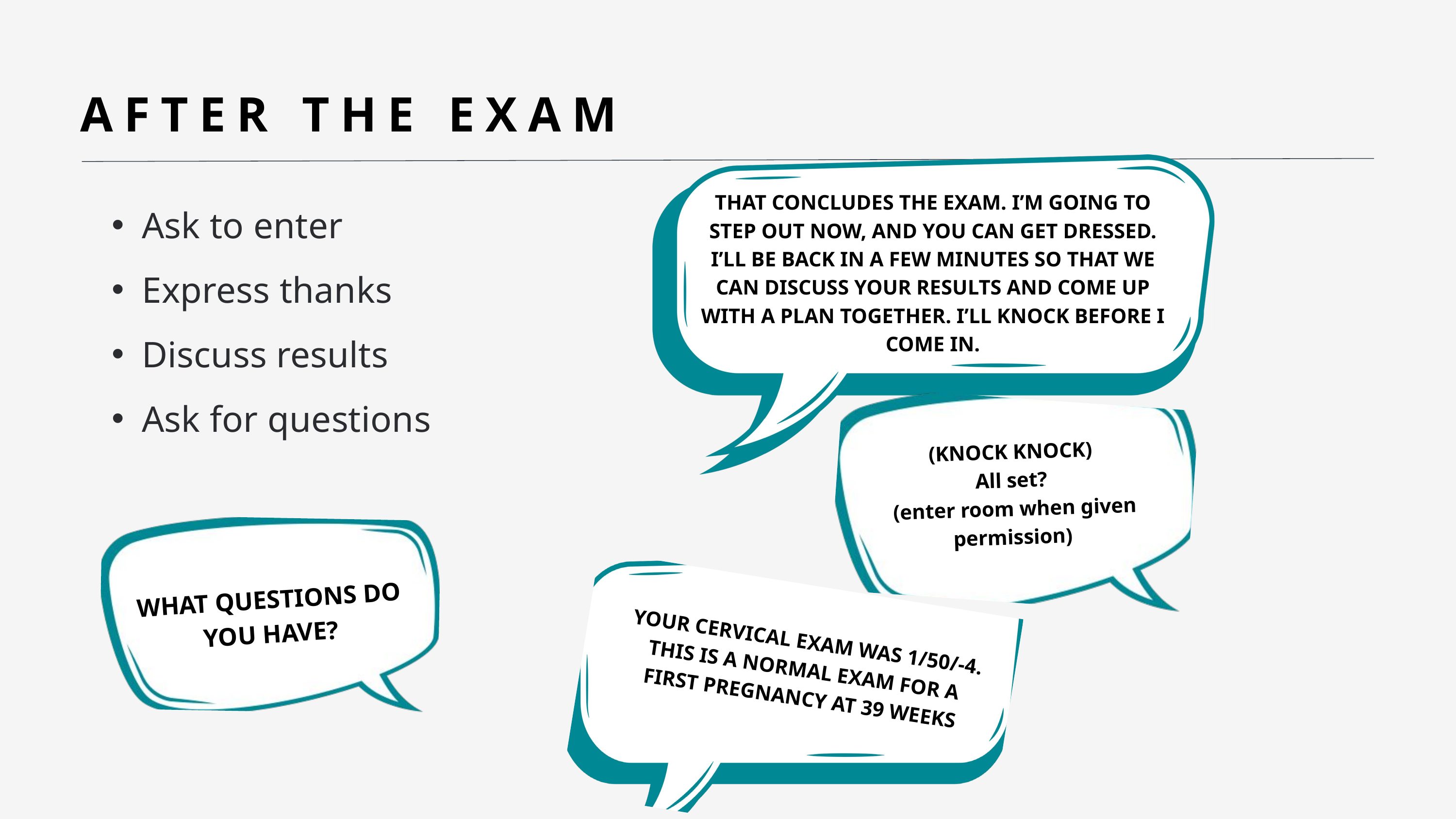

AFTER THE EXAM
Ask to enter
Express thanks
Discuss results
Ask for questions
THAT CONCLUDES THE EXAM. I’M GOING TO STEP OUT NOW, AND YOU CAN GET DRESSED. I’LL BE BACK IN A FEW MINUTES SO THAT WE CAN DISCUSS YOUR RESULTS AND COME UP WITH A PLAN TOGETHER. I’LL KNOCK BEFORE I COME IN.​
(KNOCK KNOCK)
All set?
(enter room when given permission)
WHAT QUESTIONS DO YOU HAVE?
YOUR CERVICAL EXAM WAS 1/50/-4. THIS IS A NORMAL EXAM FOR A FIRST PREGNANCY AT 39 WEEKS

## Slide 19
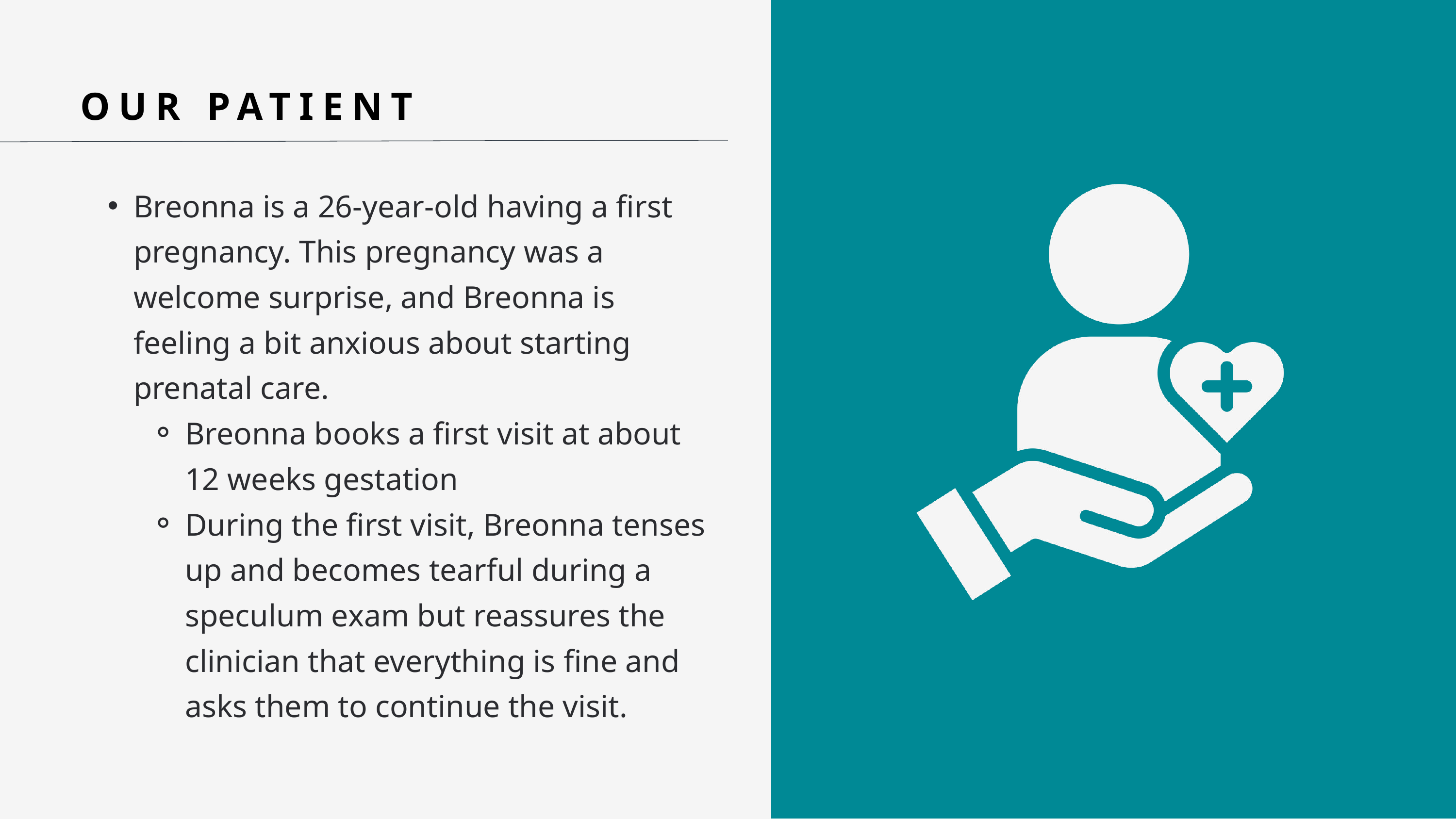

OUR PATIENT
Breonna is a 26-year-old having a first pregnancy. This pregnancy was a welcome surprise, and Breonna is feeling a bit anxious about starting prenatal care.
Breonna books a first visit at about 12 weeks gestation
During the first visit, Breonna tenses up and becomes tearful during a speculum exam but reassures the clinician that everything is fine and asks them to continue the visit.

## Slide 20
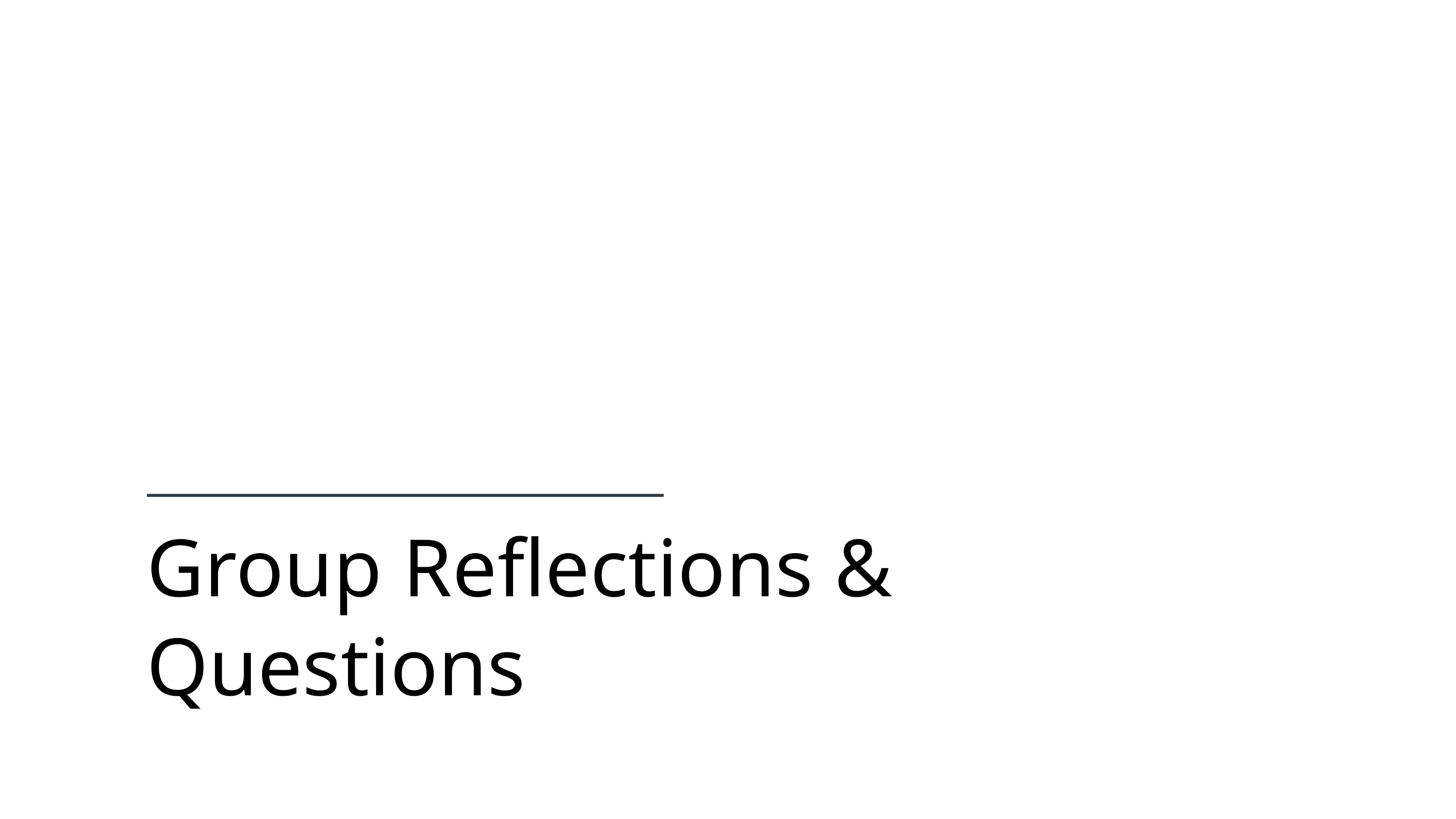

Group Reflections & Questions

## Slide 21
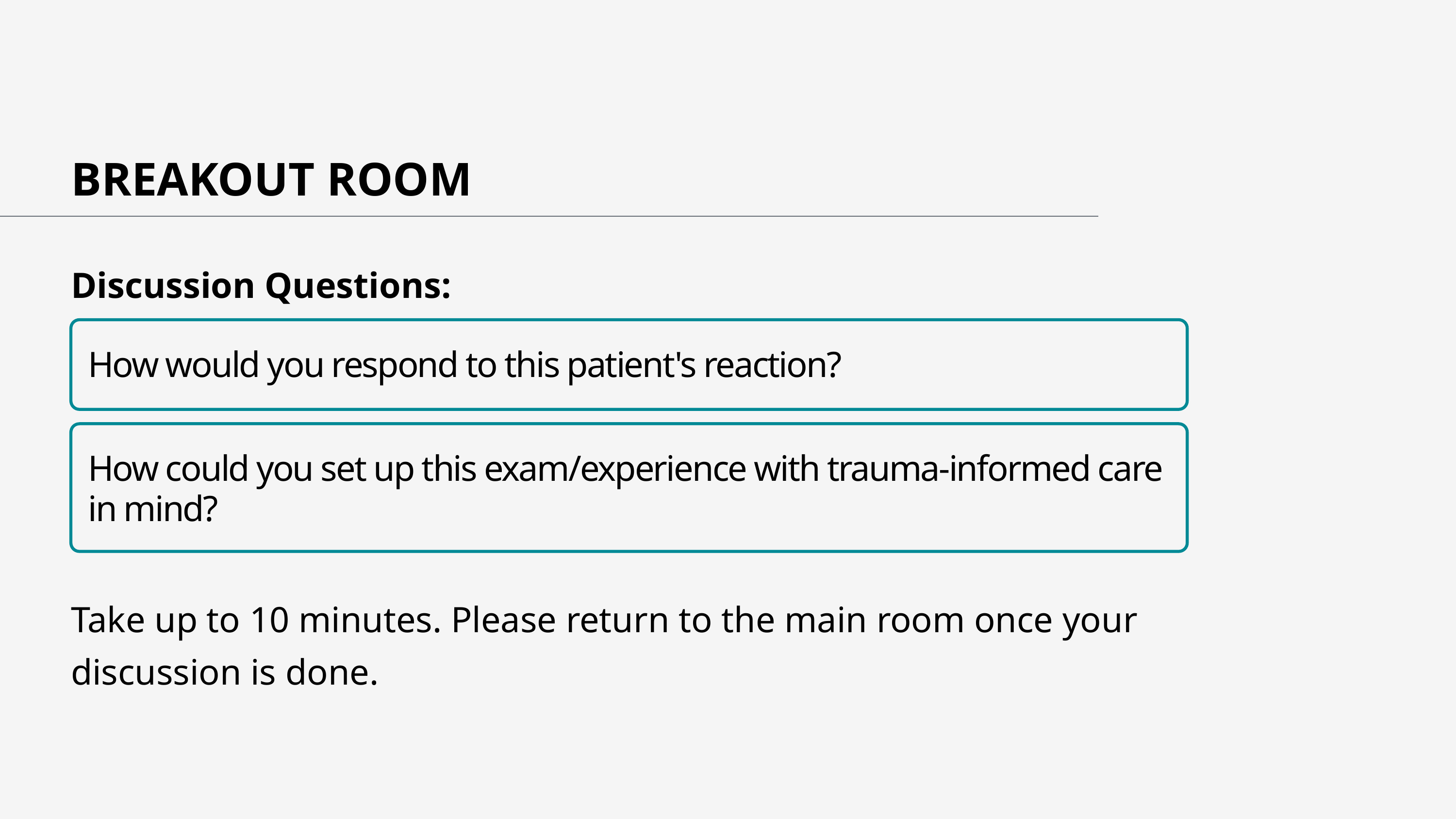

BREAKOUT ROOM
Discussion Questions:
How would you respond to this patient's reaction?
How could you set up this exam/experience with trauma-informed care in mind?
Take up to 10 minutes. Please return to the main room once your discussion is done.

## Slide 22
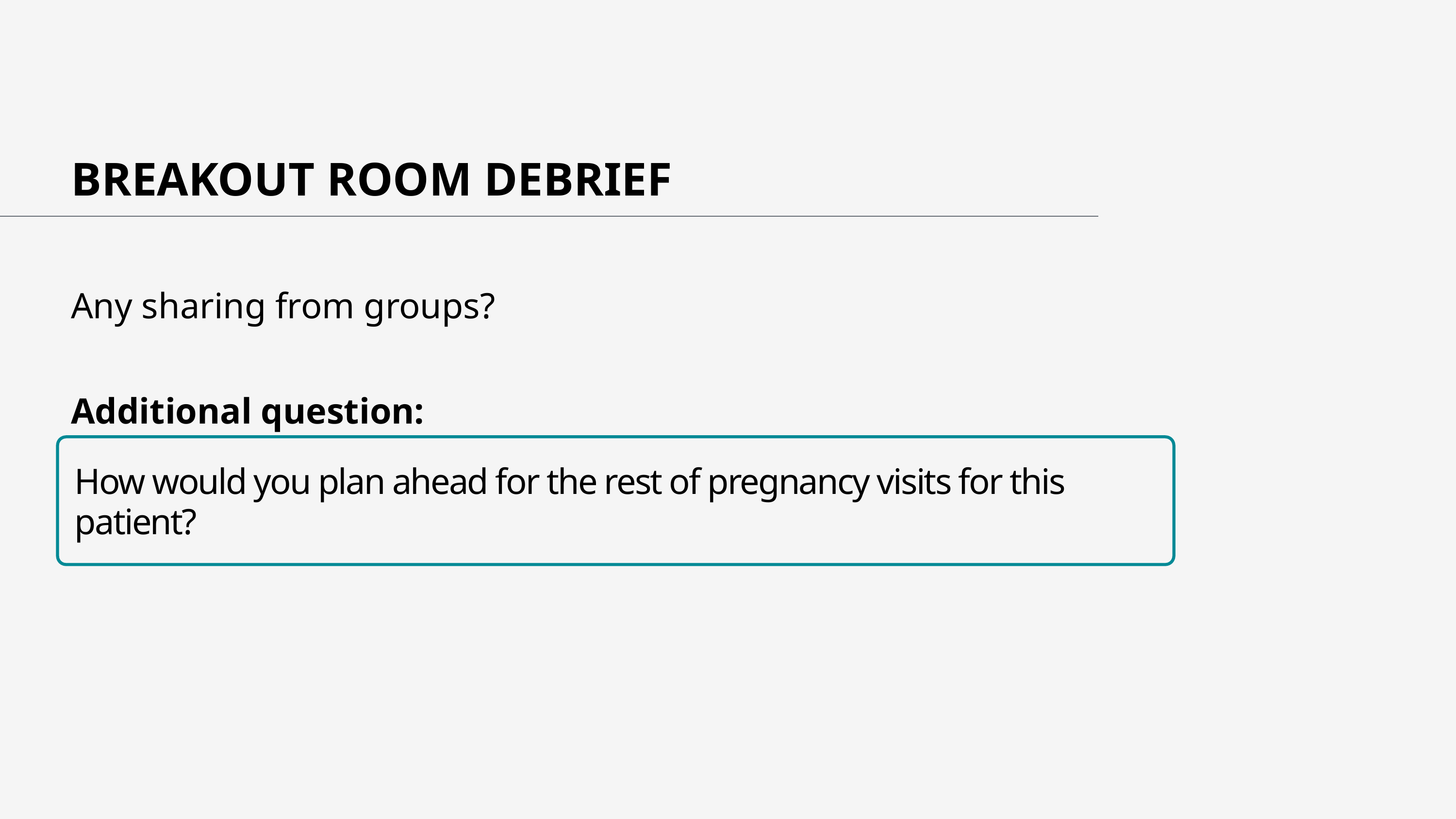

BREAKOUT ROOM DEBRIEF
Any sharing from groups?
Additional question:
How would you plan ahead for the rest of pregnancy visits for this patient?

## Slide 23
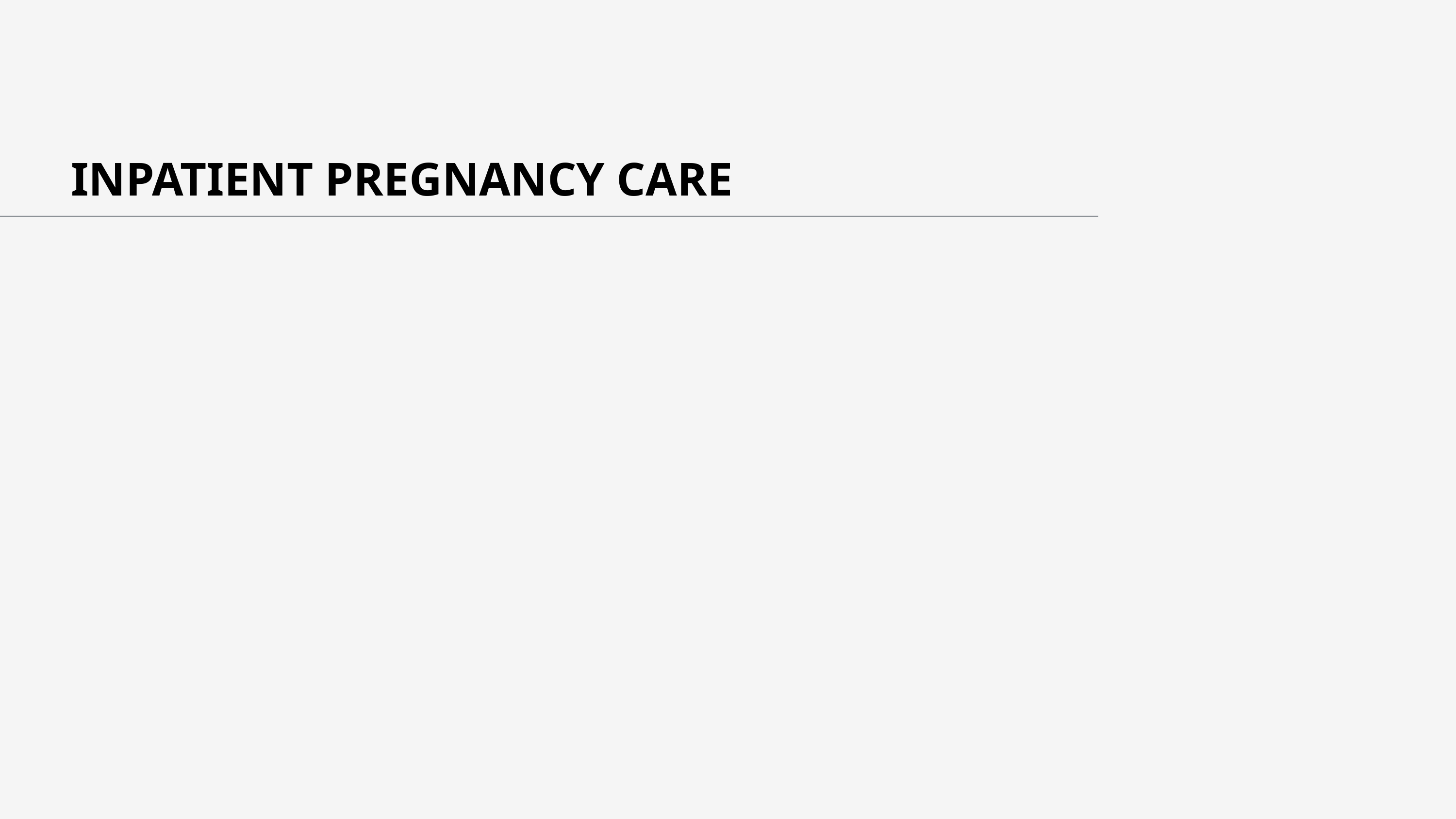

INPATIENT PREGNANCY CARE

## Slide 24
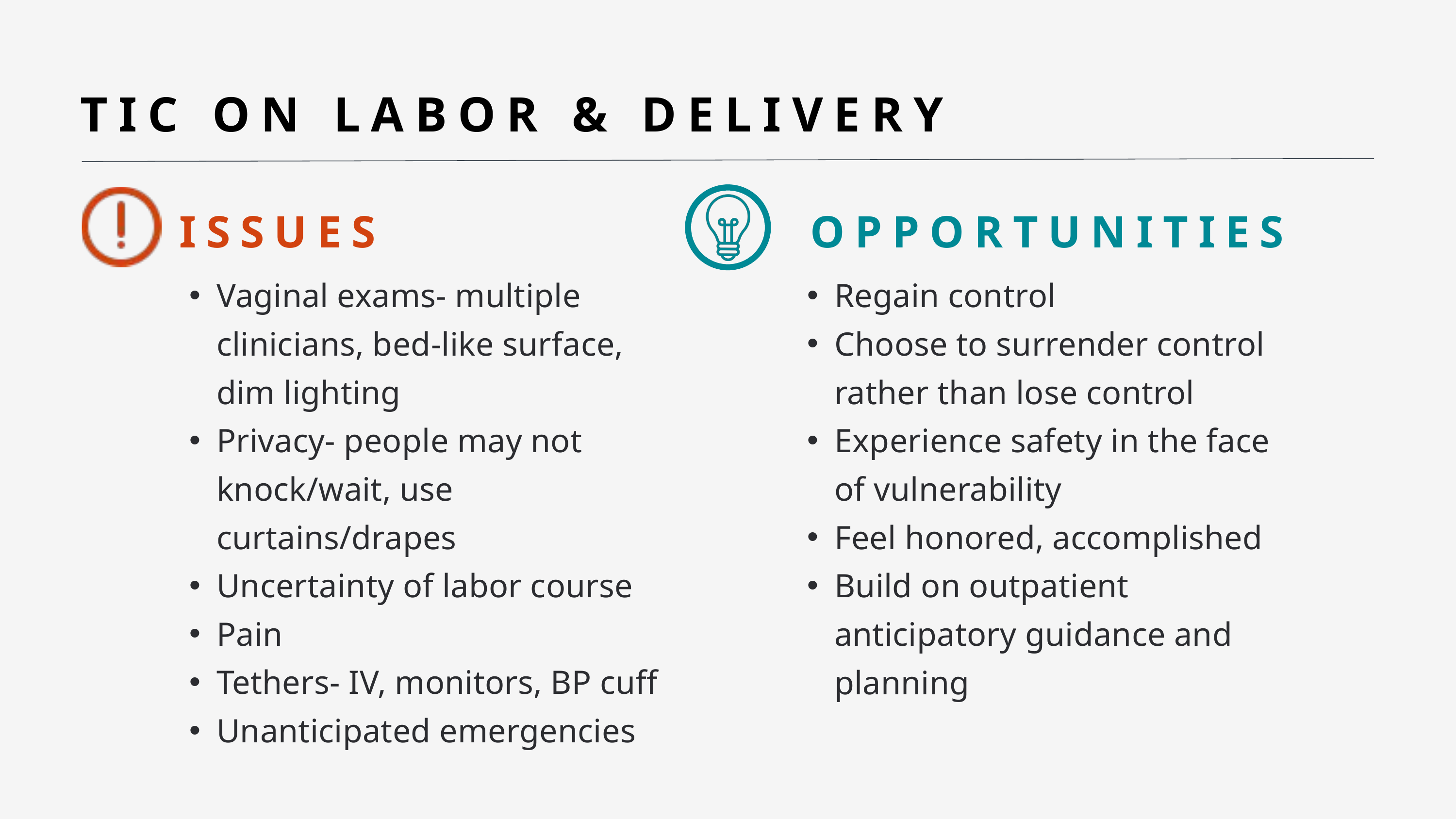

TIC ON LABOR & DELIVERY
ISSUES
OPPORTUNITIES
Vaginal exams- multiple clinicians, bed-like surface, dim lighting
Privacy- people may not knock/wait, use curtains/drapes
Uncertainty of labor course
Pain
Tethers- IV, monitors, BP cuff
Unanticipated emergencies
Regain control
Choose to surrender control rather than lose control
Experience safety in the face of vulnerability
Feel honored, accomplished
Build on outpatient anticipatory guidance and planning

## Slide 25
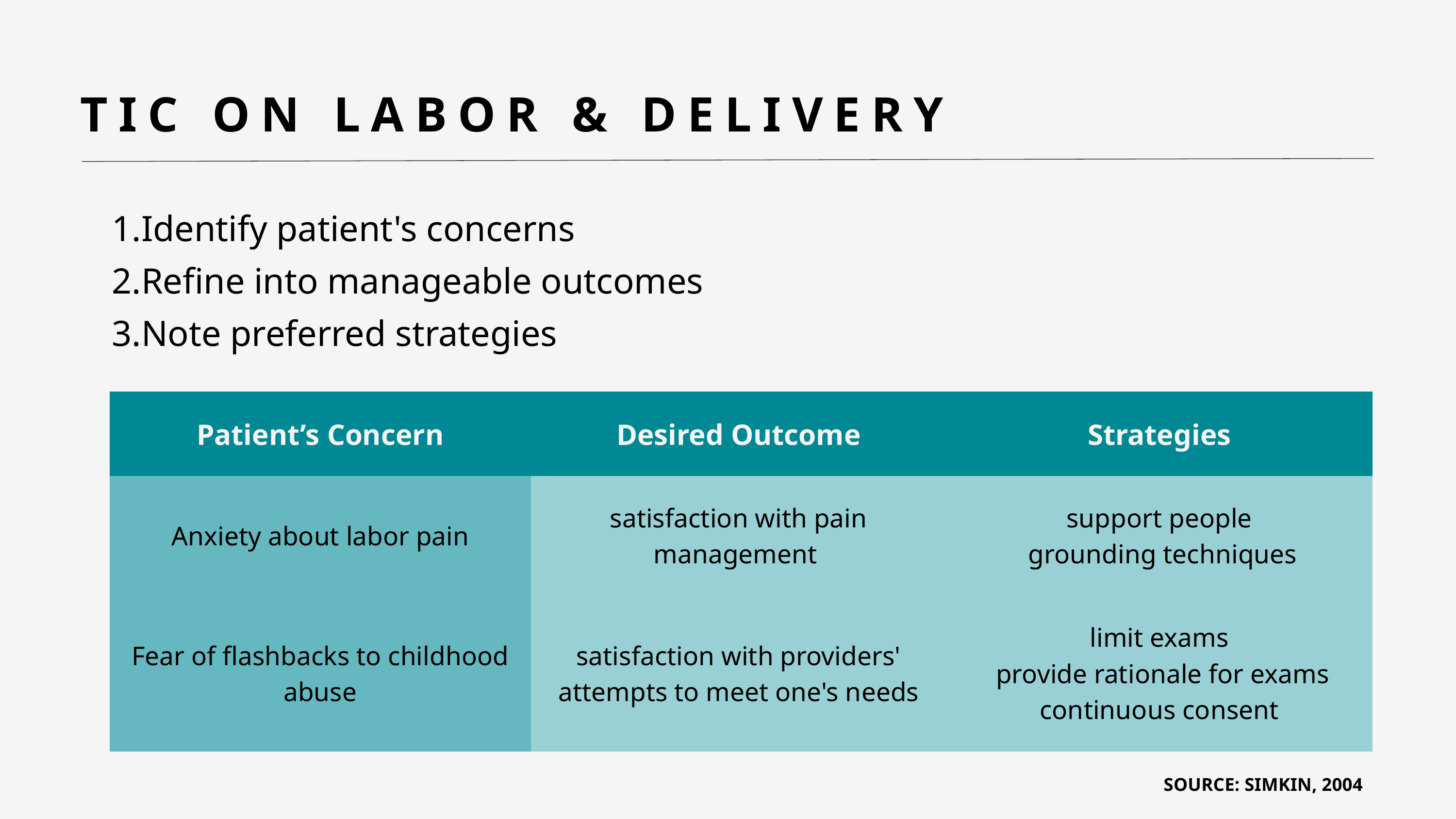

TIC ON LABOR & DELIVERY
Identify patient's concerns
Refine into manageable outcomes
Note preferred strategies
| Patient’s Concern | Desired Outcome | Strategies |
| --- | --- | --- |
| Anxiety about labor pain | satisfaction with pain management | support people grounding techniques |
| Fear of flashbacks to childhood abuse | satisfaction with providers' attempts to meet one's needs | limit exams provide rationale for exams continuous consent |
SOURCE: SIMKIN, 2004

## Slide 26
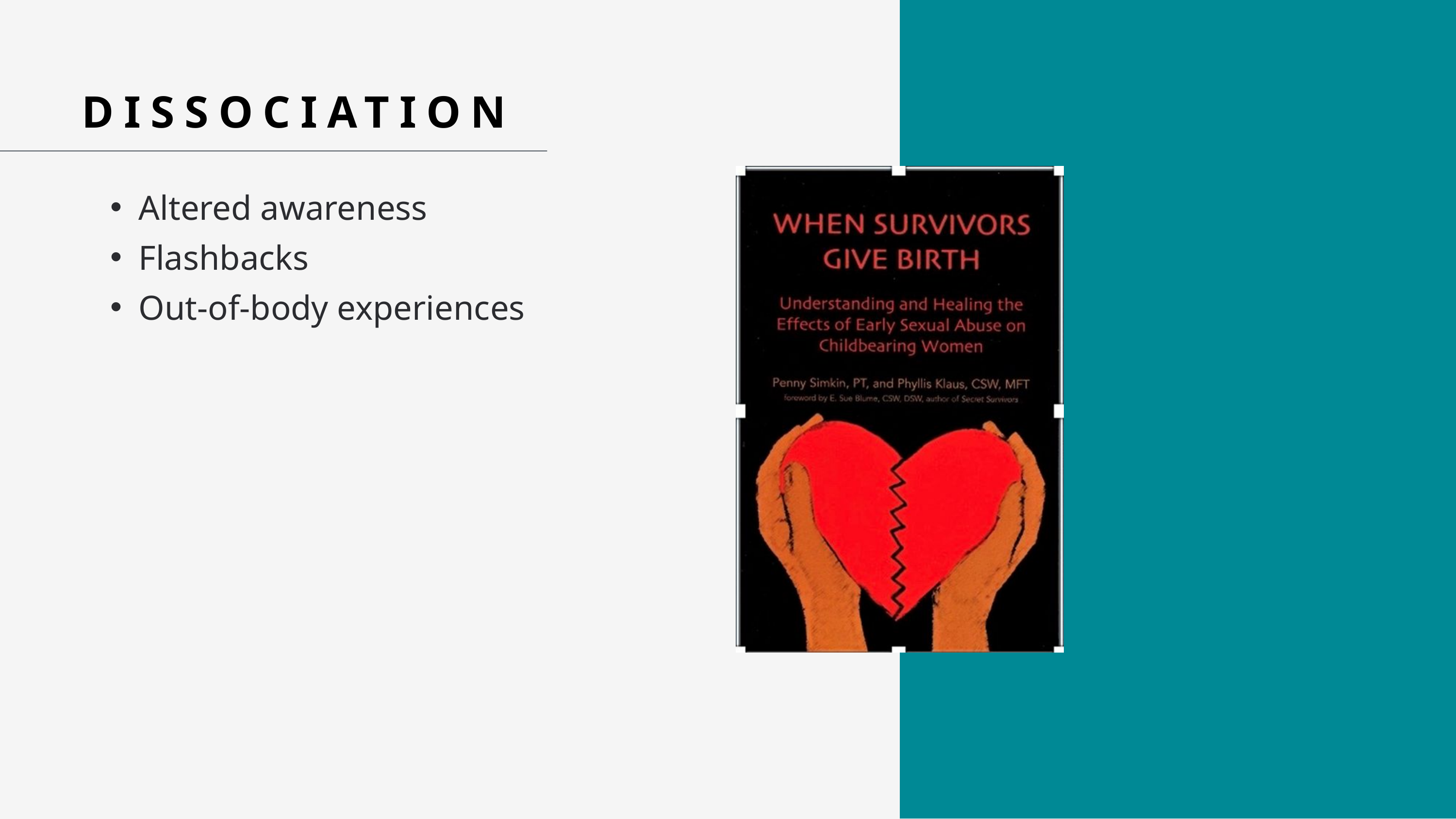

DISSOCIATION
Altered awareness
Flashbacks
Out-of-body experiences

## Slide 27
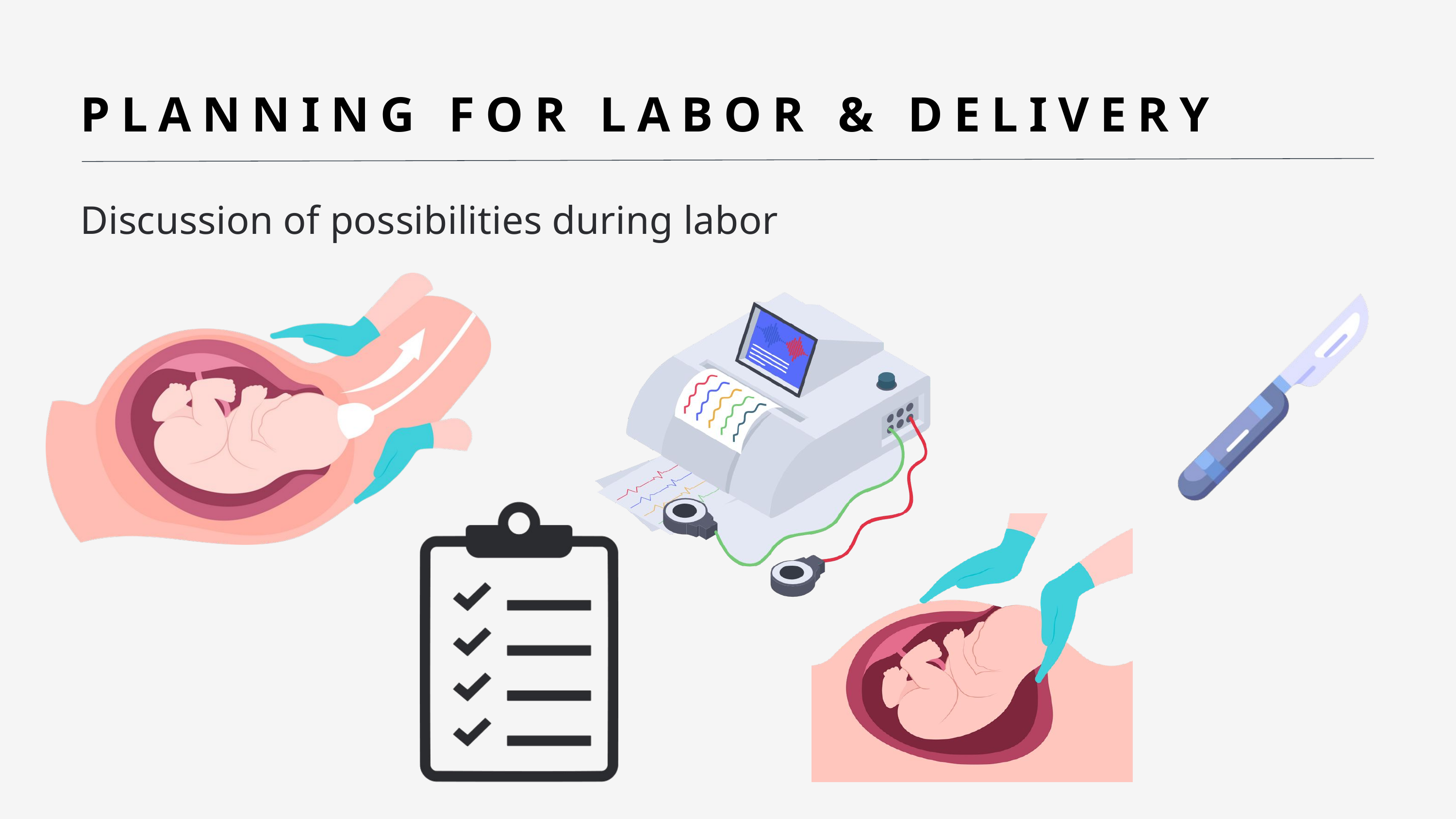

PLANNING FOR LABOR & DELIVERY
Discussion of possibilities during labor

## Slide 28
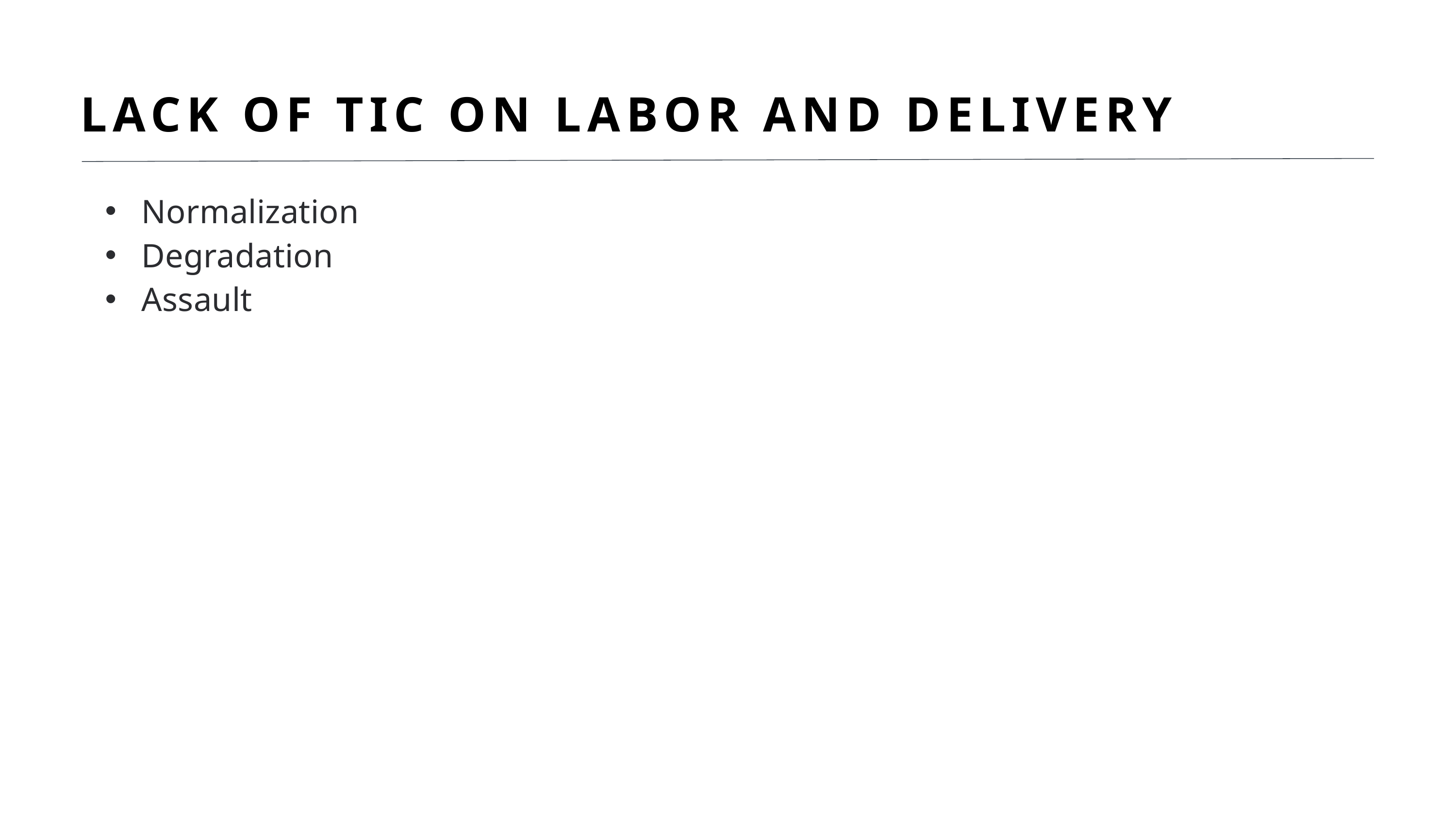

LACK OF TIC ON LABOR AND DELIVERY
Normalization
Degradation
Assault

## Slide 29
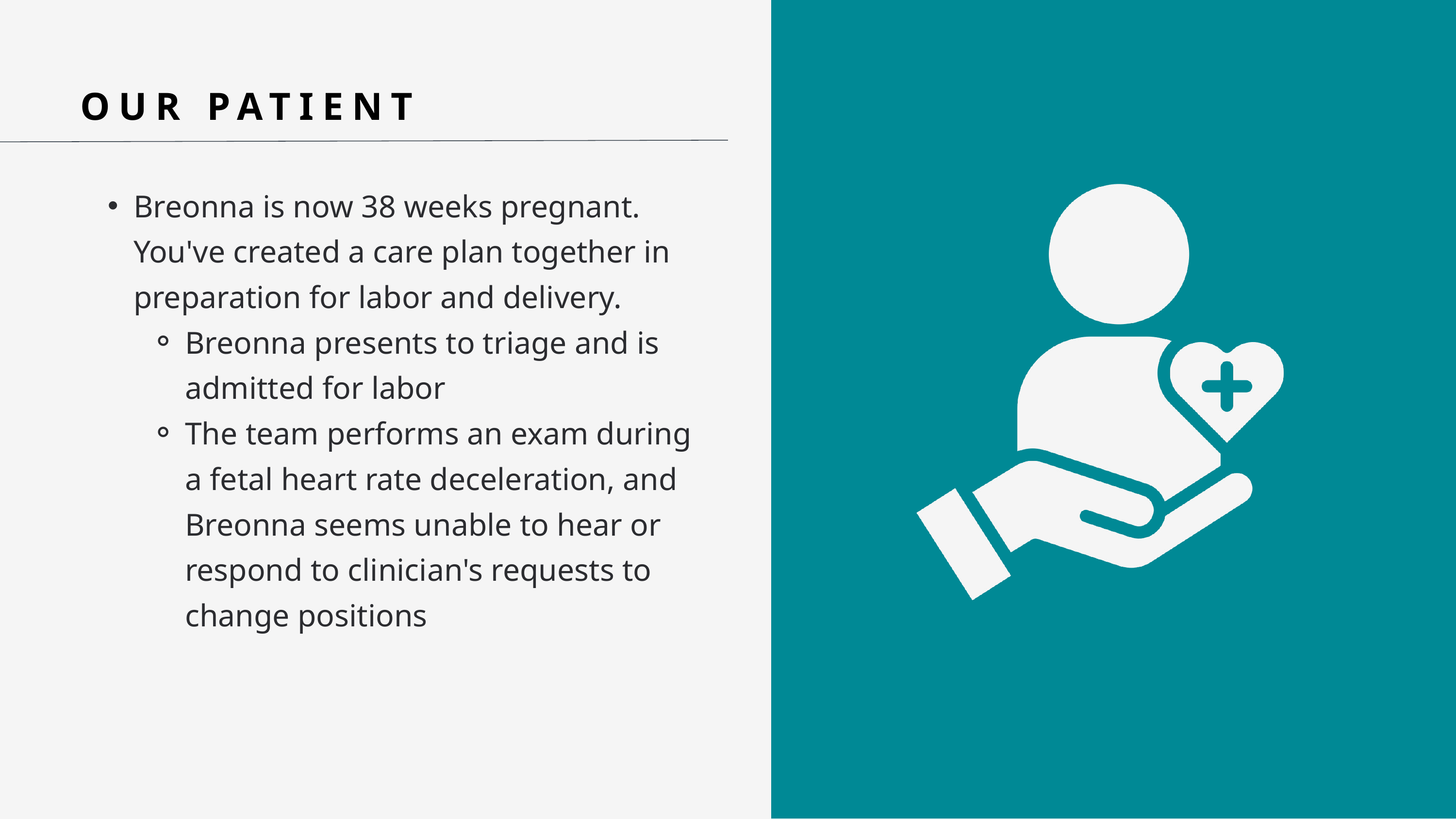

OUR PATIENT
Breonna is now 38 weeks pregnant. You've created a care plan together in preparation for labor and delivery.
Breonna presents to triage and is admitted for labor
The team performs an exam during a fetal heart rate deceleration, and Breonna seems unable to hear or respond to clinician's requests to change positions

## Slide 30
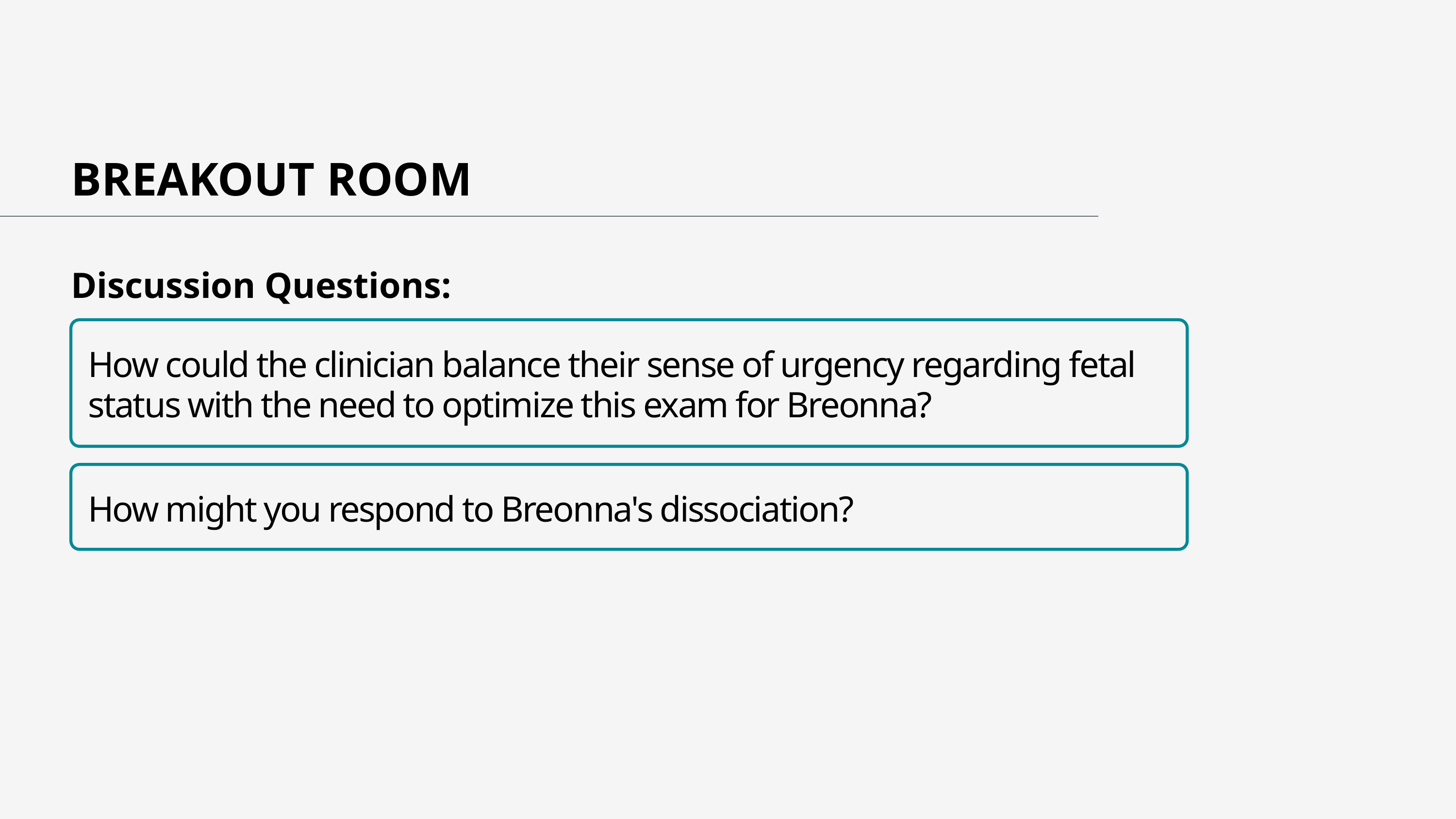

BREAKOUT ROOM
Discussion Questions:
How could the clinician balance their sense of urgency regarding fetal status with the need to optimize this exam for Breonna?
How might you respond to Breonna's dissociation?

## Slide 31
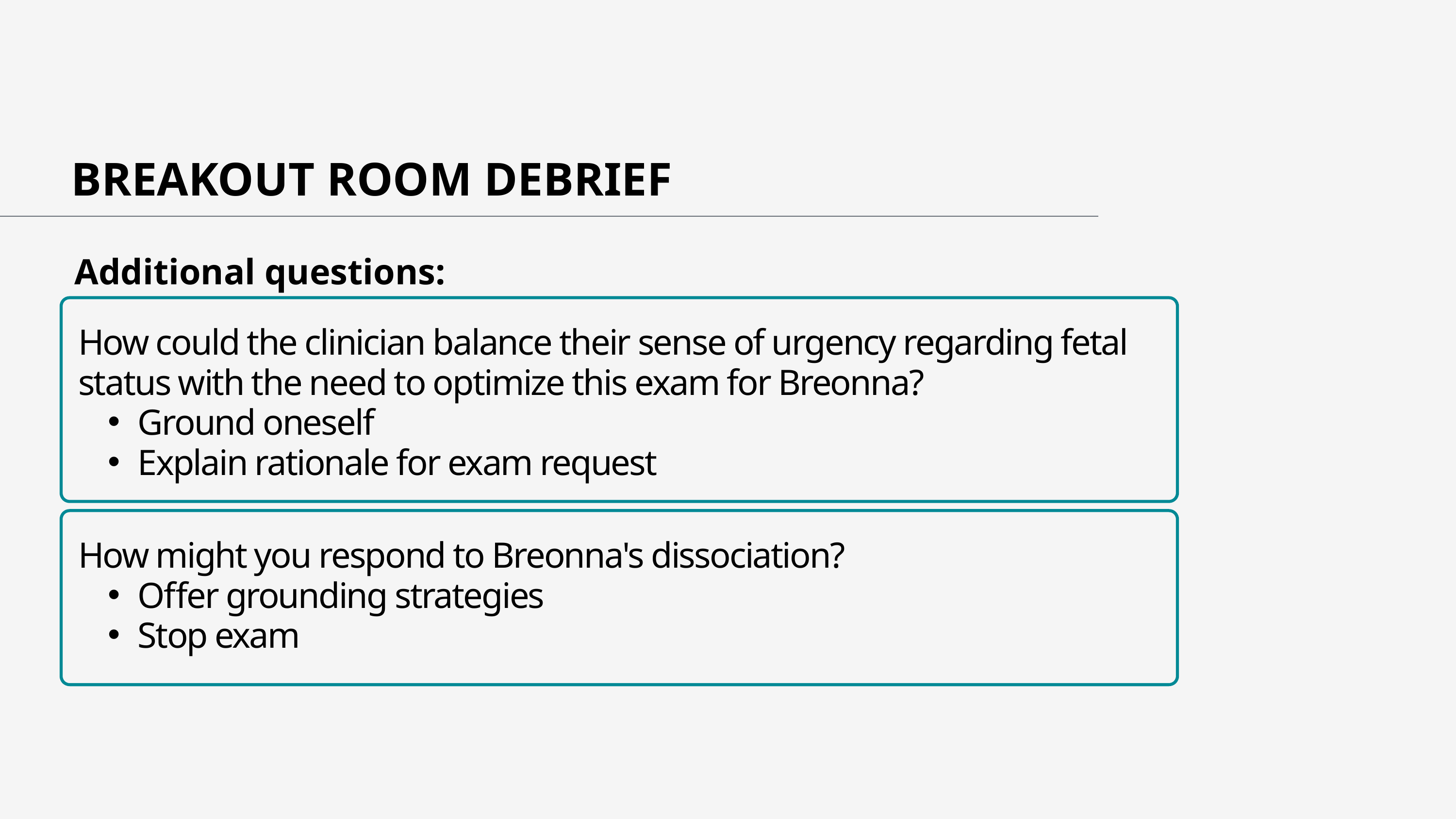

BREAKOUT ROOM DEBRIEF
Additional questions:
How could the clinician balance their sense of urgency regarding fetal status with the need to optimize this exam for Breonna?
Ground oneself
Explain rationale for exam request
How might you respond to Breonna's dissociation?
Offer grounding strategies
Stop exam

## Slide 32
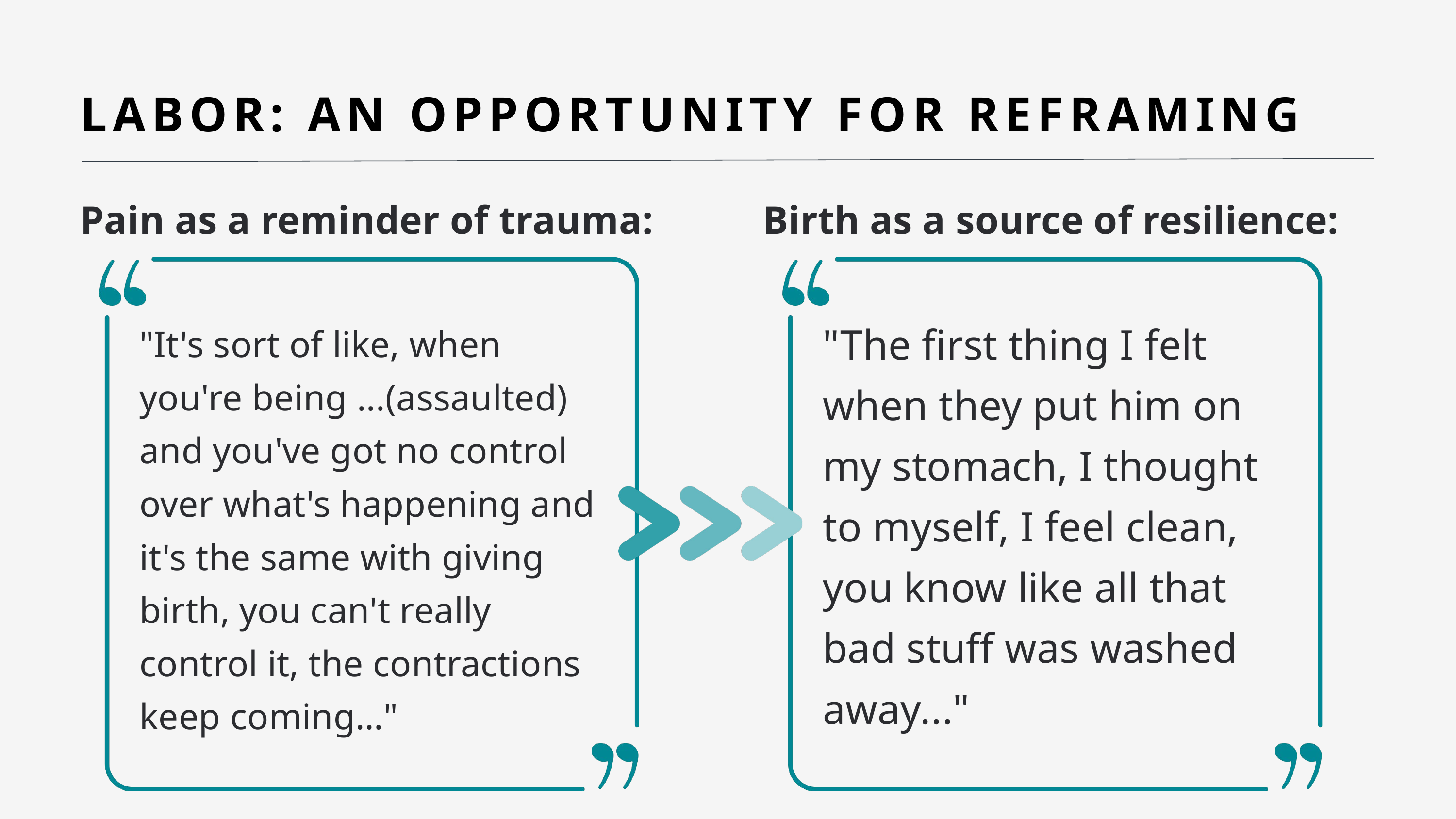

LABOR: AN OPPORTUNITY FOR REFRAMING
Pain as a reminder of trauma:
Birth as a source of resilience:
"The first thing I felt when they put him on my stomach, I thought to myself, I feel clean, you know like all that bad stuff was washed away..."
"It's sort of like, when you're being ...(assaulted) and you've got no control over what's happening and it's the same with giving birth, you can't really control it, the contractions keep coming…"

## Slide 33
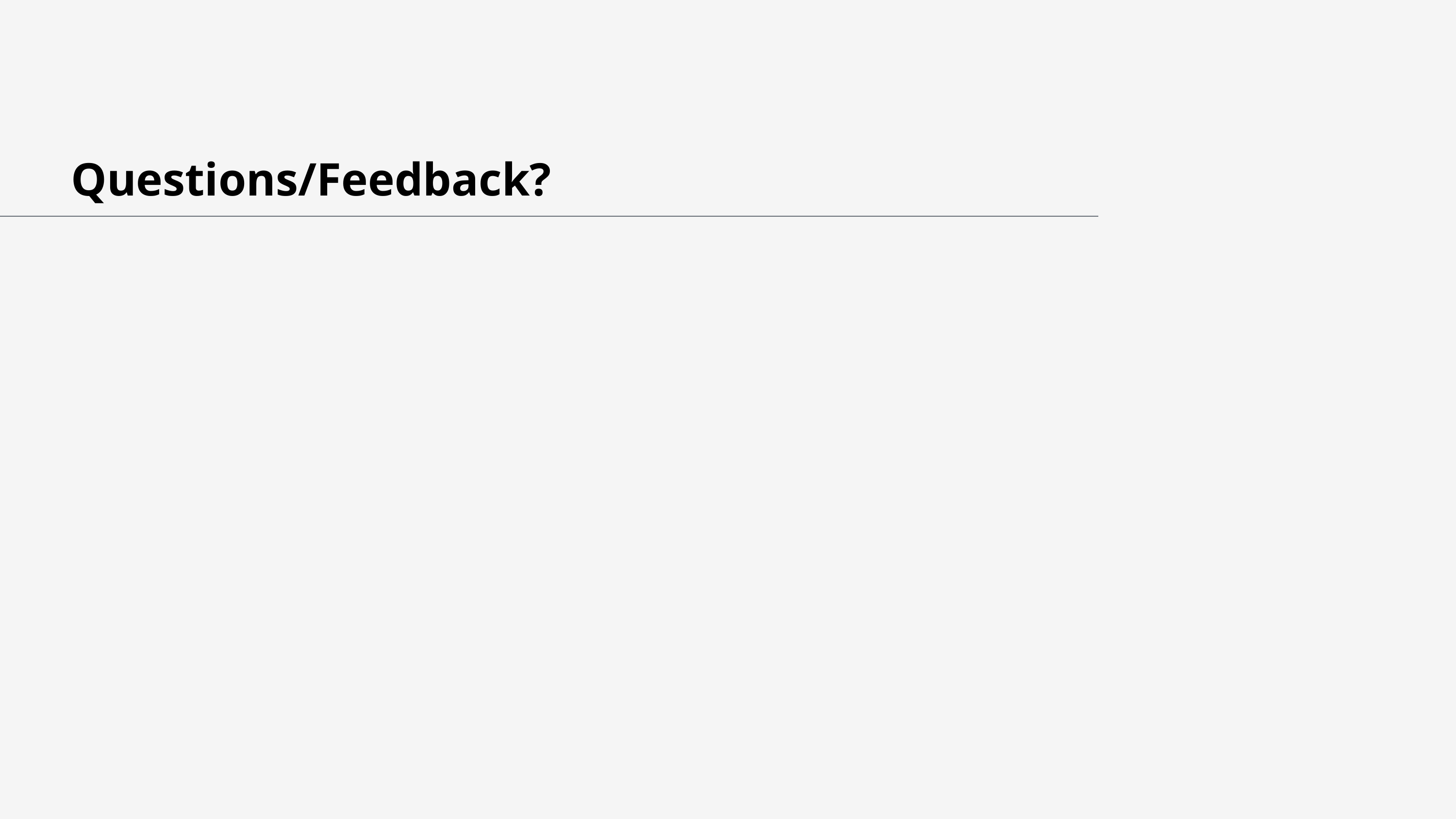

Questions/Feedback?

## Slide 34
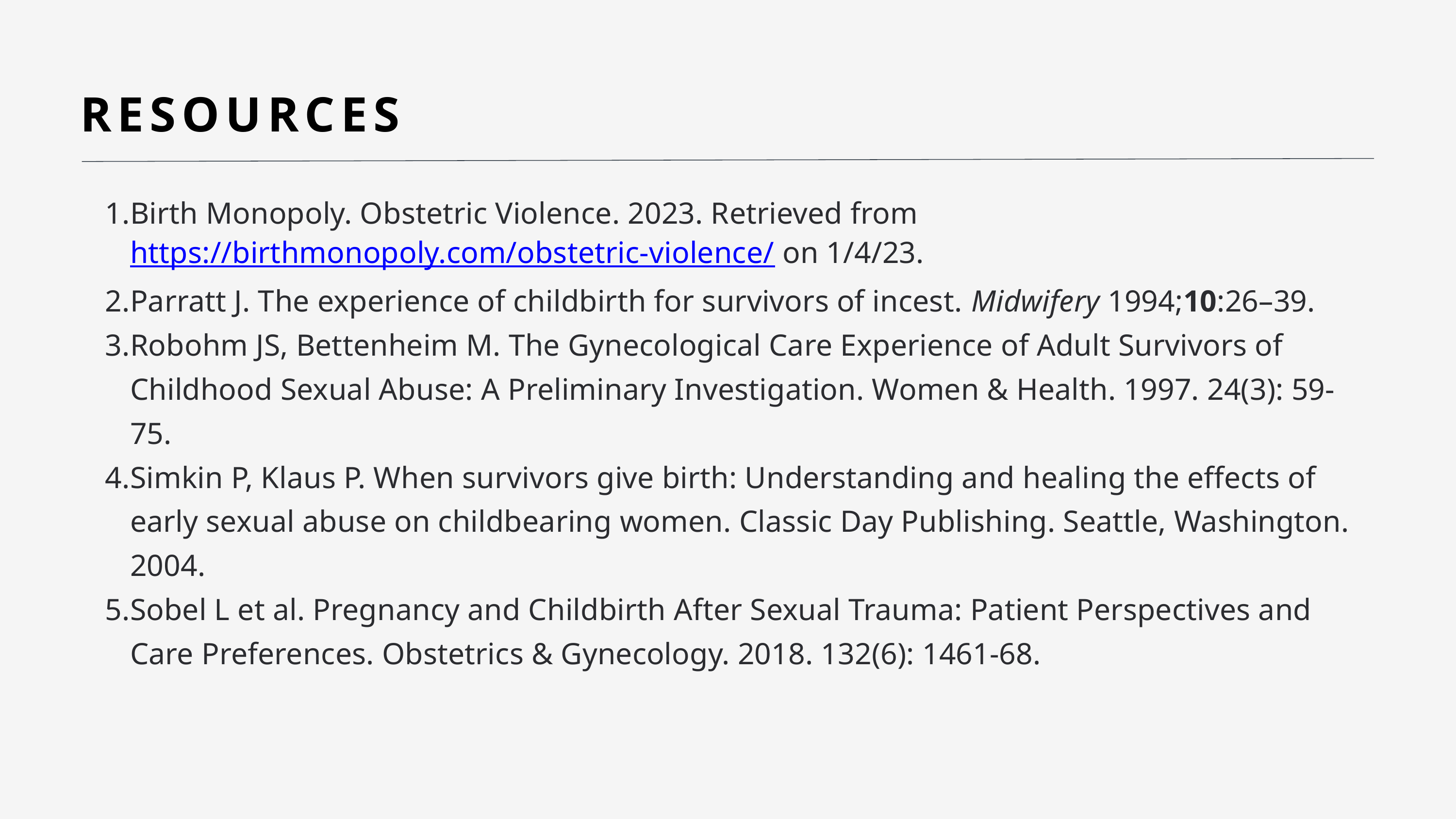

RESOURCES
Birth Monopoly. Obstetric Violence. 2023. Retrieved from https://birthmonopoly.com/obstetric-violence/ on 1/4/23.
Parratt J. The experience of childbirth for survivors of incest. Midwifery 1994;10:26–39.
Robohm JS, Bettenheim M. The Gynecological Care Experience of Adult Survivors of Childhood Sexual Abuse: A Preliminary Investigation. Women & Health. 1997. 24(3): 59-75.
Simkin P, Klaus P. When survivors give birth: Understanding and healing the effects of early sexual abuse on childbearing women. Classic Day Publishing. Seattle, Washington. 2004.
Sobel L et al. Pregnancy and Childbirth After Sexual Trauma: Patient Perspectives and Care Preferences. Obstetrics & Gynecology. 2018. 132(6): 1461-68.
