# Supplementary material for: A Trauma-informed Care Curriculum for Perinatal Providers, Staff, and Learners
Source: MedEdPORTAL. 2025 Dec 9;21:11563. doi: 10.15766/mep_2374-8265.11563 (PMC12686155; doi:10.15766/mep_2374-8265.11563)
Supplement: Supplementary file 1 — Part 1 - Overview of TIC.pptxPart 2 - TIC in Perinatal Care.pptxPart 3 - Vicarious Trauma.pptxPart 4 - Community Voices & Reflection.pptxPresurvey.docxPostsurvey.pdf [file mep_2374-8265.11563-s001.zip › C. Part 3 - Vicarious Trauma.pptx]

## Slide 1
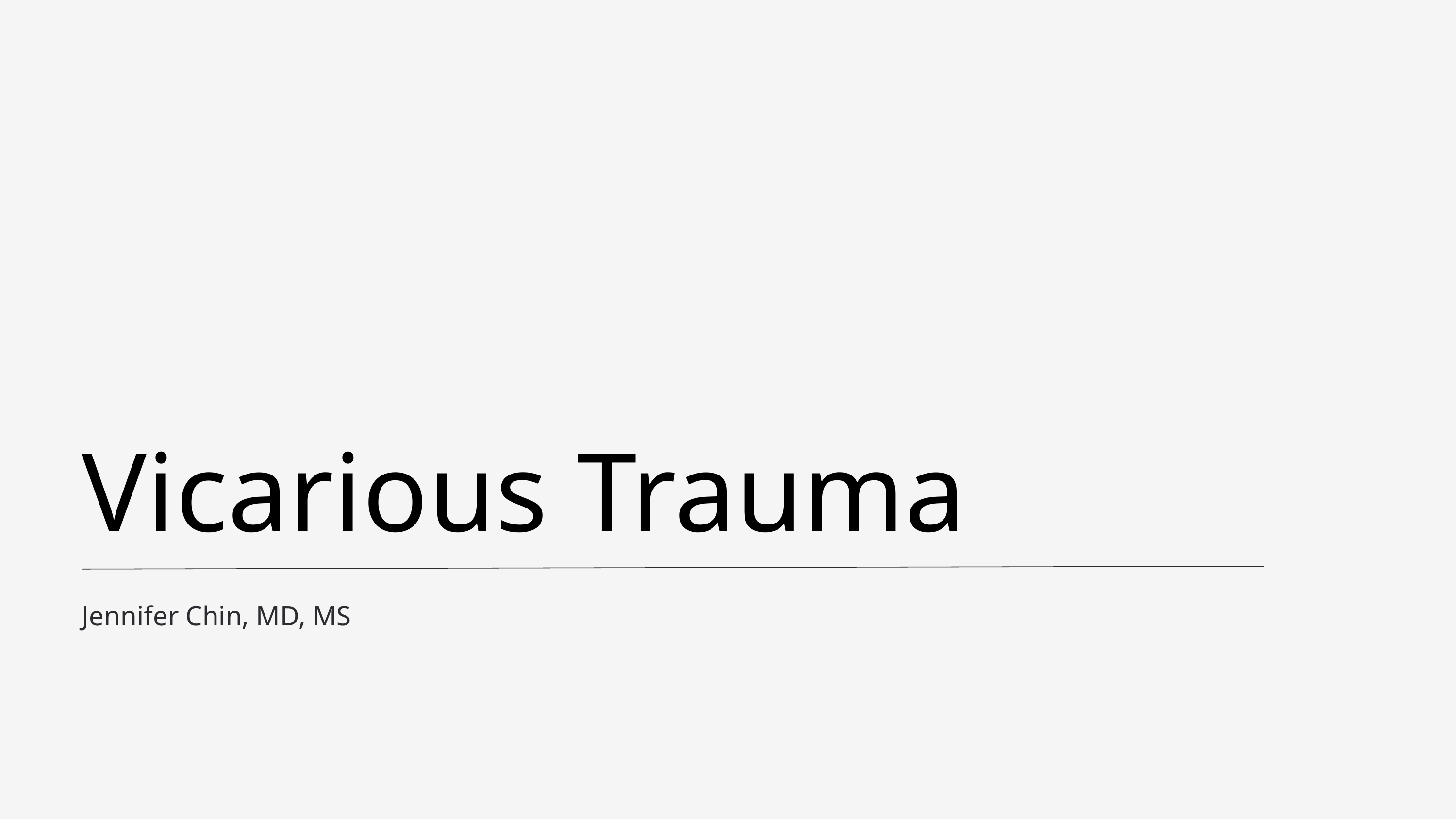

Vicarious Trauma
Jennifer Chin, MD, MS

## Slide 2
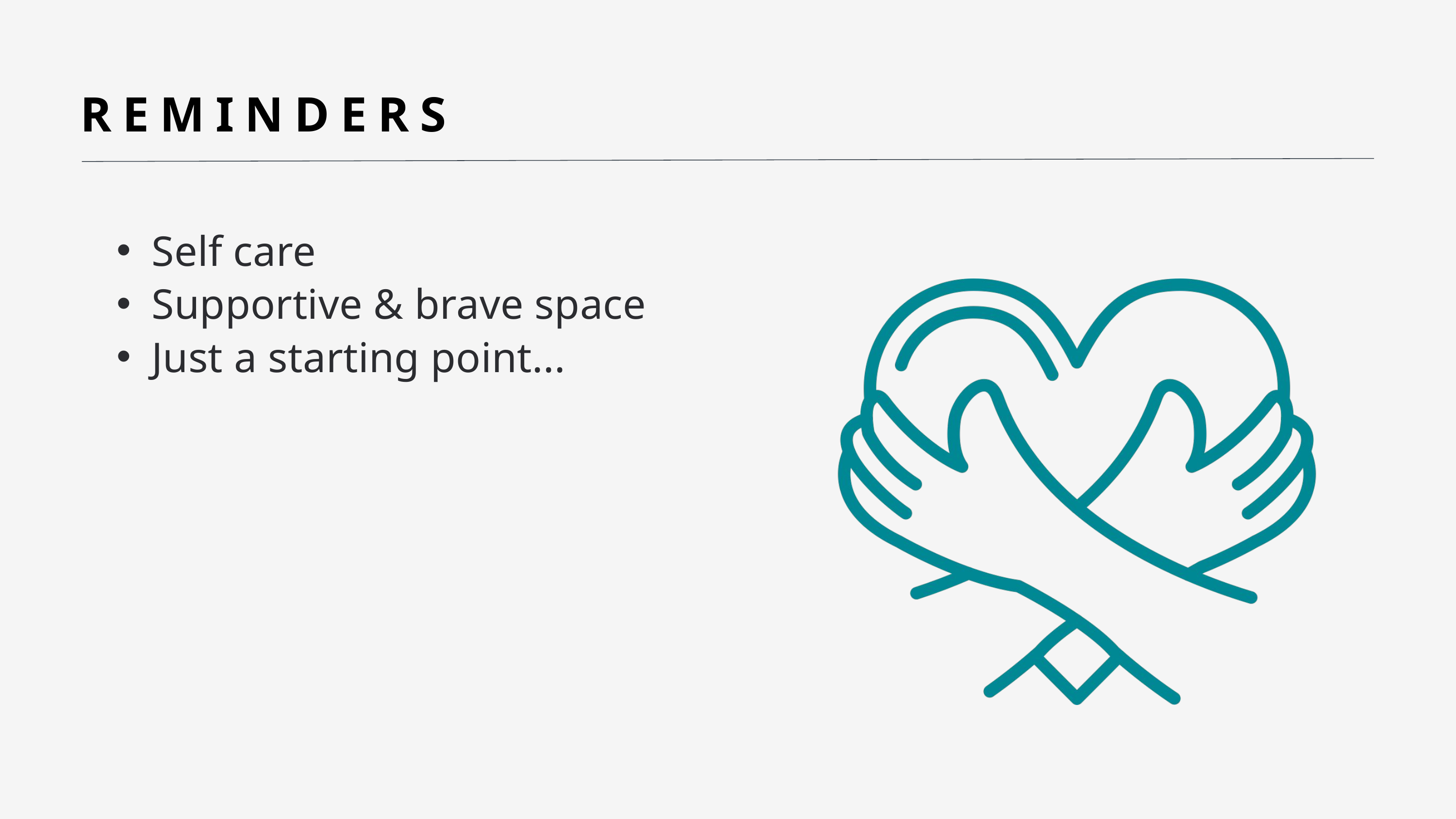

REMINDERS
Self care
Supportive & brave space
Just a starting point...

## Slide 3
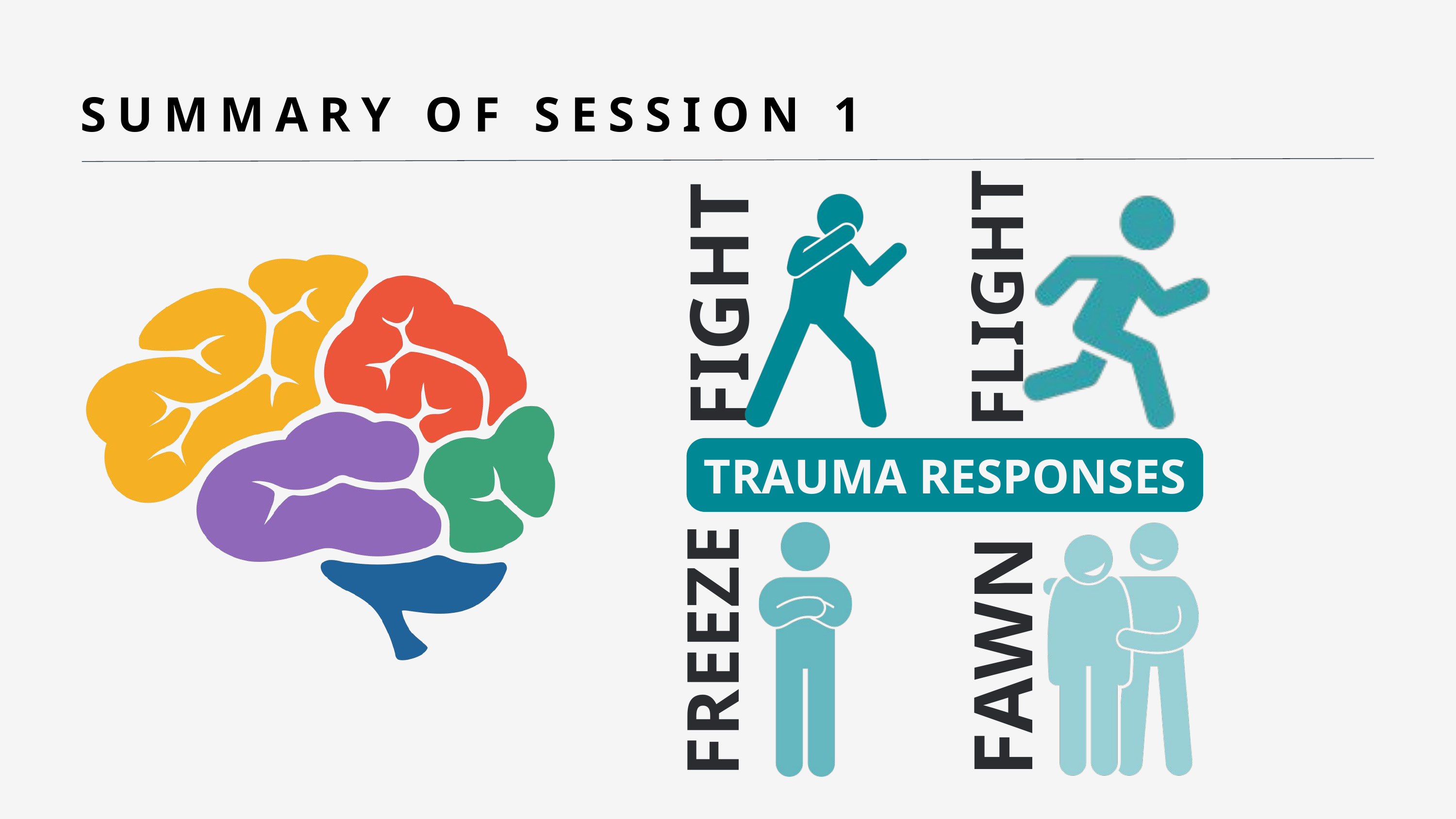

SUMMARY OF SESSION 1
FLIGHT
FIGHT
TRAUMA RESPONSES
FAWN
FREEZE

## Slide 4
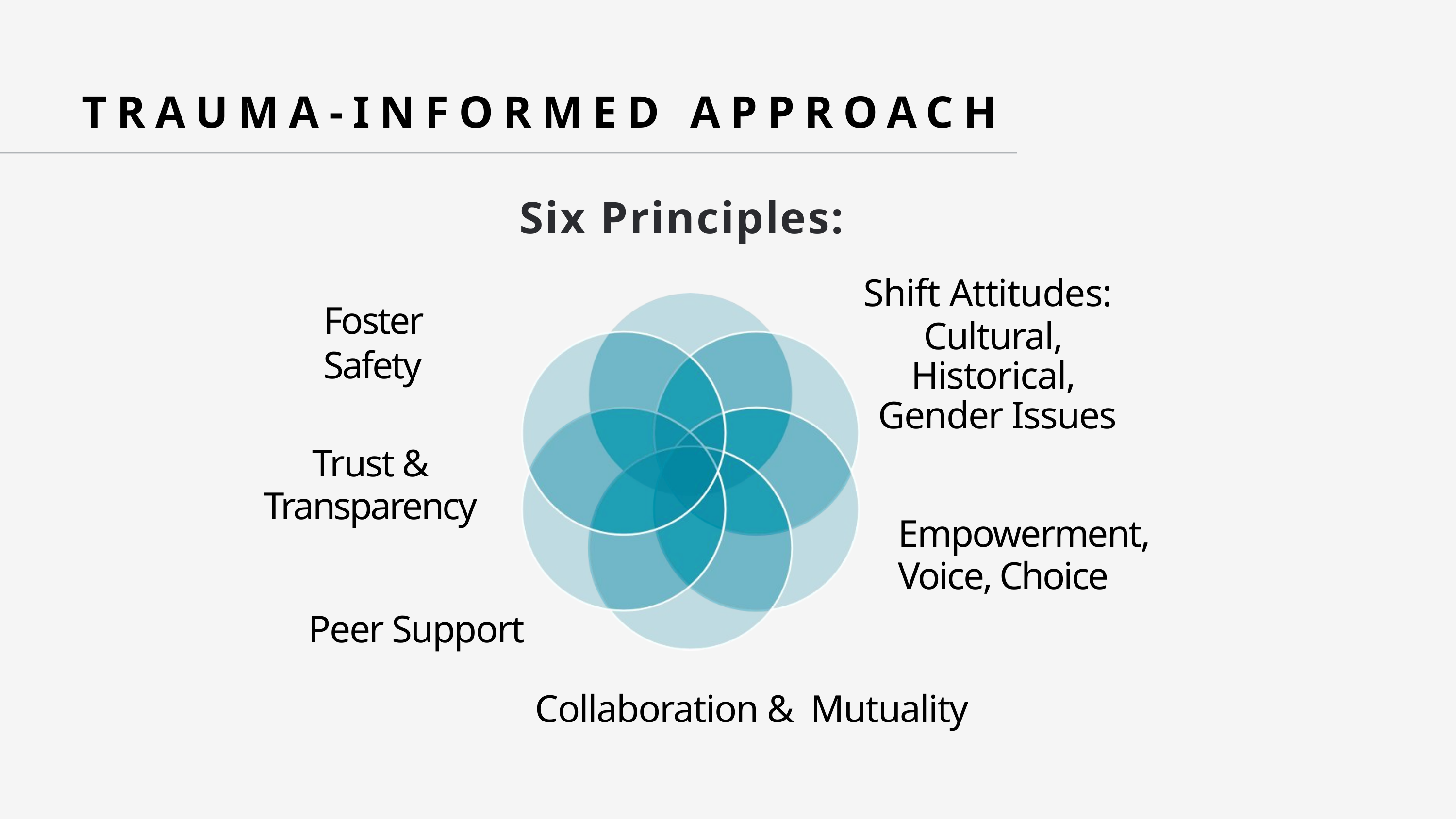

TRAUMA-INFORMED APPROACH
Six Principles:
Shift Attitudes:
Foster Safety
Cultural, Historical, Gender Issues
Trust &
Transparency
Empowerment,
Voice, Choice
Peer Support
Collaboration & Mutuality

## Slide 5
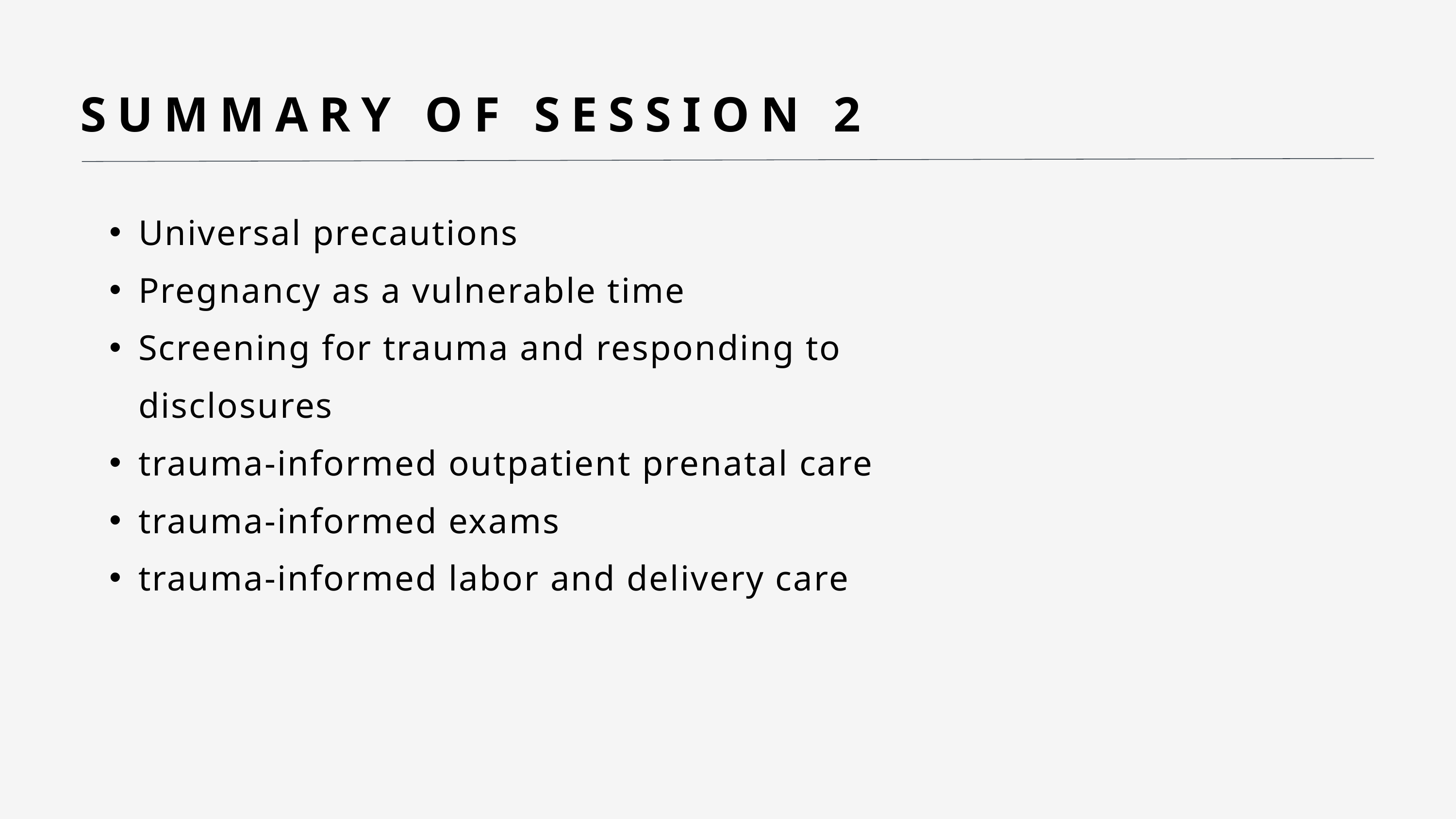

SUMMARY OF SESSION 2
Universal precautions
Pregnancy as a vulnerable time
Screening for trauma and responding to disclosures
trauma-informed outpatient prenatal care
trauma-informed exams
trauma-informed labor and delivery care

## Slide 6
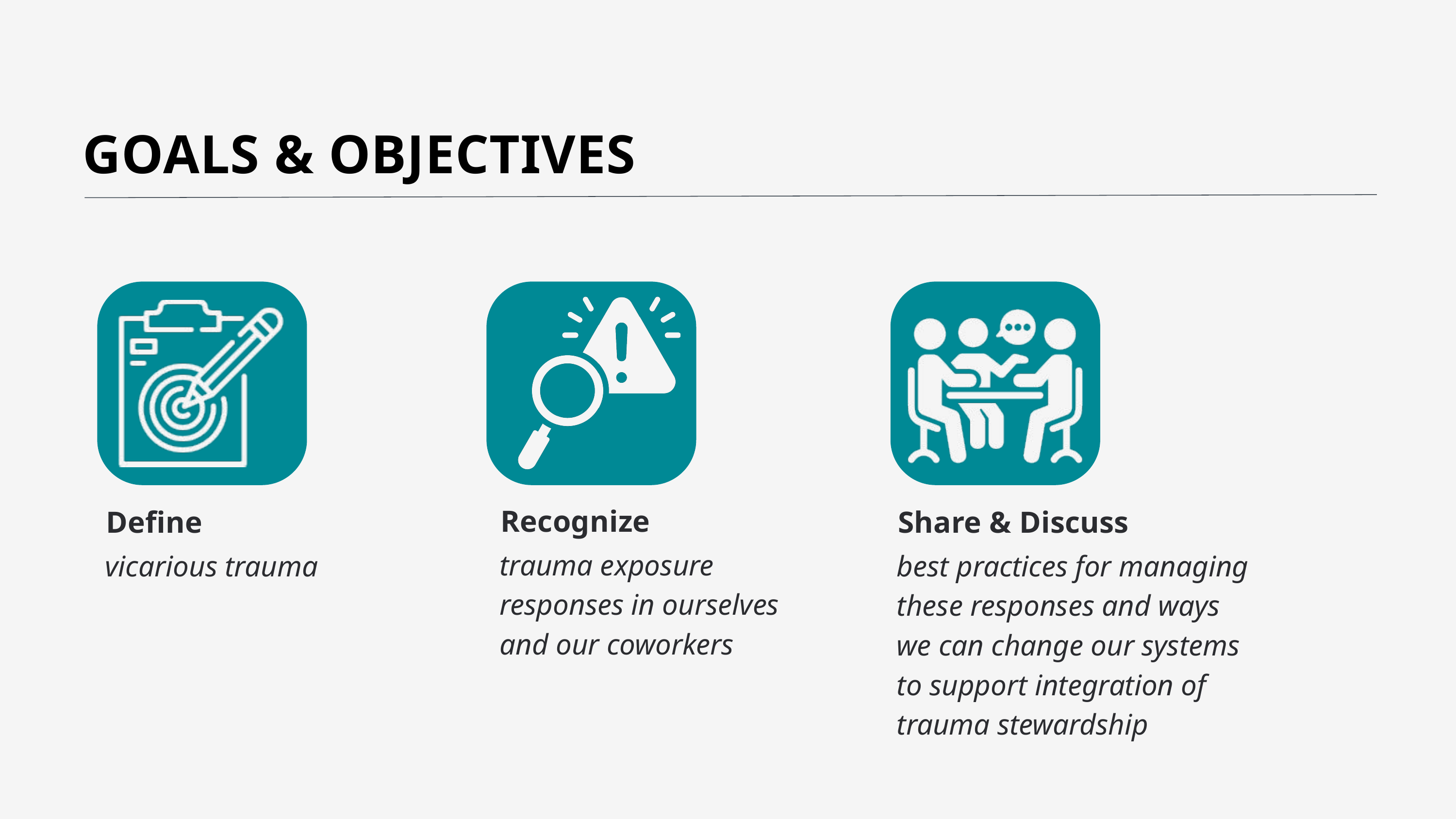

GOALS & OBJECTIVES
Recognize
Define
Share & Discuss
trauma exposure responses in ourselves and our coworkers
vicarious trauma
best practices for managing these responses and ways we can change our systems to support integration of trauma stewardship

## Slide 7
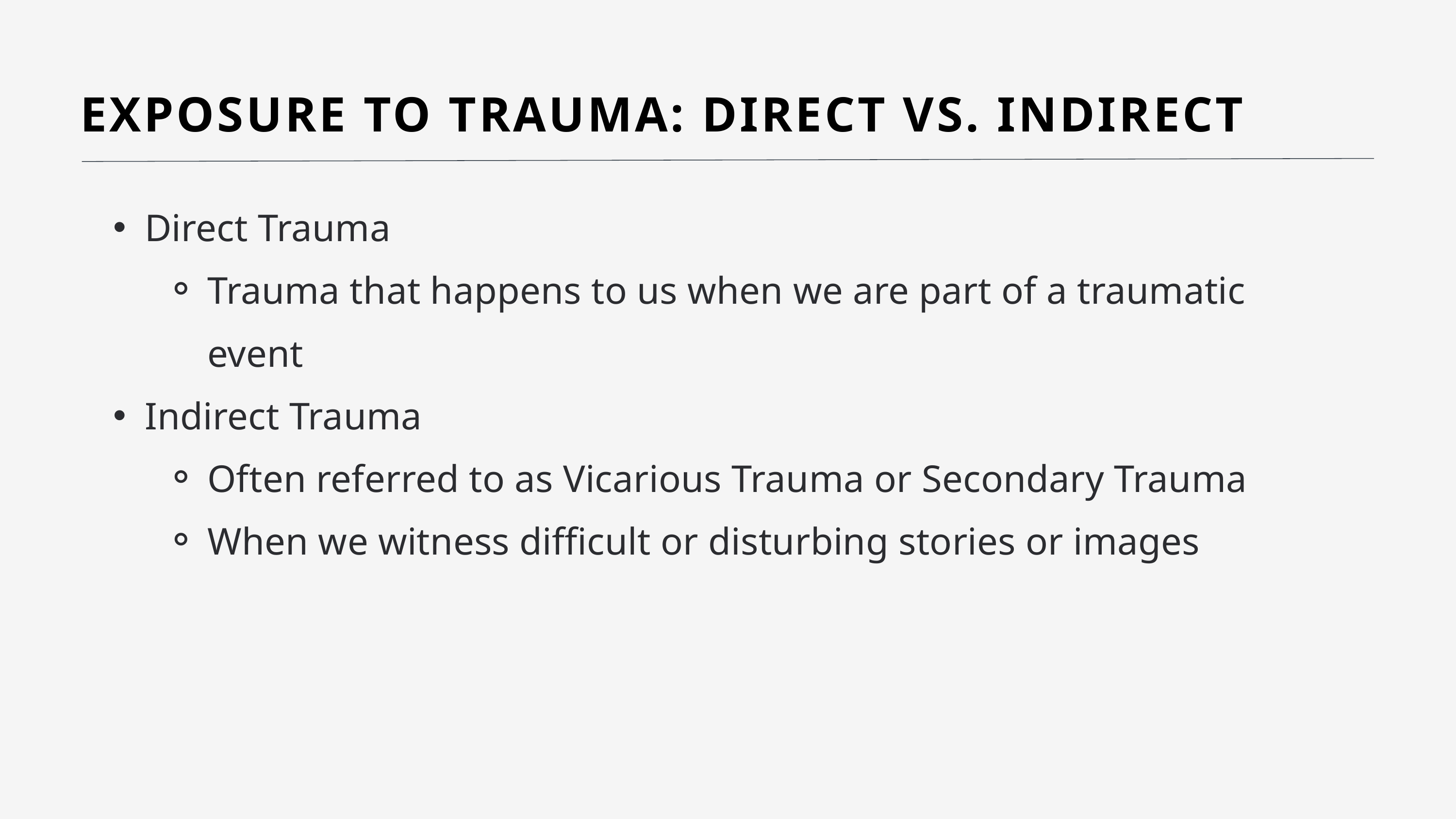

EXPOSURE TO TRAUMA: DIRECT VS. INDIRECT
Direct Trauma
Trauma that happens to us when we are part of a traumatic event
Indirect Trauma
Often referred to as Vicarious Trauma or Secondary Trauma
When we witness difficult or disturbing stories or images

## Slide 8
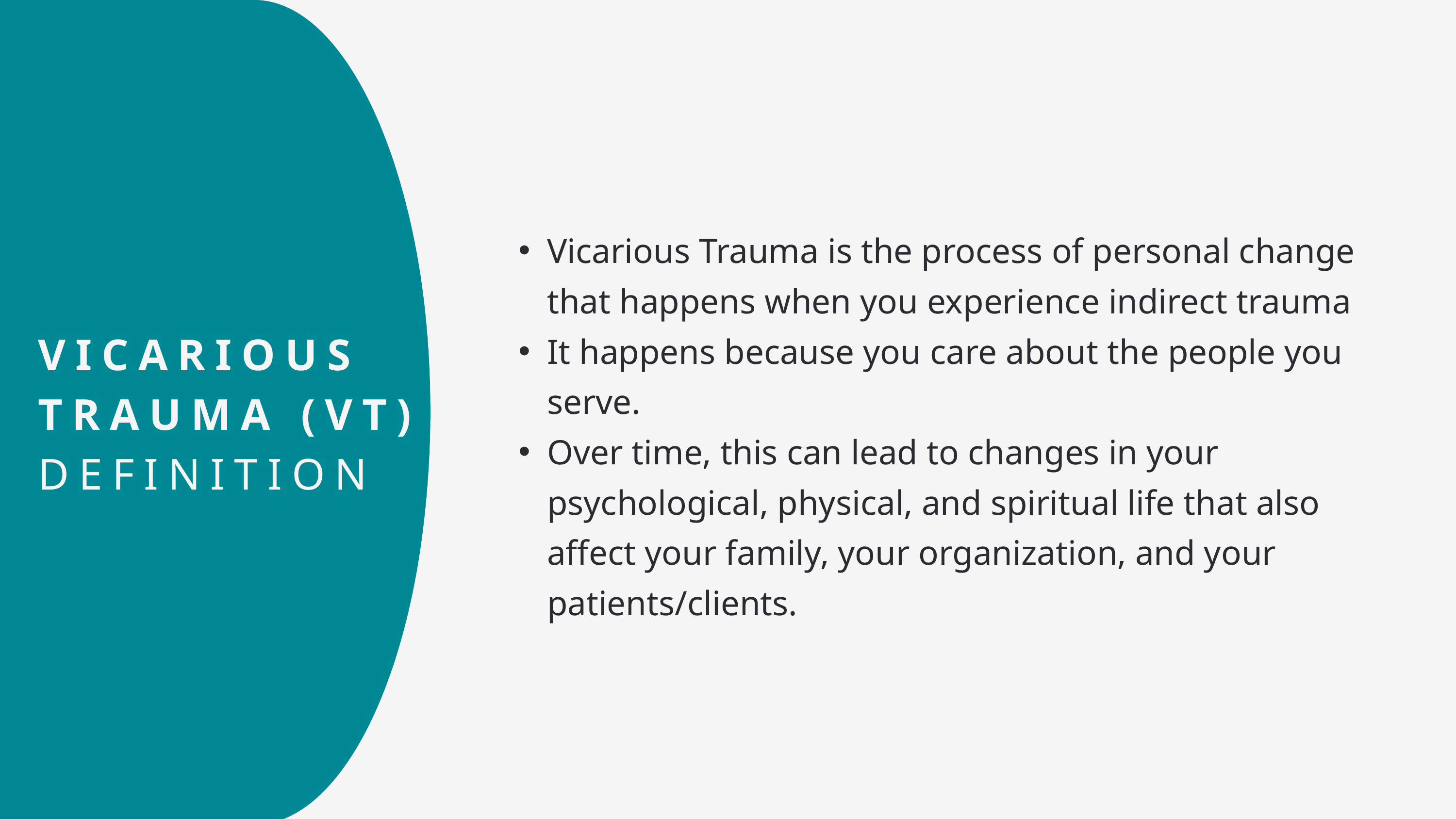

Vicarious Trauma is the process of personal change that happens when you experience indirect trauma
It happens because you care about the people you serve.
Over time, this can lead to changes in your psychological, physical, and spiritual life that also affect your family, your organization, and your patients/clients.
VICARIOUS TRAUMA (VT)
DEFINITION

## Slide 9
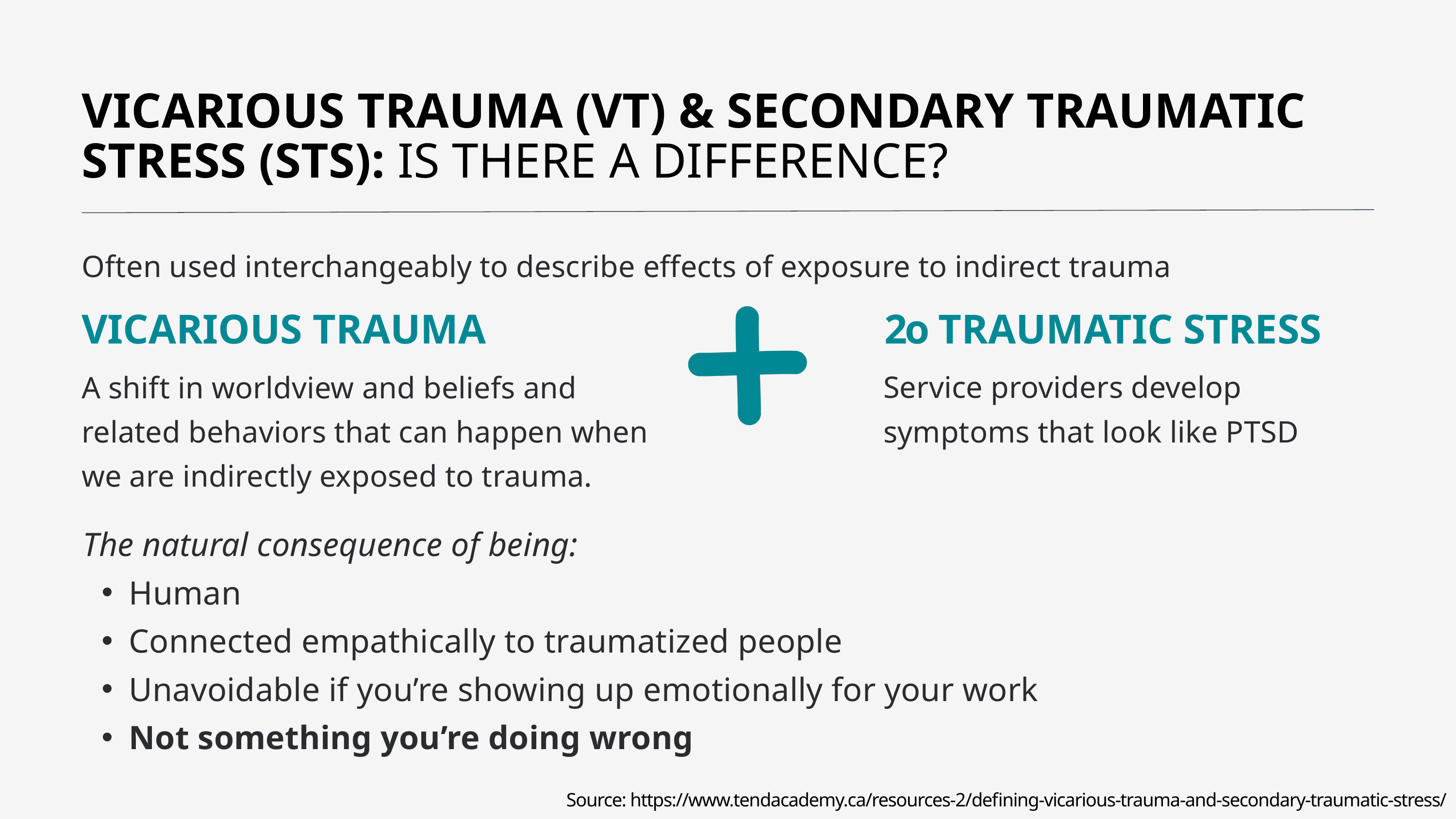

VICARIOUS TRAUMA (VT) & SECONDARY TRAUMATIC STRESS (STS): IS THERE A DIFFERENCE?
Often used interchangeably to describe effects of exposure to indirect trauma
VICARIOUS TRAUMA
2 TRAUMATIC STRESS
o
Service providers develop symptoms that look like PTSD
A shift in worldview and beliefs and related behaviors that can happen when we are indirectly exposed to trauma.
 The natural consequence of being:
Human
Connected empathically to traumatized people
Unavoidable if you’re showing up emotionally for your work
Not something you’re doing wrong
Source: https://www.tendacademy.ca/resources-2/defining-vicarious-trauma-and-secondary-traumatic-stress/

## Slide 10
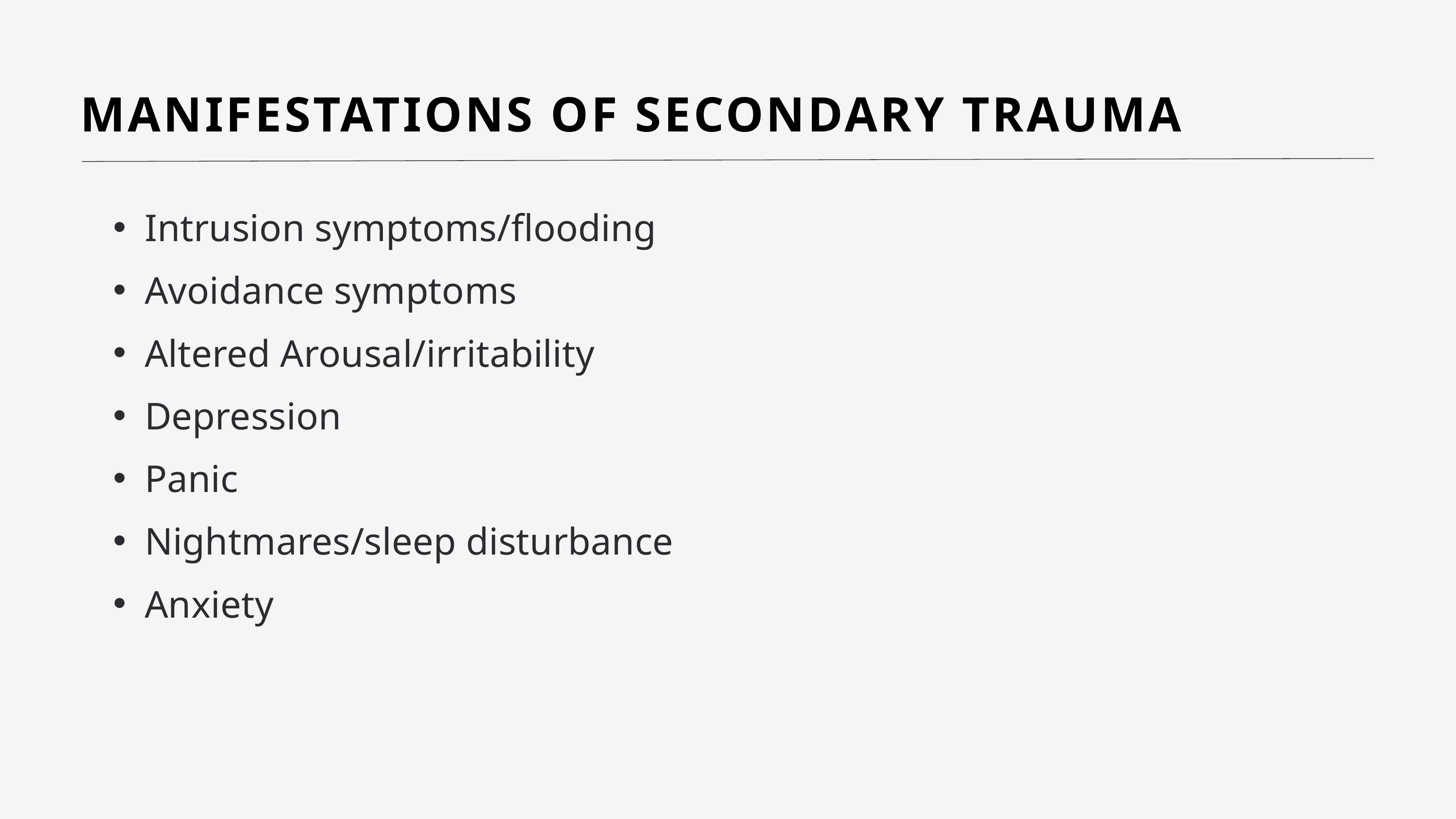

MANIFESTATIONS OF SECONDARY TRAUMA
Intrusion symptoms/flooding
Avoidance symptoms
Altered Arousal/irritability
Depression
Panic
Nightmares/sleep disturbance
Anxiety

## Slide 11
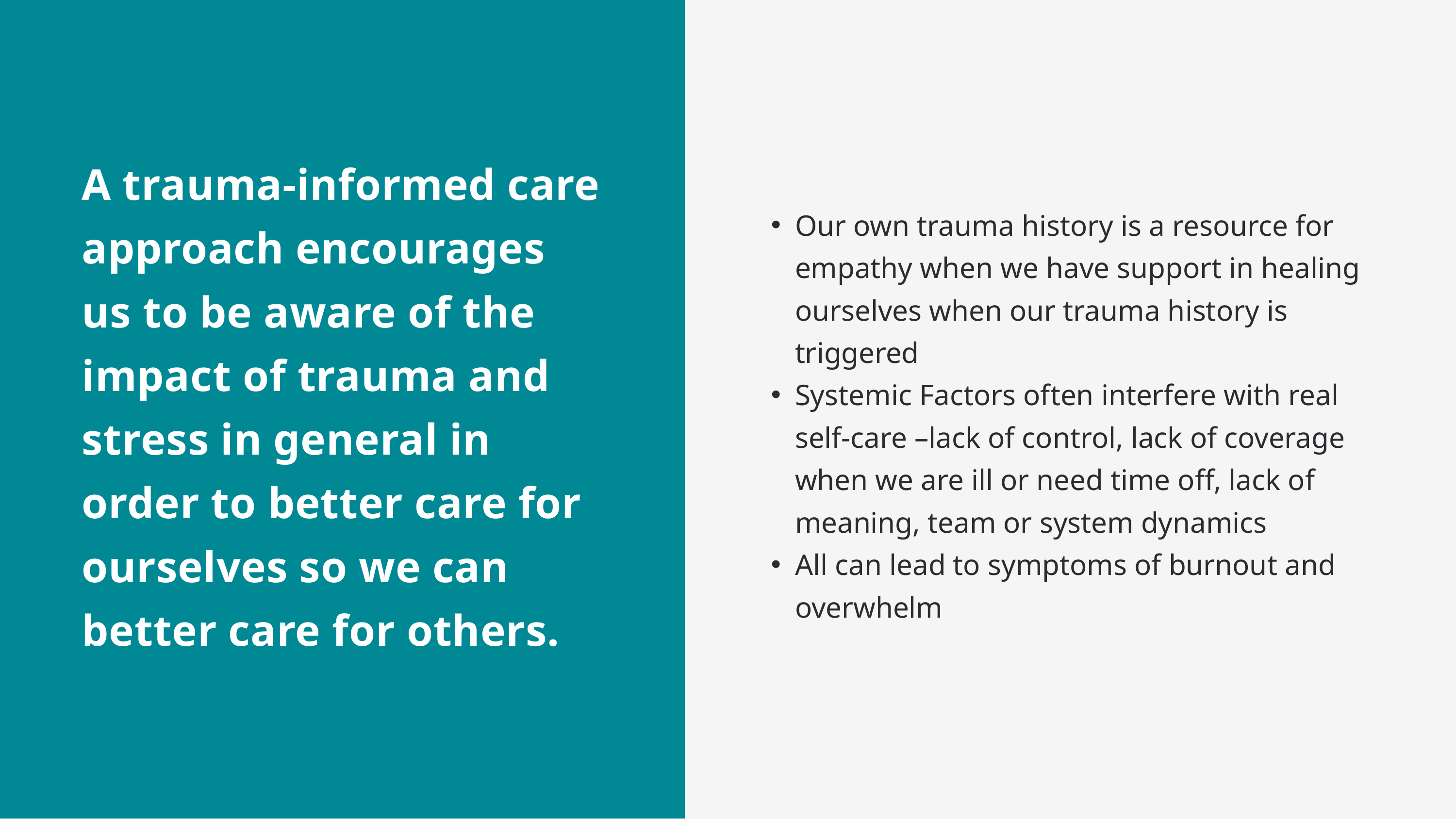

A trauma-informed care approach encourages us to be aware of the impact of trauma and stress in general in order to better care for ourselves so we can better care for others.
Our own trauma history is a resource for empathy when we have support in healing ourselves when our trauma history is triggered
Systemic Factors often interfere with real self-care –lack of control, lack of coverage when we are ill or need time off, lack of meaning, team or system dynamics
All can lead to symptoms of burnout and overwhelm

## Slide 12
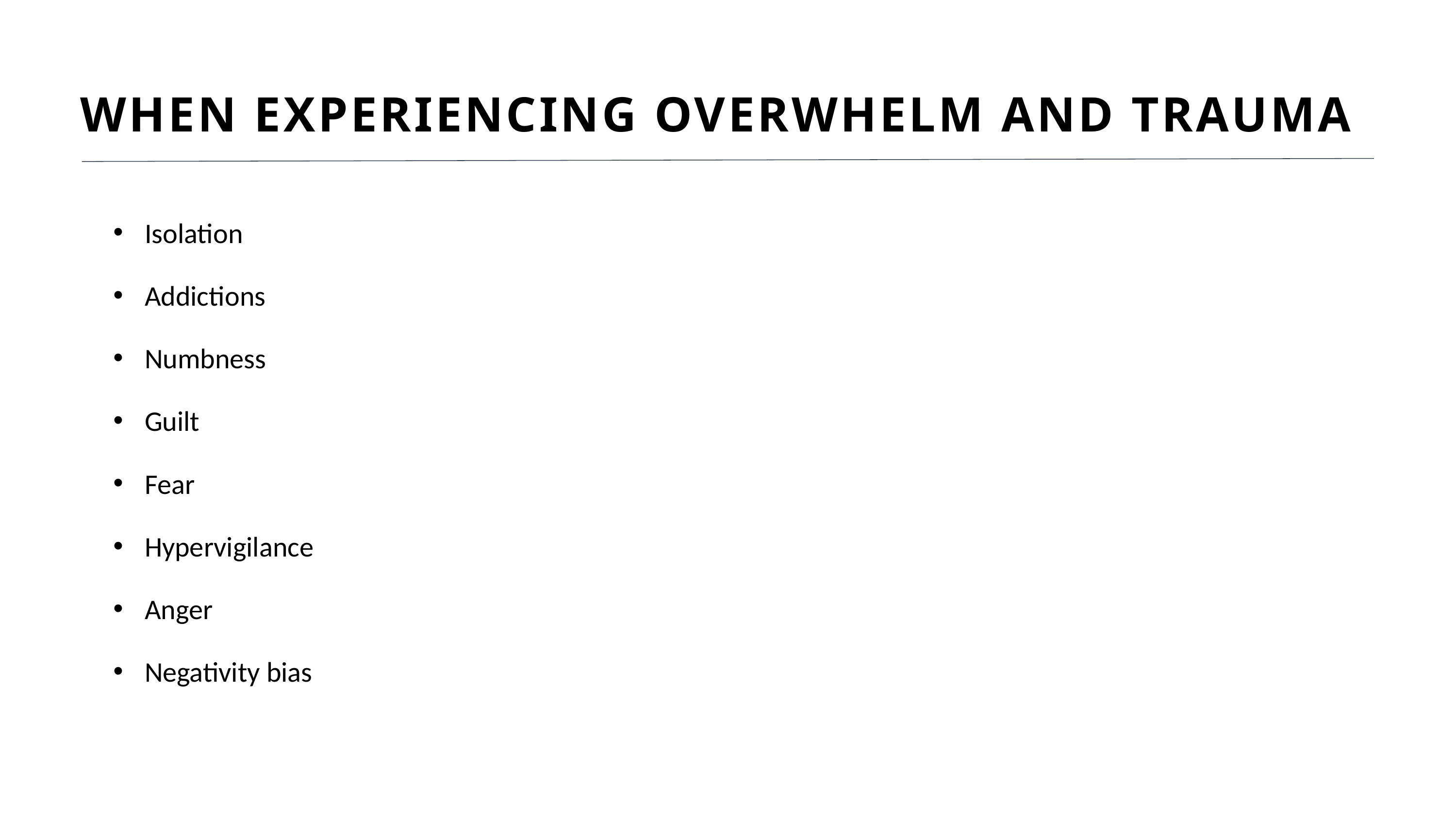

WHEN EXPERIENCING OVERWHELM AND TRAUMA
Isolation
Addictions
Numbness
Guilt
Fear
Hypervigilance
Anger
Negativity bias

## Slide 13
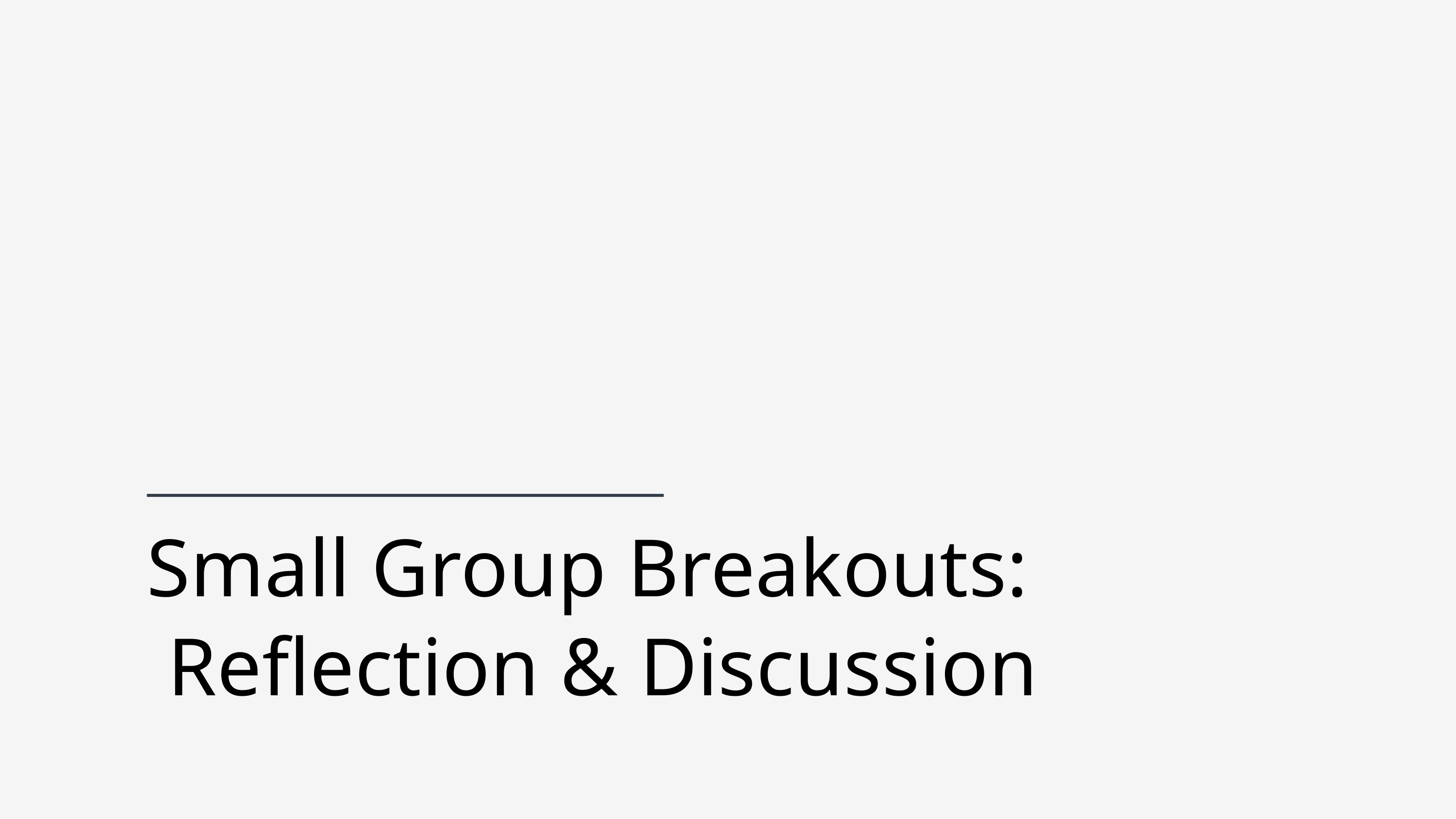

Small Group Breakouts:
 Reflection & Discussion

## Slide 14
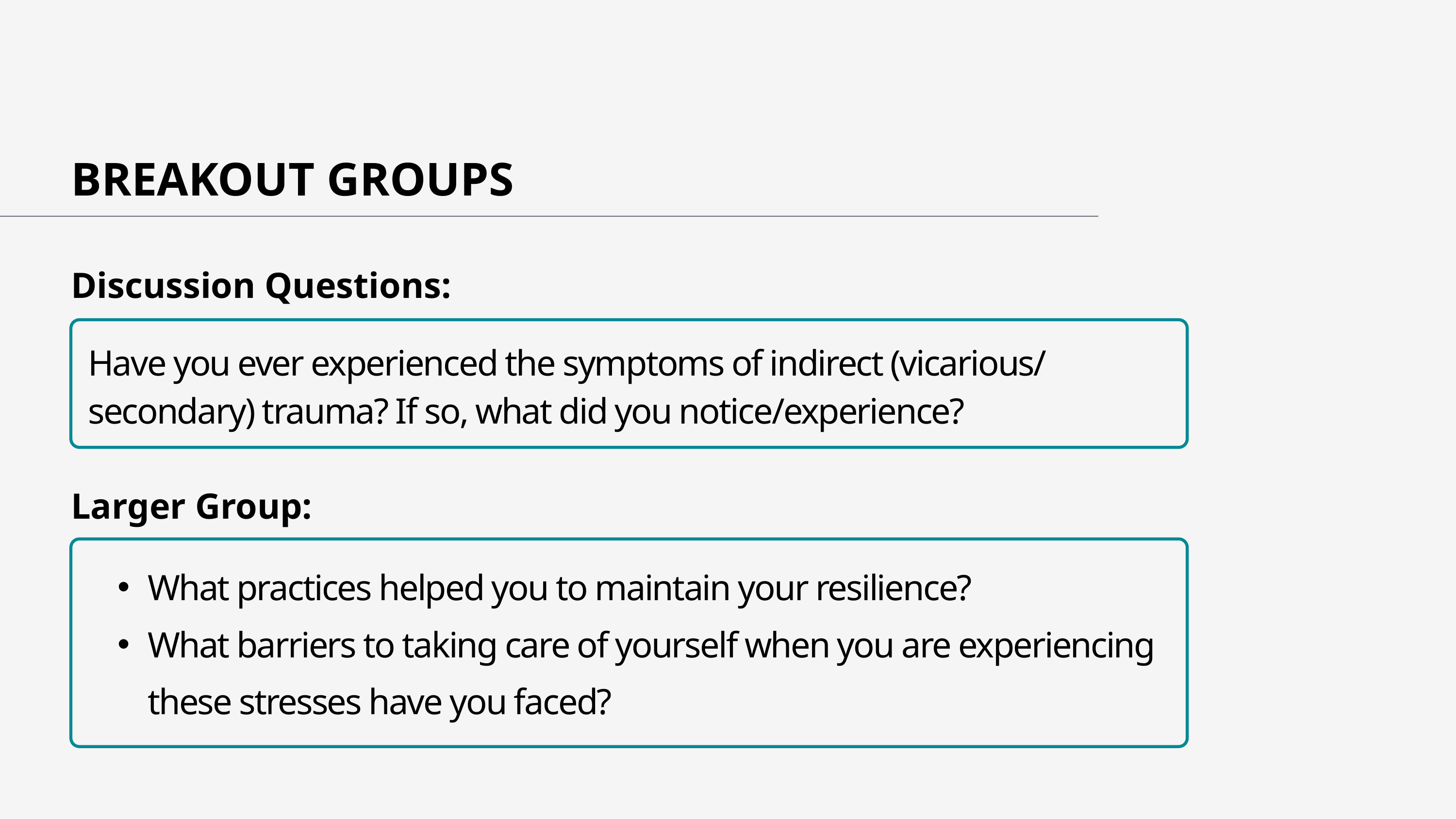

BREAKOUT GROUPS
Discussion Questions:
Have you ever experienced the symptoms of indirect (vicarious/ secondary) trauma? If so, what did you notice/experience?
Larger Group:
What practices helped you to maintain your resilience?
What barriers to taking care of yourself when you are experiencing these stresses have you faced?

## Slide 15
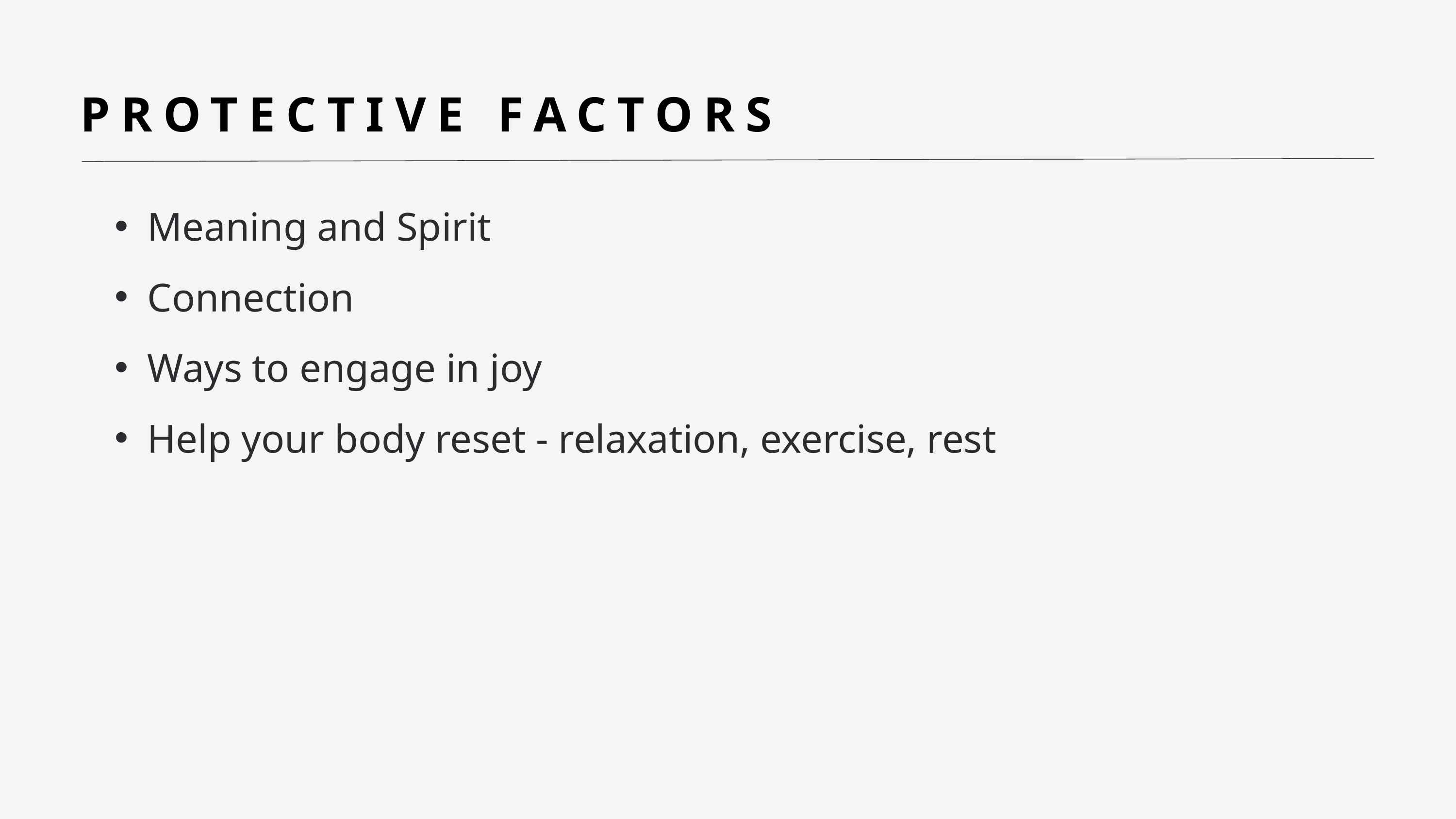

PROTECTIVE FACTORS
Meaning and Spirit
Connection
Ways to engage in joy
Help your body reset - relaxation, exercise, rest

## Slide 16
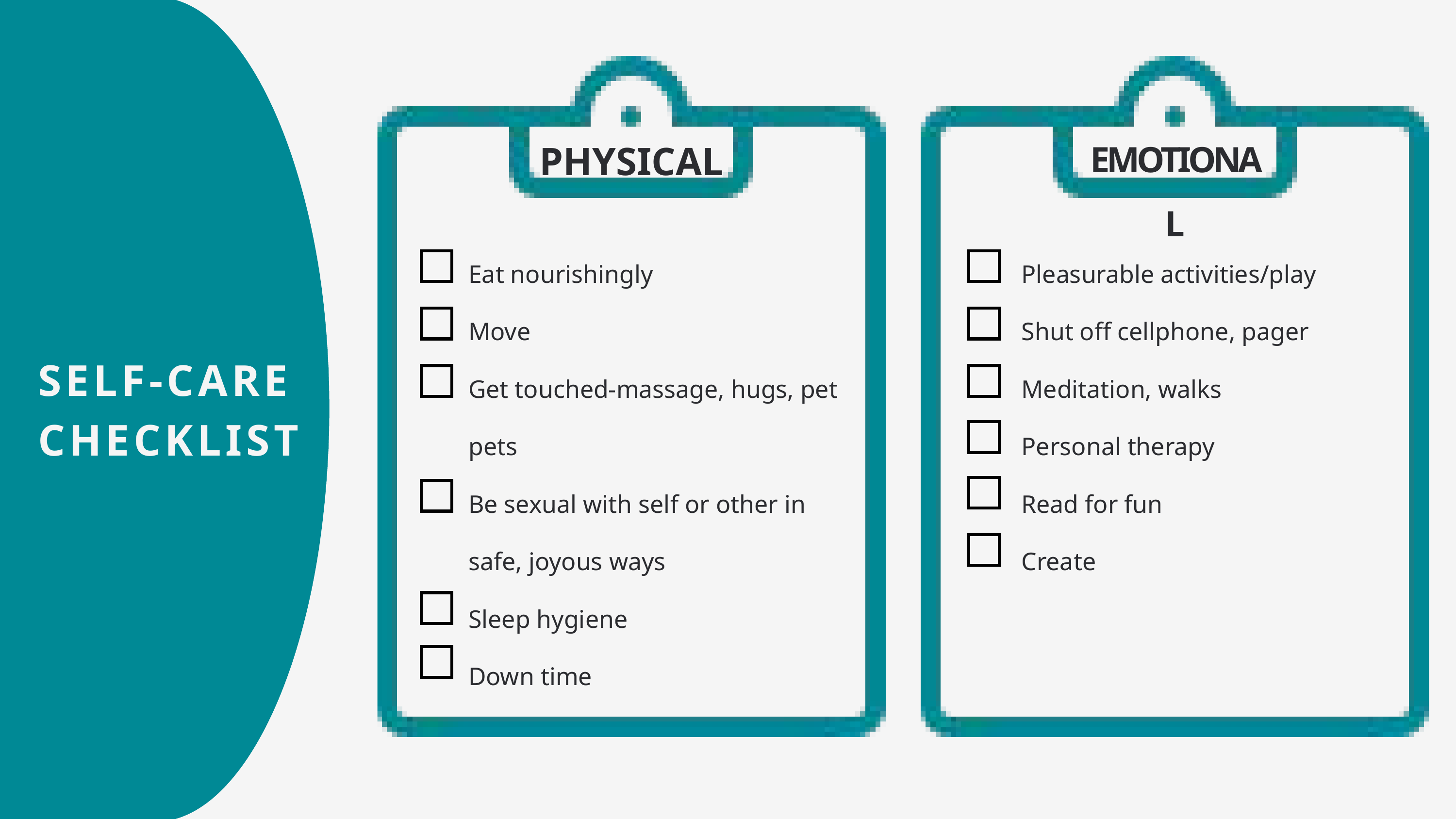

PHYSICAL
EMOTIONAL
Eat nourishingly
Move
Get touched-massage, hugs, pet pets
Be sexual with self or other in safe, joyous ways
Sleep hygiene
Down time
Pleasurable activities/play
Shut off cellphone, pager
Meditation, walks
Personal therapy
Read for fun
Create
SELF-CARE CHECKLIST

## Slide 17
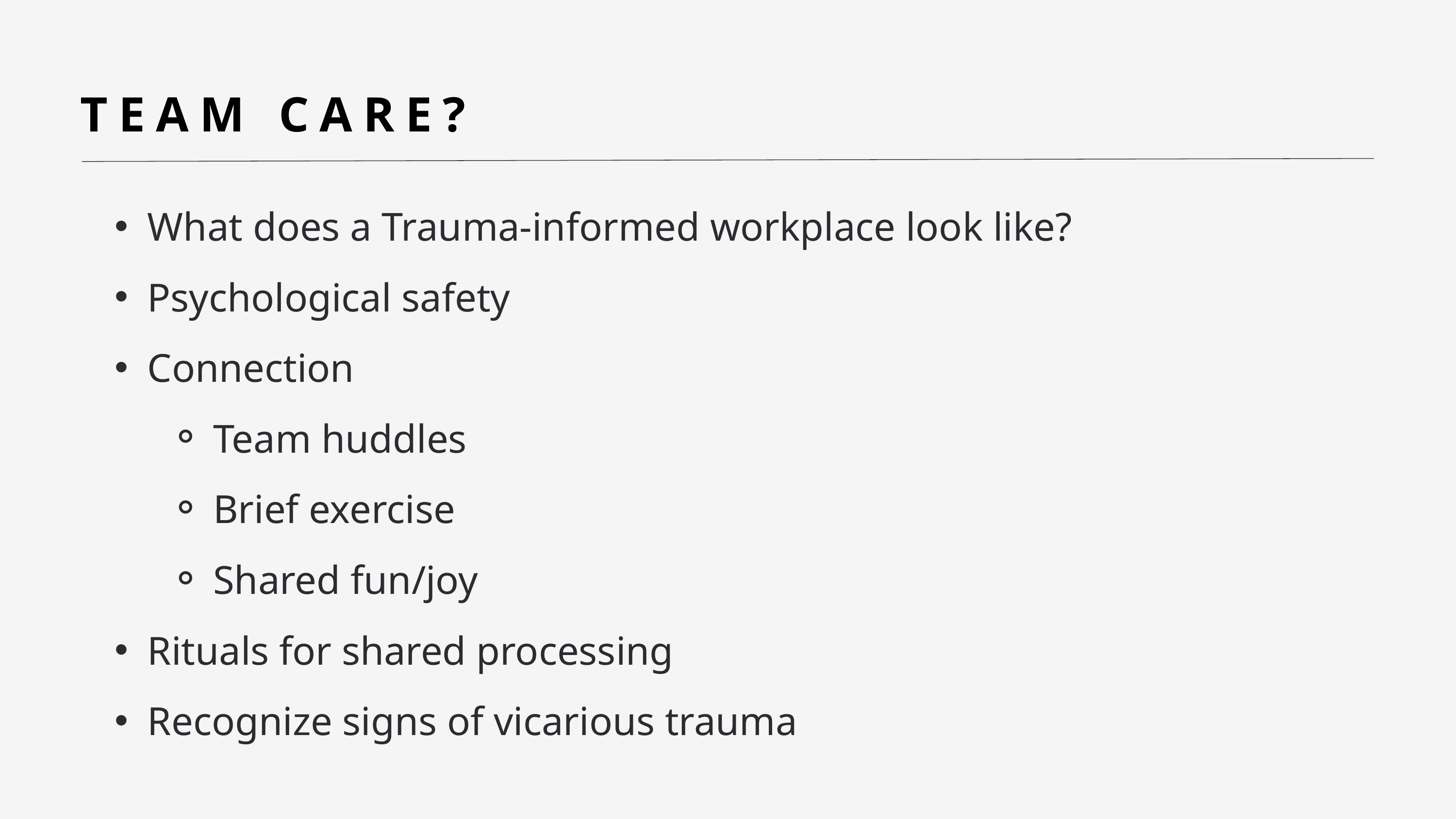

TEAM CARE?
What does a Trauma-informed workplace look like?
Psychological safety
Connection
Team huddles
Brief exercise
Shared fun/joy
Rituals for shared processing
Recognize signs of vicarious trauma

## Slide 18
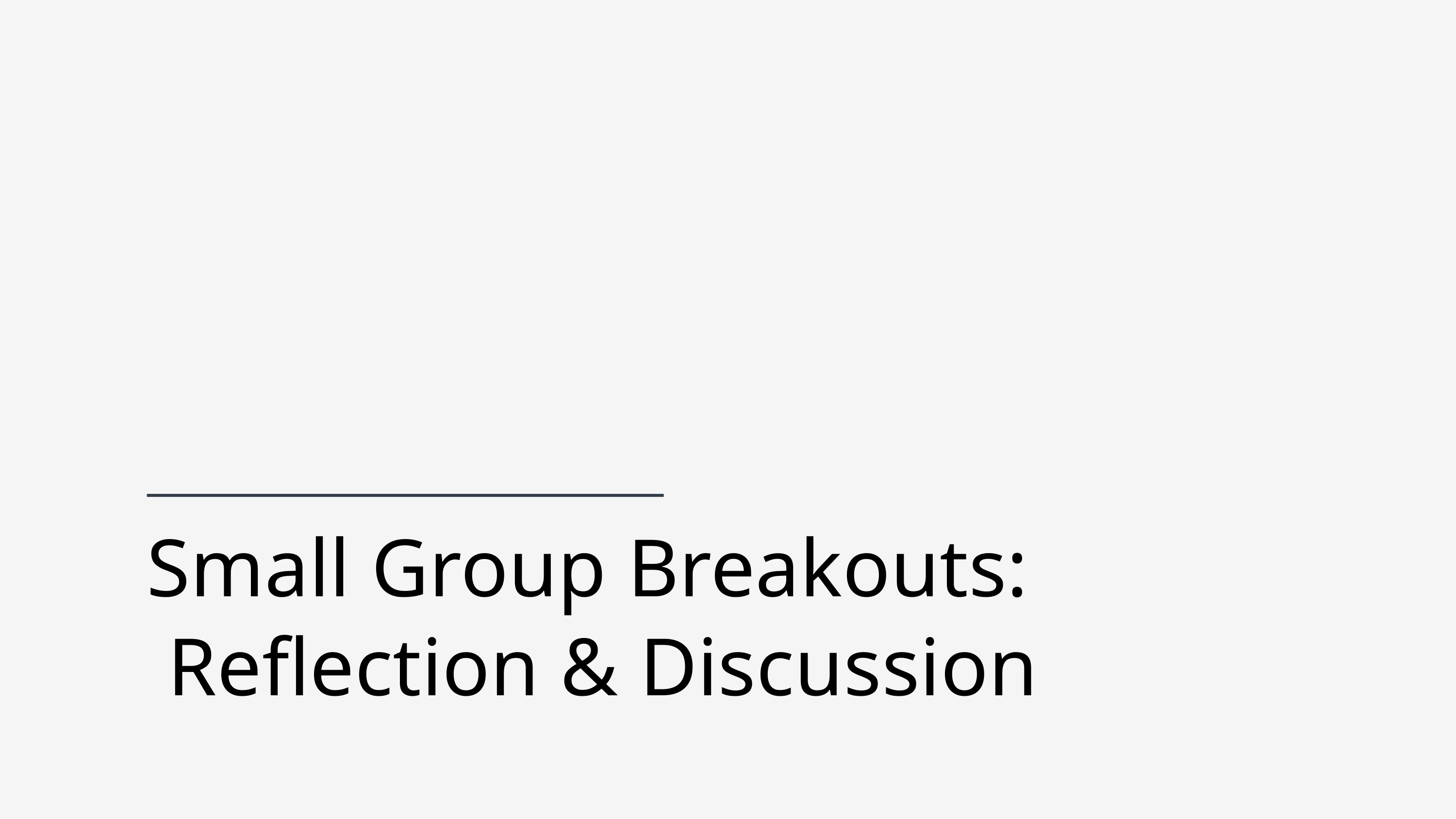

Small Group Breakouts:
 Reflection & Discussion

## Slide 19
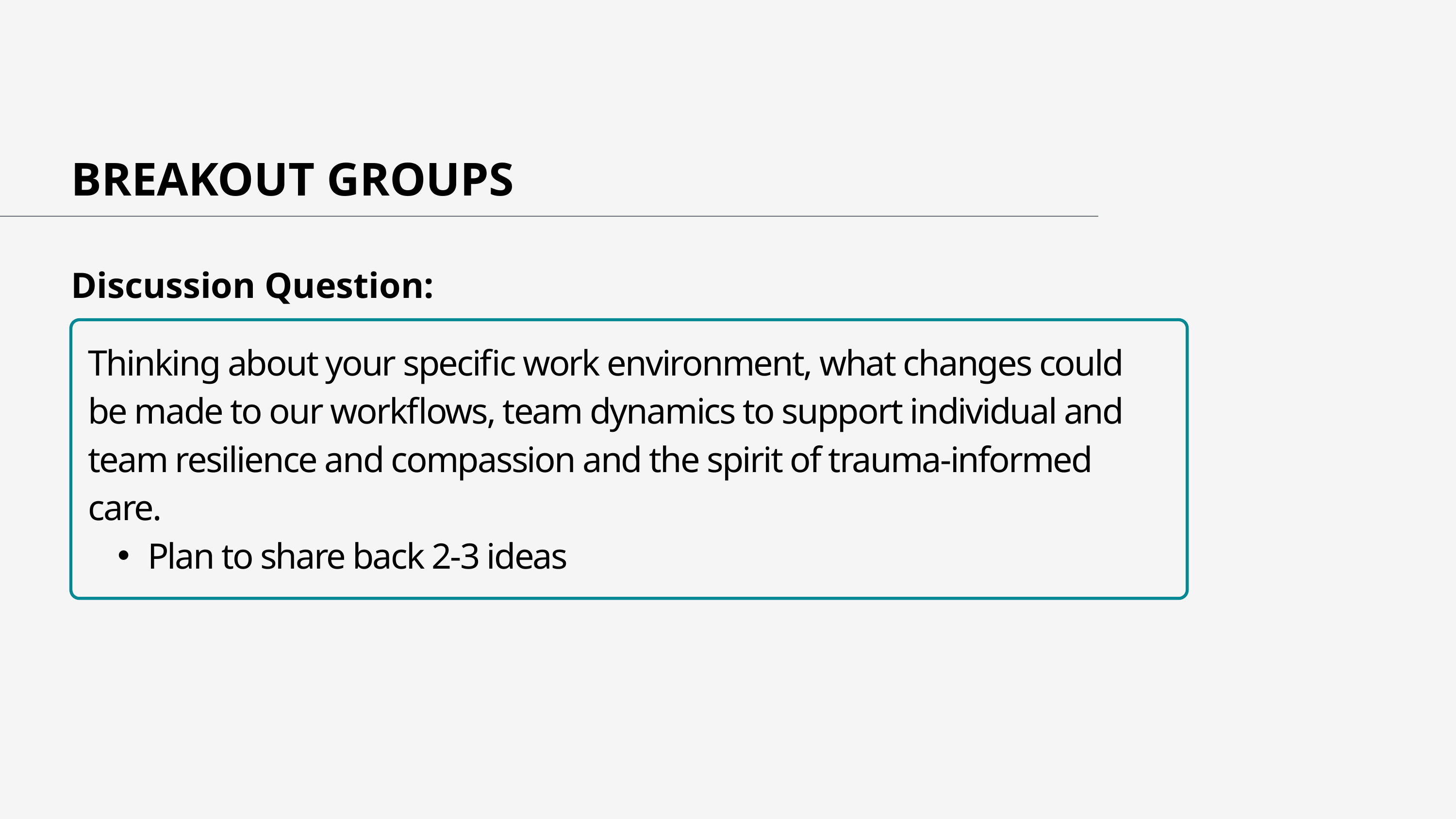

BREAKOUT GROUPS
Discussion Question:
Thinking about your specific work environment, what changes could be made to our workflows, team dynamics to support individual and team resilience and compassion and the spirit of trauma-informed care.
Plan to share back 2-3 ideas

## Slide 20
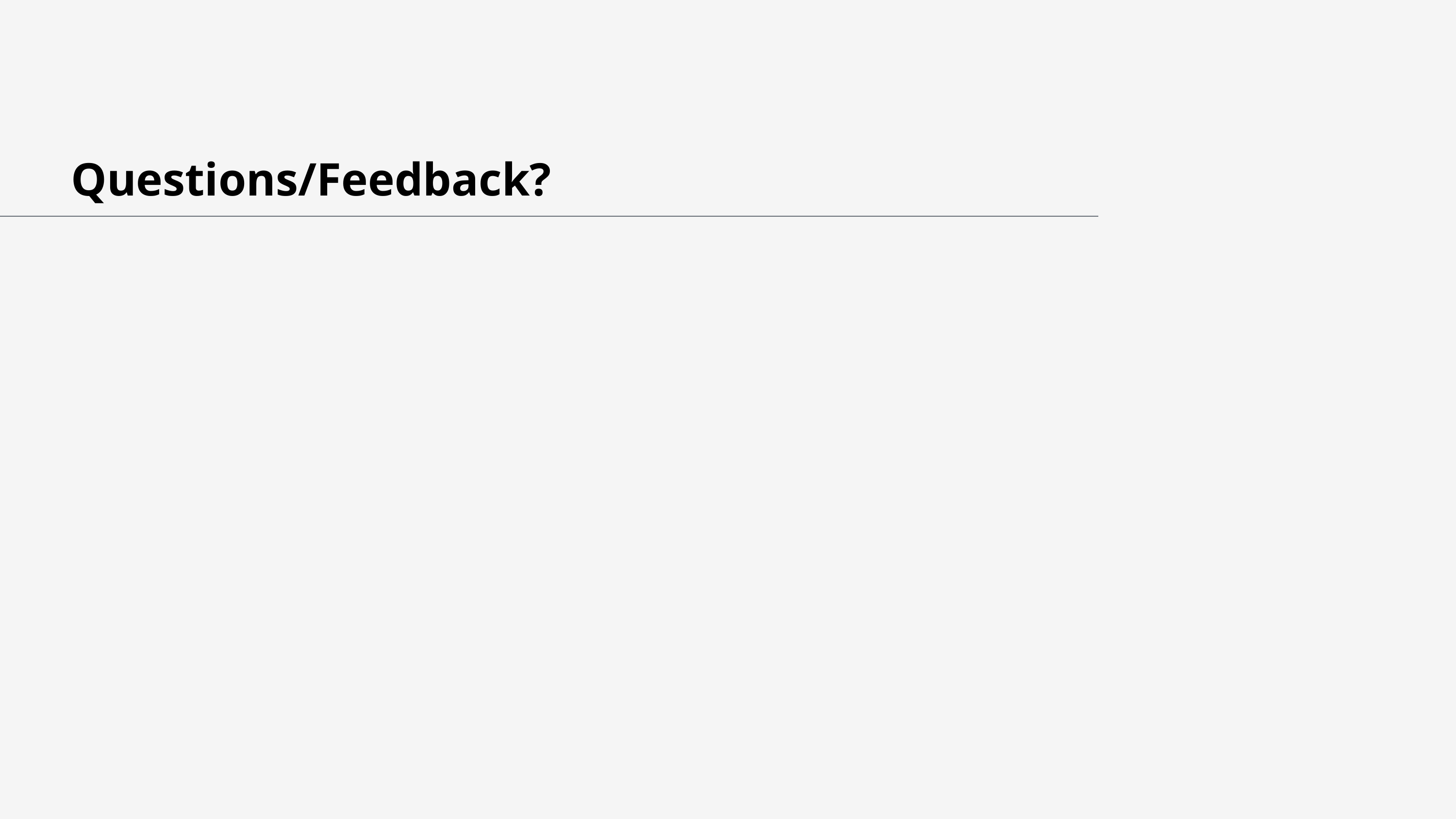

Questions/Feedback?

## Slide 21
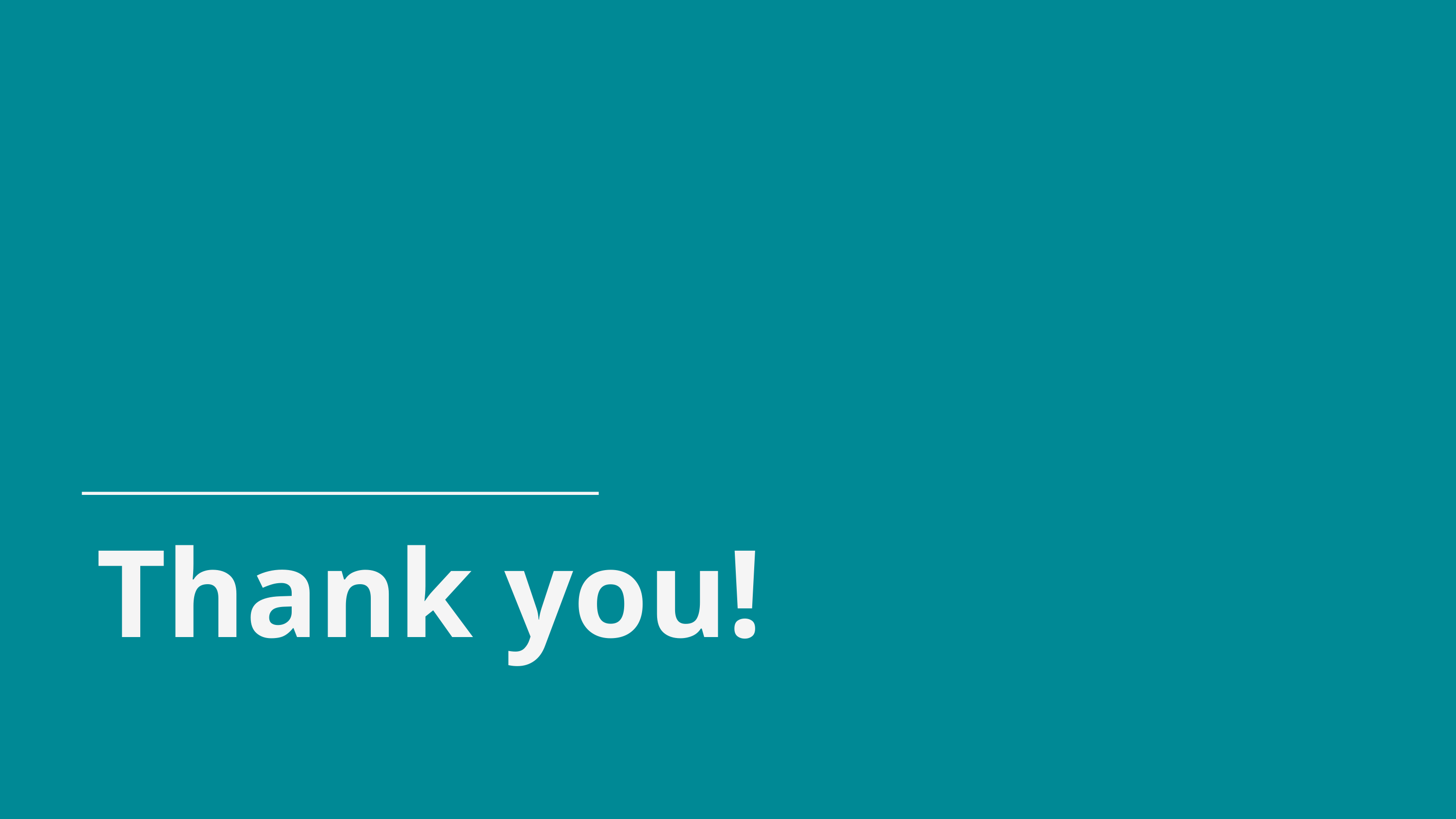

Thank you!
