# Supplementary material for: A Trauma-informed Care Curriculum for Perinatal Providers, Staff, and Learners
Source: MedEdPORTAL. 2025 Dec 9;21:11563. doi: 10.15766/mep_2374-8265.11563 (PMC12686155; doi:10.15766/mep_2374-8265.11563)
Supplement: Supplementary file 1 — Part 1 - Overview of TIC.pptxPart 2 - TIC in Perinatal Care.pptxPart 3 - Vicarious Trauma.pptxPart 4 - Community Voices & Reflection.pptxPresurvey.docxPostsurvey.pdf [file mep_2374-8265.11563-s001.zip › D. Part 4 - Community Voices & Reflection.pptx]

## Slide 1
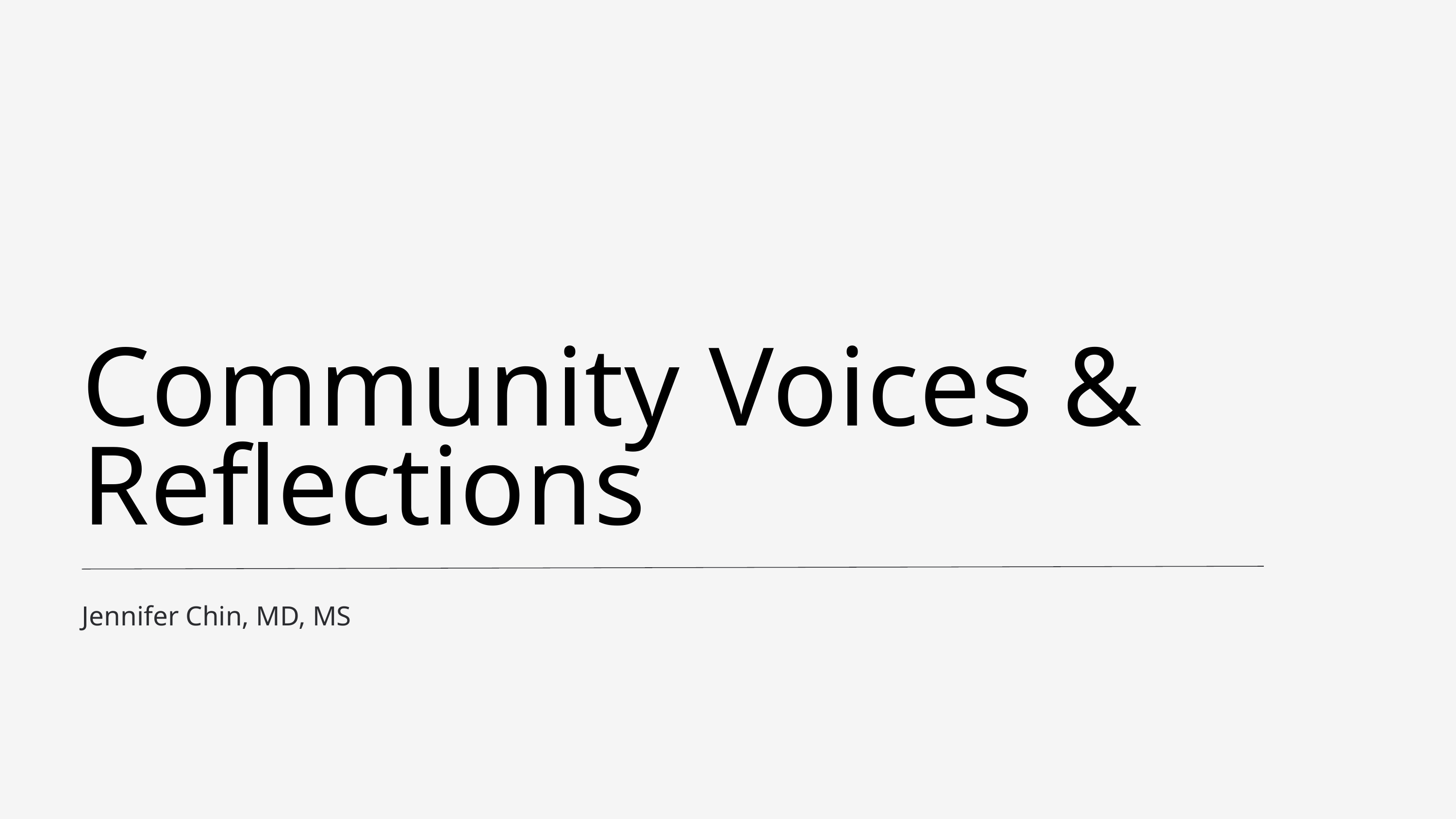

Community Voices & Reflections
Jennifer Chin, MD, MS

## Slide 2
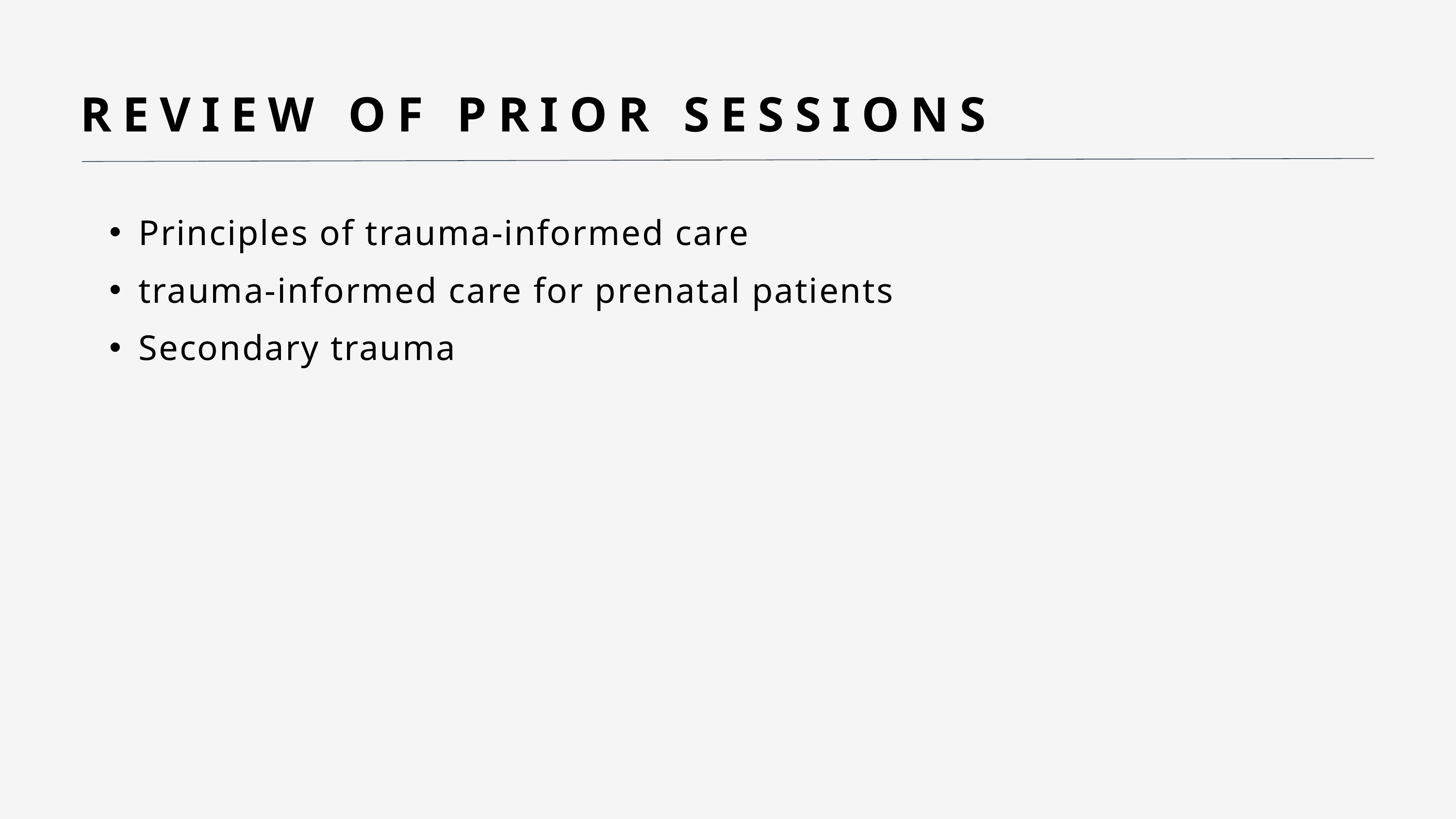

REVIEW OF PRIOR SESSIONS
Principles of trauma-informed care
trauma-informed care for prenatal patients
Secondary trauma

## Slide 3
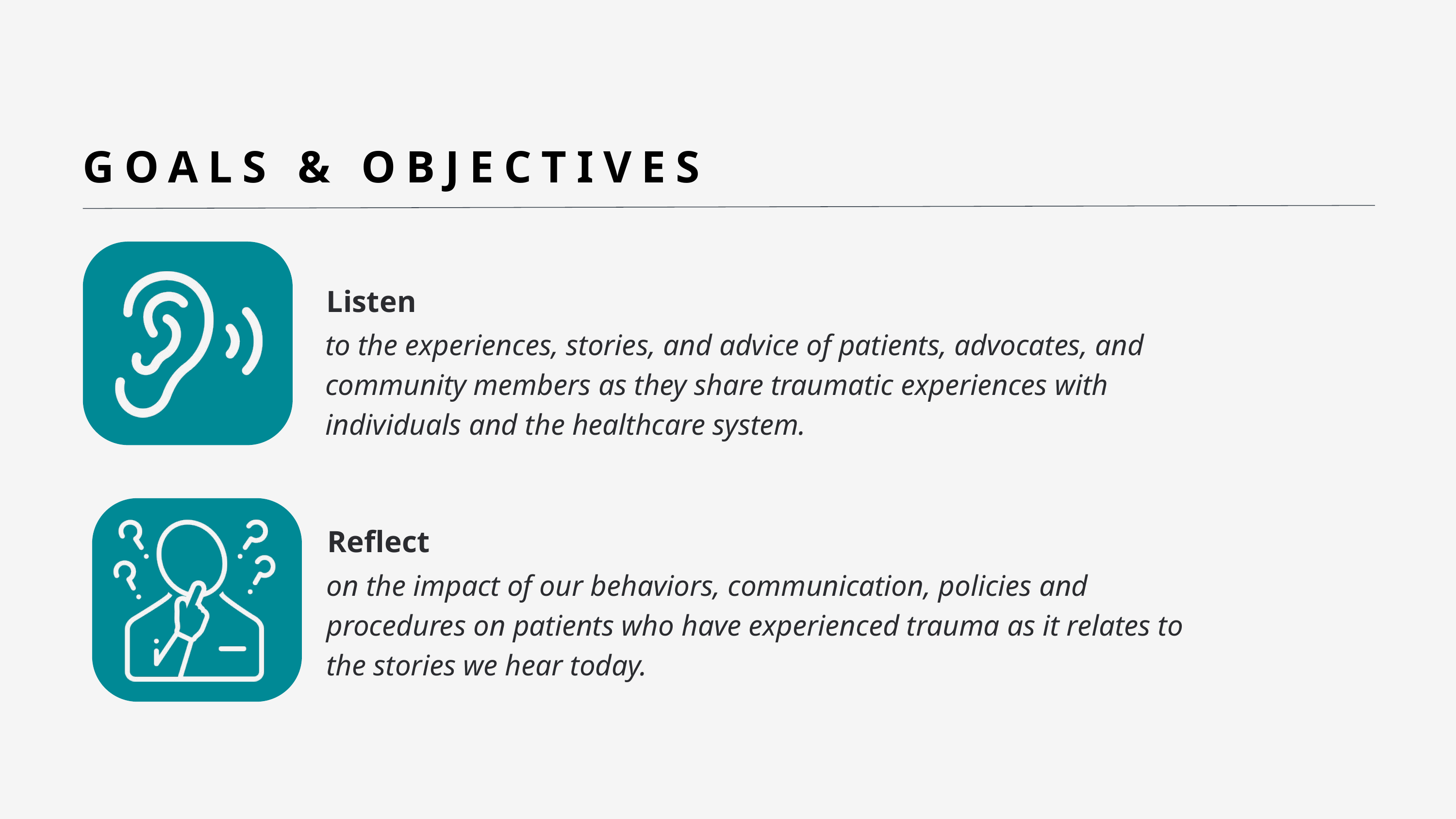

GOALS & OBJECTIVES
Listen
to the experiences, stories, and advice of patients, advocates, and community members as they share traumatic experiences with individuals and the healthcare system.
Reflect
on the impact of our behaviors, communication, policies and procedures on patients who have experienced trauma as it relates to the stories we hear today.

## Slide 4
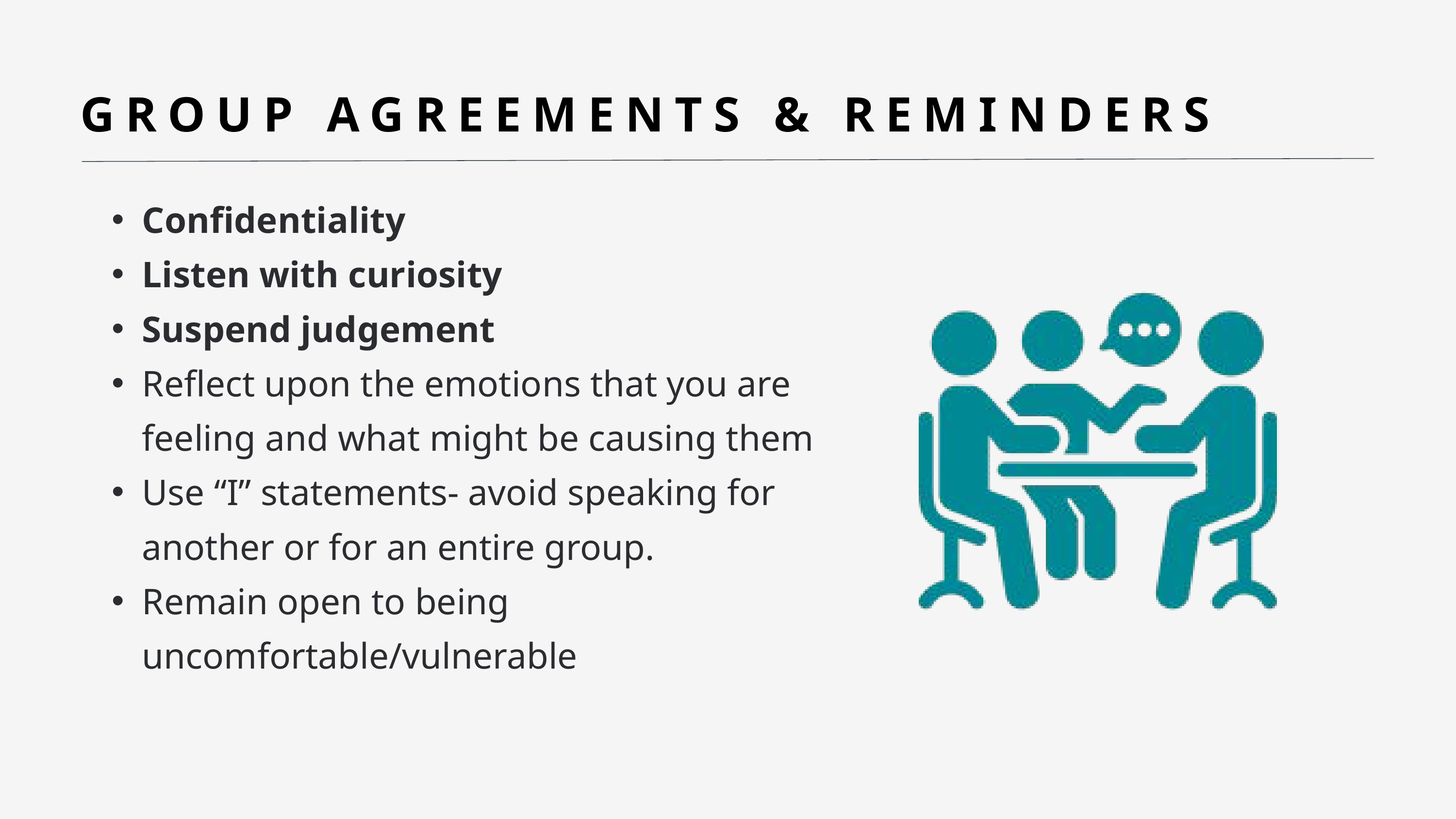

GROUP AGREEMENTS & REMINDERS
Confidentiality
Listen with curiosity
Suspend judgement
Reflect upon the emotions that you are feeling and what might be causing them​
Use “I” statements- avoid speaking for another or for an entire group. ​
Remain open to being uncomfortable/vulnerable

## Slide 5
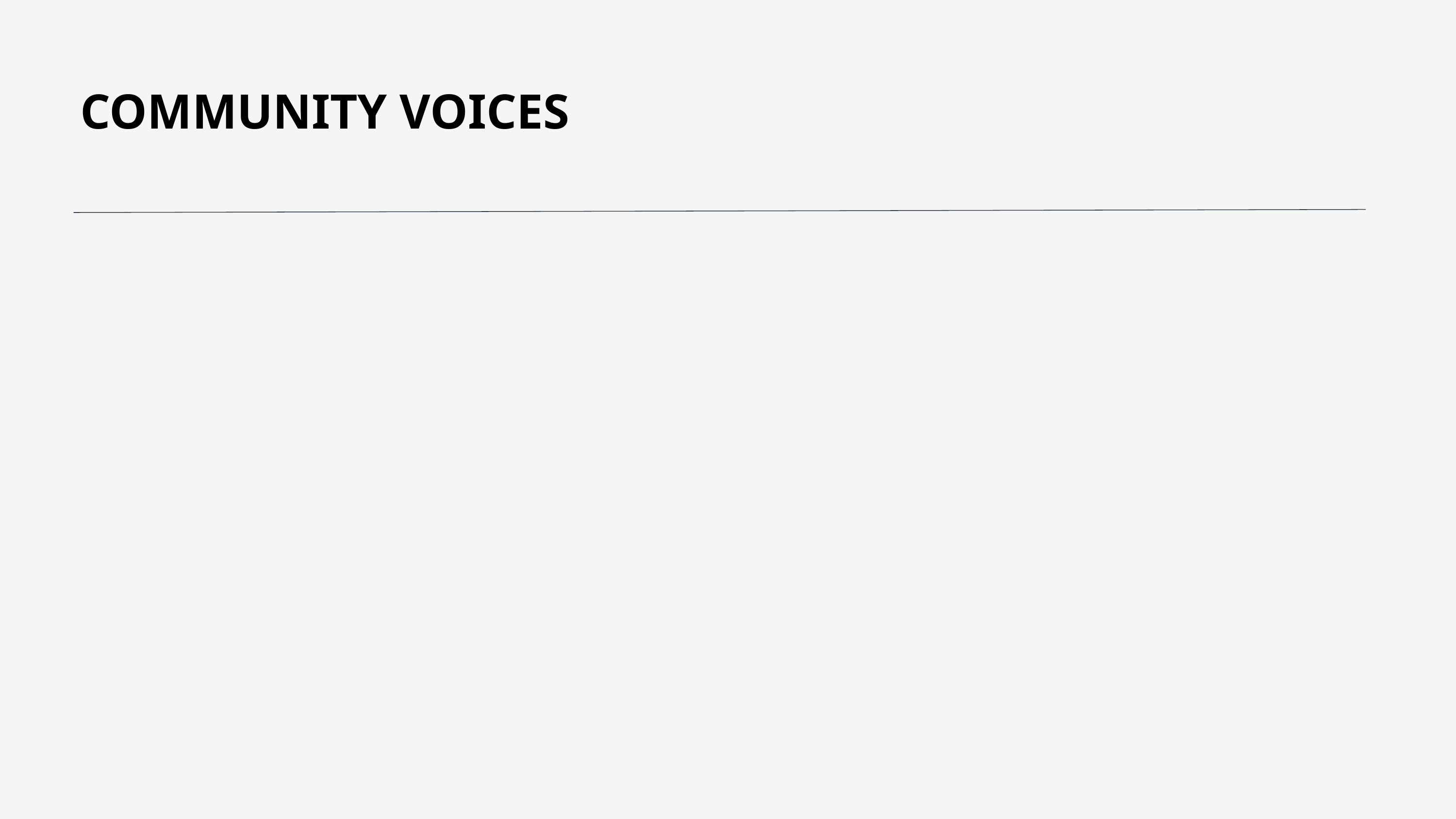

COMMUNITY VOICES

## Slide 6
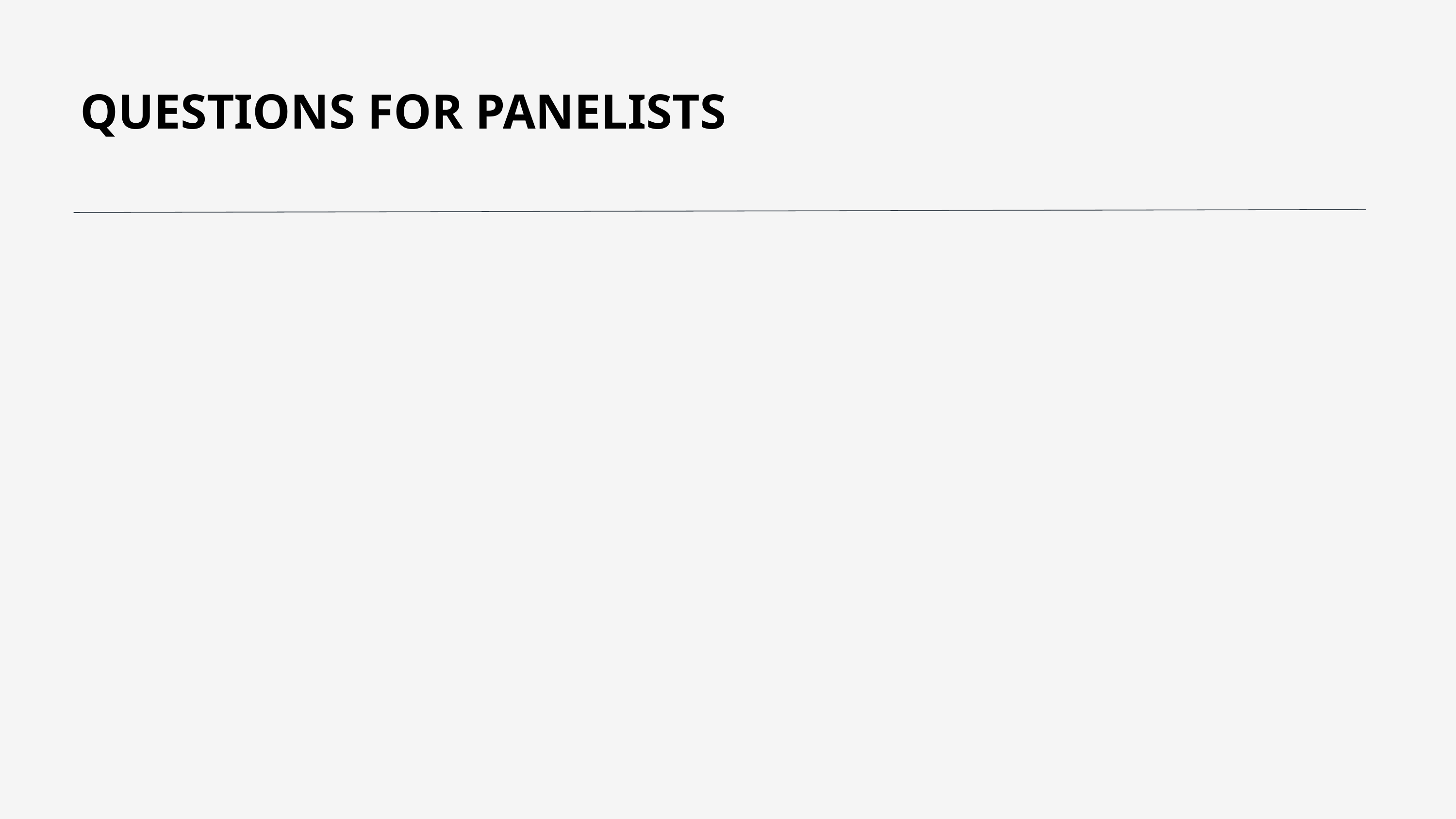

QUESTIONS FOR PANELISTS

## Slide 7
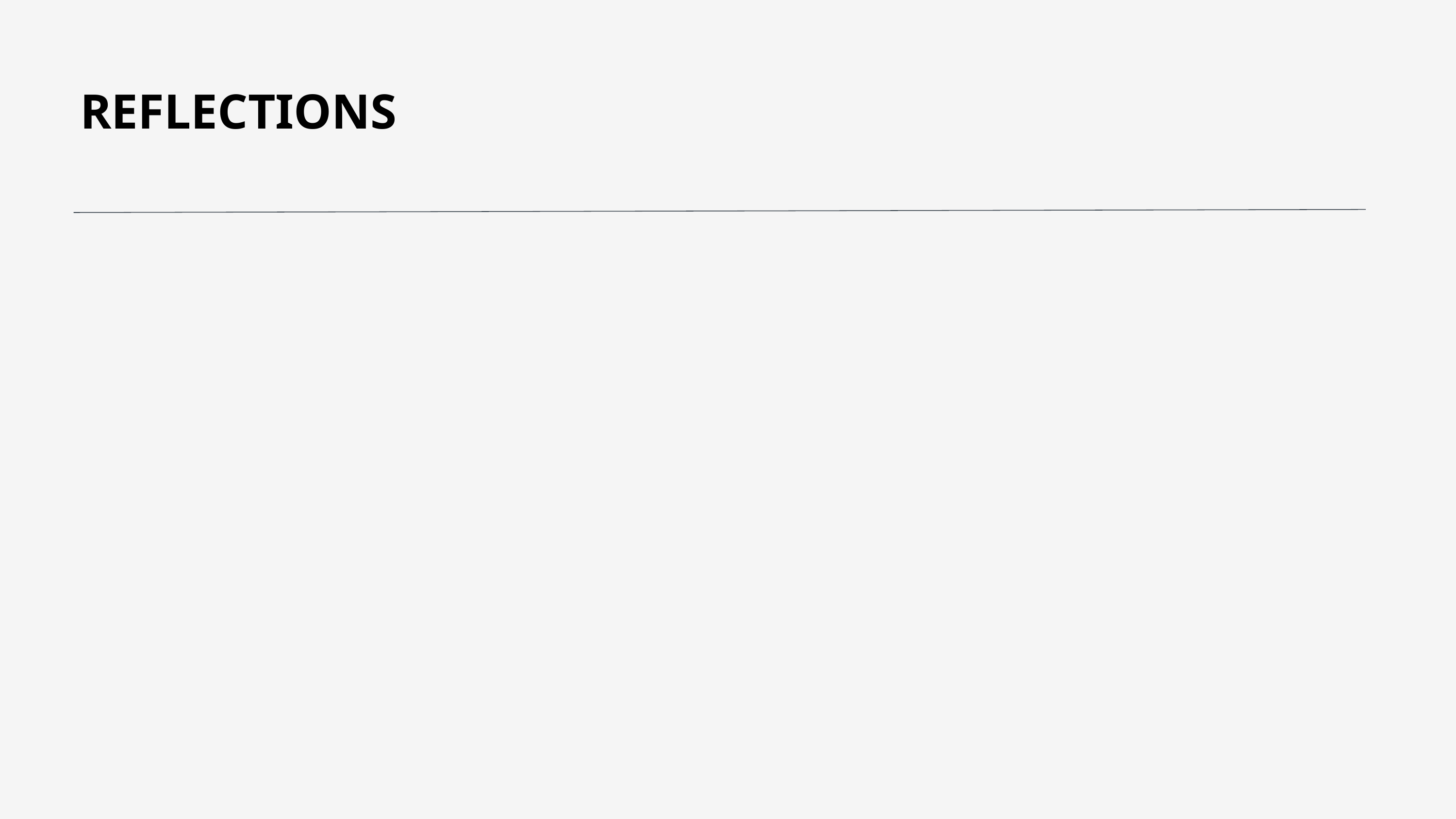

REFLECTIONS
