# Supplementary material for: A Trauma-informed Care Curriculum for Perinatal Providers, Staff, and Learners
Source: MedEdPORTAL. 2025 Dec 9;21:11563. doi: 10.15766/mep_2374-8265.11563 (PMC12686155; doi:10.15766/mep_2374-8265.11563)
Supplement: Supplementary file 1 — Part 1 - Overview of TIC.pptxPart 2 - TIC in Perinatal Care.pptxPart 3 - Vicarious Trauma.pptxPart 4 - Community Voices & Reflection.pptxPresurvey.docxPostsurvey.pdf [file mep_2374-8265.11563-s001.zip › E. Presurvey.docx]

Pre-Workshop Survey

Please complete the survey below.Thank you!

Would you like to receive a gift card after completion Yes


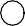

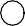


of this survey? No

Email (This will not be shared with anyone outside the

research team and will only be used to contact you for a gift card if you desire and the post-workshop

survey.)

Age

Transgender Yes


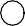

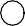

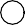


No

Prefer not to respond

Gender identity Woman


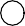

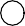

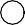

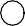

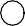

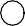

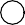


Man

Non-binary/non-conforming Agender Genderqueer/genderfluid Prefer not to respond

Prefer to self-identify (please indicate)

Other gender identity

Sexual orientation Heterosexual/straight


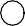

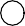

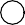

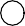

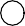


Gay/lesbian Bisexual

Prefer not to answer

Prefer to self-identify (please indicate)

Other sexual orientation

Race White


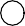

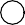

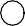

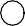

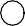

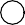

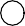

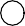


Black Asian

Native Hawaiian/Pacific Islander American Indian/Alaskan Native Prefer not to respond Multiracial (please indicate) Other (please indicate)

Multiracial

Other race

Asian (select all that apply)
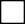
 Indian


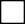
 Chinese
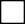
 Filipino
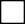
 Japanese
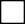
 Korean


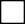
 Vietnamese


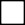
 Other Asian (please indicate)

Other Asian

Native Hawaiian/Pacific Islander (select all that
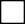
 Native Hawaiian

apply)
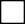
 Guamanian/Chamorro


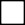
 Samoan


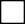
 Other Pacific Islander (please indicate)

Other Native Hawaiian/Pacific Islander

Ethnicity Hispanic/Latinx


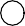

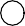

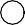

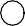


Not Hispanic/Latinx Prefer not to respond Other (please indicate)

Other ethnicity

How would you describe your disability/ability I do not identify with a disability or impairment. identity? We are interested in this identification My disability/ability identity includes one or regardless of whether you typically request more of the following: a sensory impairment


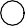

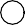


accommodations for this disability. (vision or hearing); learning disability (e.g.

ADHD, dyslexia); long-term medical illness (e.g. epilepsy, cystic fibrosis); mobility impairment; mental health disorder; or a disability or impairment not listed above.


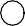
 Prefer not to respond

Number of years working in pregnancy care

Job responsibility/role in prenatal care or inpatient MD/DO Attending


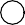

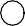

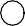

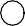

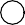

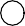

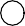

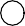

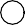

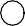

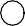

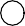

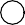


obstetrical care MD/DO Fellow

MD/DO Resident Midwife

ARNP PA MA RN MSW

Pharmacist Front Desk Staff Manager

Other (please indicate)

Other job

Have you had any prior training or education in TIC? Yes No


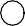

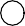


Describe the training (please include length of training, topics covered, and institution if applicable)

I believe I will learn a great deal from this program. Strongly disagree


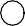

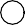

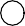

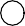

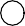


Disagree Neutral Agree

Strongly agree

I feel that I know what TIC is. Strongly disagree Disagree


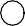

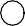

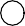

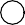

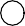


Neutral Agree

Strongly agree

It is important for people in my role to practice TIC. Strongly disagree


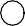

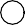

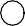

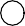

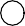


Disagree Neutral Agree

Strongly agree

Trauma-informed practice may improve interactions Strongly disagree within teams. Disagree


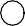

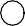

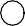

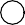

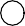


Neutral Agree

Strongly agree

Trauma-informed practice may enhance self-care. Strongly disagree Disagree


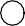

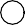

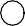

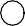

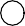


Neutral Agree

Strongly agree

TIC requires both individual and systems change. Strongly disagree Disagree


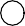

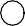

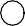

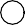

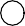


Neutral Agree

Strongly agree

Information about TIC is relevant to our Strongly disagree


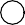

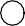

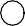

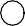

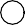


clients/patients. Disagree

Neutral Agree

Strongly agree

Our clients/patients will benefit from receiving TIC. Strongly disagree Disagree


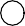

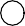

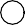

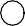

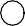


Neutral Agree

Strongly agree

TIC will help our clients/patients have better health Strongly disagree


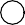

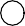


outcomes. Disagree

Neutral Agree

Strongly agree

I understand how a trauma history and post-traumatic Strongly disagree stress disorder can affect pain during labor and Disagree

birth. Neutral

Agree Strongly agree

I know how to identify symptoms of post-traumatic Strongly disagree

stress, including dissociation, in clients/patients. Disagree Neutral Agree

Strongly agree

I know how to address traumatic situations in labor Strongly disagree for someone who has experienced trauma in the past. Disagree

Neutral Agree

Strongly agree

I plan to use strategies presented in this program in Strongly disagree caring for and interacting with clients/patients who Disagree

are trauma survivors. Neutral

Agree Strongly agree

I know how to identify trauma caused by caring for Strongly disagree

those who have experienced trauma (i.e. vicarious Disagree

trauma). Neutral

Agree Strongly agree

I have a good understanding of how certain populations Strongly disagree disproportionately experience trauma. Disagree

Neutral Agree

Strongly agree

What do you most hope to learn from this workshop?

What barriers do you perceive will hinder your ability

to implement the knowledge gained from this workshop?

How often do you include trauma-informed principles in Every patient

your work right now? Every day but not with every patient Every week

Every month

Less often than once a month
