# Supplementary material for: A Trauma-informed Care Curriculum for Perinatal Providers, Staff, and Learners
Source: MedEdPORTAL. 2025 Dec 9;21:11563. doi: 10.15766/mep_2374-8265.11563 (PMC12686155; doi:10.15766/mep_2374-8265.11563)
Supplement: Supplementary file 1 — Part 1 - Overview of TIC.pptxPart 2 - TIC in Perinatal Care.pptxPart 3 - Vicarious Trauma.pptxPart 4 - Community Voices & Reflection.pptxPresurvey.docxPostsurvey.pdf [file mep_2374-8265.11563-s001.zip › F. Postsurvey.pdf]

Post-Workshop Survey

Please complete the survey below.Thank you!

Did you attend or listen to a recording of Session 1?	◯ Attended live<br>◯ Listened to recording<br>◯ Did not attend or listen
Did you attend or listen to a recording of Session 2?	◯ Attended live<br>◯ Listened to recording<br>◯ Did not attend or listen
Did you attend or listen to a recording of Session 3?	◯ Attended live<br>◯ Listened to recording<br>◯ Did not attend or listen
Did you attend or listen to a recording of Session 4?	◯ Attended live<br>◯ Listened to recording<br>◯ Did not attend or listen
I learned a great deal from this program.	◯ Strongly disagree<br>◯ Disagree<br>◯ Neutral<br>◯ Agree<br>◯ Strongly agree
I feel that I know what TIC is.	◯ Strongly disagree<br>◯ Disagree<br>◯ Neutral<br>◯ Agree<br>◯ Strongly agree
It is important for people in my role to practice TIC.	◯ Strongly disagree<br>◯ Disagree<br>◯ Neutral<br>◯ Agree<br>◯ Strongly agree
Trauma-informed practice may improve interactions within teams.	◯ Strongly disagree<br>◯ Disagree<br>◯ Neutral<br>◯ Agree<br>◯ Strongly agree
Trauma-informed practice may enhance self-care.	◯ Strongly disagree<br>◯ Disagree<br>◯ Neutral<br>◯ Agree<br>◯ Strongly agree
TIC requires both individual and systems change.	◯ Strongly disagree<br>◯ Disagree<br>◯ Neutral<br>◯ Agree<br>◯ Strongly agree

Information about TIC is relevant to our clients/patients.	◯ Strongly disagree<br>◯ Disagree<br>◯ Neutral<br>◯ Agree<br>◯ Strongly agree
Our clients/patients will benefit from receiving TIC.	◯ Strongly disagree<br>◯ Disagree<br>◯ Neutral<br>◯ Agree<br>◯ Strongly agree
TIC will help our clients/patients have better health outcomes.	◯ Strongly disagree<br>◯ Disagree<br>◯ Neutral<br>◯ Agree<br>◯ Strongly agree
I understand how a trauma history and post-traumatic stress disorder can affect pain during labor and birth.	◯ Strongly disagree<br>◯ Disagree<br>◯ Neutral<br>◯ Agree<br>◯ Strongly agree
I know how to identify symptoms of post-traumatic stress, including dissociation, in clients/patients.	◯ Strongly disagree<br>◯ Disagree<br>◯ Neutral<br>◯ Agree<br>◯ Strongly agree
I know how to address traumatic situations in labor for someone who has experienced trauma in the past.	◯ Strongly disagree<br>◯ Disagree<br>◯ Neutral<br>◯ Agree<br>◯ Strongly agree
I plan to use strategies presented in this program in caring for and interacting with clients/patients who are trauma survivors.	◯ Strongly disagree<br>◯ Disagree<br>◯ Neutral<br>◯ Agree<br>◯ Strongly agree
I know how to identify trauma caused by caring for those who have experienced trauma (i.e. vicarious trauma).	◯ Strongly disagree<br>◯ Disagree<br>◯ Neutral<br>◯ Agree<br>◯ Strongly agree
I have a good understanding of how certain populations disproportionately experience trauma.	◯ Strongly disagree<br>◯ Disagree<br>◯ Neutral<br>◯ Agree<br>◯ Strongly agree
Did this program meet your personal objectives? What needs do you still have that we might address in future versions of this curriculum?	__________________________________

What barriers do you perceive will hinder your ability to implement the knowledge gained from this workshop?	__________________________________
Have you incorporated anything from these trainings into your practice?	◯ Yes<br>◯ No
If so, what?	__________________________________
How often do you include trauma-informed principles in your work right now?	◯ Every patient<br>◯ Every day but not with every patient<br>◯ Every week<br>◯ Every month<br>◯ Less often than once a month
Would you like to receive results of this study by email at the conclusion of this study?	◯ Yes<br>◯ No
If so, please provide your email	____________________________
Would you like to participate in an in-depth 30-45 minute follow-up interview focused on exploring participant experience with TIC training?	◯ Yes<br>◯ No
If so, please provide your best phone number	____________________________
